# Inter-individual variation of cellular and gene-expression properties of the human striatum

**DOI:** 10.64898/2026.03.20.713160

**Published:** 2026-03-23

**Authors:** Steven Burger, Olivia Yoo, James Nemesh, Ezra Muratoglu, Charles Vanderburg, Jiayi Yuan, Khalid Shakir, Curtis J. Mello, Nirmala A. Rayan, Julianna Milidantri, Kathleen Kim, Sadie Drouin, Emily Finn, Haoyuan Gao, Nikita Budnik, Melissa Goldman, Haley Fritch, Giulio Genovese, Marina Hogan, Olivia Catalini, Seva Kashin, Nicole Rockweiler, Alec Wysoker, Lauren Macaisa, Lucas Reese, Katelyn Flowers, Andrew W. Kraft, Stephen J. Fleming, Madelynn Coe, Riyaan Gunaratne, Liv Spina, Catherine Crombie, Abir Mohsin, Nolan Kamitaki, Evan Z. Macosko, Kiku Ichihara, Steven A. McCarroll

**Affiliations:** 1 Broad Institute of Harvard and MIT, Cambridge, MA, USA; 2 Department of Genetics, Harvard Medical School, Boston MA, USA; 3 Department of Neurology, Massachusetts General Hospital, Boston MA, USA; 4 Department of Psychiatry, Massachusetts General Hospital, Boston MA, USA; 5 Department of Neurobiology, Harvard Medical School, Boston MA, USA; 6 Howard Hughes Medical Institute, Boston MA, USA

## Abstract

The human brain varies from person to person in ways that shape behaviors and vulnerabilities, yet the cellular and molecular bases for inter-individual variation are largely unknown. Here we describe an analysis of cellular and gene-expression variation in four key structures of the striatum complex – the caudate, putamen, nucleus accumbens, and internal capsule – as well as the prefrontal cortex, from single-nucleus RNA-seq analysis of 3.9 million nuclei from 178 adult brain donors. We found that people with more astrocytes in any one brain region tended to have this property in all brain regions sampled; the same was true of striatal interneurons, microglia, and oligodendrocyte precursor cells (OPCs). OPCs showed attrition with age, declining in numbers by approximately 40% between age 30 and age 80 in both gray matter and white matter regions. We identified thousands of age-associated (but few sex-associated) variations in gene expression; the vast majority of these effects of age were cell-type-specific. Aging most strongly affected gene expression in projection neurons – especially striatal medium spiny neurons (MSNs/SPNs) – and had a much smaller effect on gene expression in interneurons. Individuals' ages could be predicted to within about five years based on RNA-expression patterns from any of the striatal cell types. Common genetic variants detectably affected the expression levels of some ten thousand genes; the great majority of these effects were cell-type-specific. These data will provide a foundation for exploring natural inter-individual variation, aging, and tissue-based studies of human brain vulnerabilities.

## Introduction

Human brains exhibit abundant biological variation – in size and activity patterns, in behavioral proclivities, and in vulnerabilities to illness. Several factors contribute to this biological variability. Humans, unlike isogenic laboratory animals, harbor much genetic variation: any two humans have tens of millions of genetic differences. Humans also live in diverse environments that shape brain development and function in many ways, through social context and through influences on nutrition and metabolism, stress-related neuroendocrine responses, and immune and inflammatory processes.

The striatum, as the principal input nucleus of the basal ganglia, is a key brain region in which biological variation may influence brain function. Striatal activity contributes to behavioral flexibility, reinforcement learning, decision making, motivation, and motor control^1-7^. The striatum is strongly influenced by neuromodulatory systems that can both reflect and shape inter-individual differences in molecular state and cellular activity across striatal circuits. These networks are involved in many neurological and psychiatric disorders, including obsessive compulsive disorder^8-10^ and pronounced neurodegeneration in Huntington's disease^11,12^.

Despite decades of anatomical and physiological investigation^13^, much of our current cellular and molecular understanding of the striatum is derived from rodent and non-human primate models, which have provided foundational insights into basal ganglia circuitry and cellular organization^14-19^ but do not address the ways in which such features might vary among individual humans. Significant work has been done to identify and characterize the distinct cell types within the striatum^20-24^, but there is still much to understand about how these cell types vary throughout the striatum, and from person to person. Recognizing and quantifying inter-individual variation in cell-type abundance and gene expression programs across the lifespan could in principle help provide a foundation for linking genetic variation to striatal circuit function and disease vulnerability. In this work, we sought to leverage advances in single-cell genomics to begin mapping some of the major axes along which the human striatum might commonly vary – including the cells that are present in the striatum, their relative abundances, and the genes they express. We also sought to identify anatomical features and age-related dynamics that reappear consistently across people.

Single-cell genomics generates detailed, multi-dimensional cellular and molecular profiles of any brain region or subregion; however, it has historically been challenging to determine which aspects of inter-individual variation are biological and which are technical. Small variations in how tissue specimens are subdissected, and how nuclei are extracted from tissue, can skew both the representations of different types of cells and the RNA transcripts that are ascertained for analysis. To address this, most studies have focused on how properties, including cell type composition and gene expression profiles, differ on average between two groups of individuals: for example, those with a specific clinical condition and those without. Less-emphasized – in part because it often involves an unknown mix of technical, statistical, and biological factors – is the large quantitative variation that appears within each such group.

We sought to address these challenges using two aspects of project design. The first was to analyze several brain regions in each of the individuals sampled; this allowed us to identify cellular and molecular features that were consistent properties of an individual in the sense that they appeared in most or in all brain regions under analysis. The second was to perform all experiments in a well-controlled "village" format^25^, in which tissue samples from ~20 donors were processed together and later reassigned to their donor of origin using transcribed genetic variants. This approach made it straightforward to recognize and correct technical effects on the resulting data, since such effects were shared by all of the specimens in an experiment.

Here, and in concert with broader efforts by the BICAN consortium, we applied this approach to deeply analyze the main structures of the striatum – the caudate (CaH), putamen (Pu), and nucleus accumbens (NAC) – which share major cell types in common, but differ in functional connectivity^26,27^, gene expression^28,29^, and disease vulnerability^12,30-33^. We also included the internal capsule (ic), a white matter tract between the caudate and putamen, and dorsolateral prefrontal cortex (DFC), a well-characterized cortical area, to help recognize properties that extend beyond the striatum. Neuroanatomical structures and region abbreviations are defined in the Allen Institute Developing Human Brain Atlas (DHBA) ontology^34^ (RRID:SCR_027940).

We created a resource of single-nucleus RNA-seq profiles from 3.9 million nuclei sampled from 178 adult human brain donors. We identified many cellular features that show a surprising degree of quantitative variation from person to person; many of these features reappeared throughout a person's various brain regions or showed clear relationships to other variables (such as age and common genetic variation), suggesting that they embody true inter-individual variation in human neurobiology. We discuss below ways to use this data resource and the many patterns it contains, and provide a web-based portal (RRID:SCR_028065) to make the data simple to query and utilize in other work. This resource will enable a deep exploration of inter-individual variation and the ways it is shaped by age and genetics, and will serve as a resource for conceiving and designing future tissue-based studies of human neurobiology.

## Results

### A single-cell resource of inter-individual variation in the adult human striatum

We analyzed brain tissues that had been donated at the end of life by 178 persons. These included a core donor set (n = 147) and additional donors (n = 31) for whom one or more clinical or neuropathological findings became apparent in the course of the project (Table S1). Below we specify which analyses use the core set of donors and which use the extended (core + additional) set. Donors were selected based on donor characterization by brain bank staff, assessments of tissue quality (see **Methods**), and availability of tissues from the regions of interest. The donor age distribution spanned from 27 to 90+ years old (median = 62; precise ages >89 were unavailable to protect donor identity), and included 111 male and 67 female donors (Figure S2B, Table S2).

**Figure 1.**
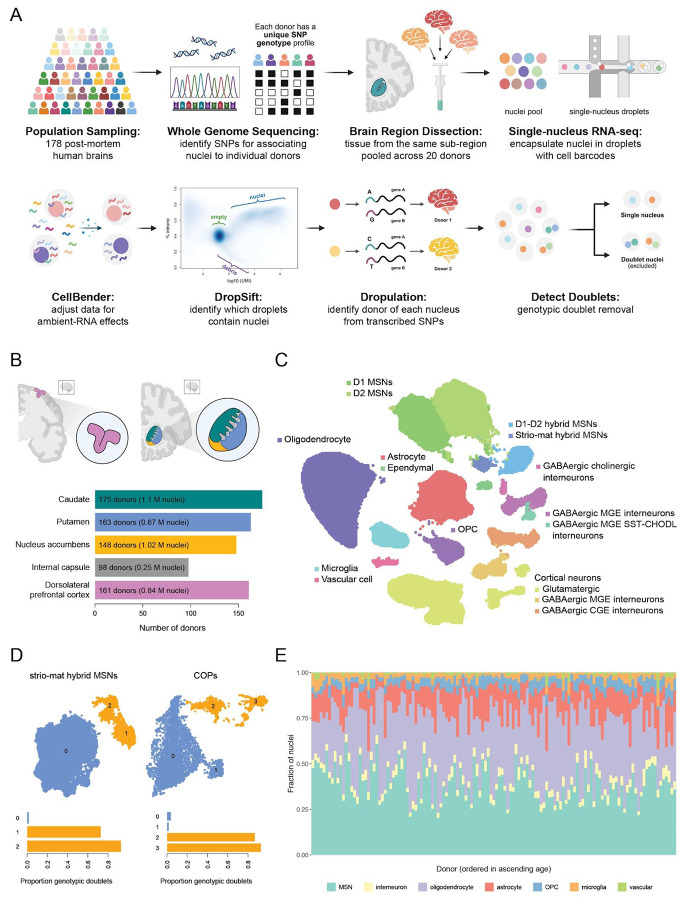
A single-cell resource for recognizing inter-individual variation in the adult human striatum. **A.** Schematic of data generation and processing. Top: multi-donor (“village”) snRNA-seq experimental workflow. Bottom: computational data processing workflow. Diagrams created with BioRender (RRID:SCR_018361). **B.** Brain regions sampled. Left: dorsolateral prefrontal cortex (pink). Right: caudate (green), putamen (blue), nucleus accumbens (yellow), and internal capsule (gray). Diagrams created with BioRender. Barplot represents the total number of donors sampled for each region. **C.** UMAP of all 3.9M nuclei assigned to a single donor and cell type. **D.** Genotypic (two-donor) doublet calls identified by Dropulation help confidently identify clusters of cell types that resemble mixtures of other cell types. Left: UMAP of strio-mat hybrid MSNs; clusters 1 and 2 contain high proportions of genotypic doublets (and are excluded from downstream analyses) compared to cluster 0. Right: UMAP of committed oligodendrocyte precursor (COP) clusters; clusters 2 and 3 have high proportions of genotypic doublets (and are excluded from downstream analyses). **E.** Stacked bar plot shows representation of major cell types among the caudate samples from 131 donors. Donors are ordered from left to right by ascending age. Major cell classes were detected in all donors sampled.

Variation between individuals is more subtle and quantitative than the categorical differences that distinguish cell types and species from one another. To better recognize and measure such variation, we implemented a "nuclei village" experimental framework^25^. Systematic dissections of the striatum separated caudate, putamen, nucleus accumbens, and internal capsule (see **Methods,** Figure S1). Tissues from each subregion were then processed in pools of ~20 donors; each such pool ("village") was handled as a single sample through nuclei isolation and snRNA-seq (Figure 1A). Donor identity for each nucleus was recognized using combinations of thousands of transcribed sequence variants that appear on RNA transcripts, using a computational approach (Dropulation; RRID:SCR_018142) that we have previously described^35^.

Gene-expression measurements were computationally adjusted for ambient RNA contamination using CellBender^36^ (RRID:SCR_025990). The selection of cell barcodes that contained nuclei (as opposed to empty droplets and droplets containing cellular debris) was refined using DropSift^37^ (RRID:SCR_028040; see **Methods**), a tool we recently developed that utilizes the fraction of reads that are intron-derived (alongside total UMI counts) to recognize the profiles of nuclei, in a data-driven manner that adapts to each dataset. Genotypic (two-donor) doublets were identified with Dropulation.

Using this approach, single-nucleus RNA-seq (snRNA-seq) data were generated from caudate head (n = 175), putamen (n = 163), nucleus accumbens (n = 148), internal capsule (n = 98), and dorsolateral prefrontal cortex (n = 161), with multiple regions sampled from the same donors where available (Figure 1B, S2A).

Unsupervised clustering of the single-nucleus RNA-expression profiles identified all major neuronal and non-neuronal cell classes across the regions sampled, including the diverse types of medium spiny neurons (MSNs, also known as striatal projection neurons or SPNs), the principal neuronal population of the striatum (Figure 1C, S2D). Manual cluster annotations utilized cell type labels from the consensus basal ganglia taxonomy^38^ generated via MapMyCells^39^ (RRID:SCR_024672) (Figure S2E). To identify the smaller number of remaining doublets (in which both nuclei are from the same donor), the far larger number of genotypic doublets were used to recognize the gene expression patterns associated with doublets in general. This systematic removal of doublets facilitated the confident recognition of cell types with transcriptional profiles that happen to somewhat resemble mixtures of other prominent cell types — including D1-D2 hybrid medium spiny neurons (MSNs), striosome-matrix hybrid MSNs, and committed oligodendrocyte precursors (COPs), which express both OPC (oligodendrocyte precursor cells) and oligodendrocyte genes — when these were observed in clusters that contained few, if any, genotypic doublets (Figure 1D).

Following data quality filtering and removal of doublets, the final dataset comprised approximately 3.9 million nuclei. For each brain region, a small fraction of the subdissected specimens (generally about 15%) exhibited low ascertainment of nuclei and/or highly abnormal cell type proportions and were excluded from downstream analyses (see **Methods**). Expected neuronal and glial cell types were ascertained in tissue samples from all donors (Figure 1E). Following quality control of the data, we first looked at changes in cell type abundances across regions, then the variables that might relate to these changes (age, sex), and finally at effects of common genetic variation on gene expression in these striatal and cortical cell populations.

### Glial cell abundances vary from person to person

**Figure 2.**
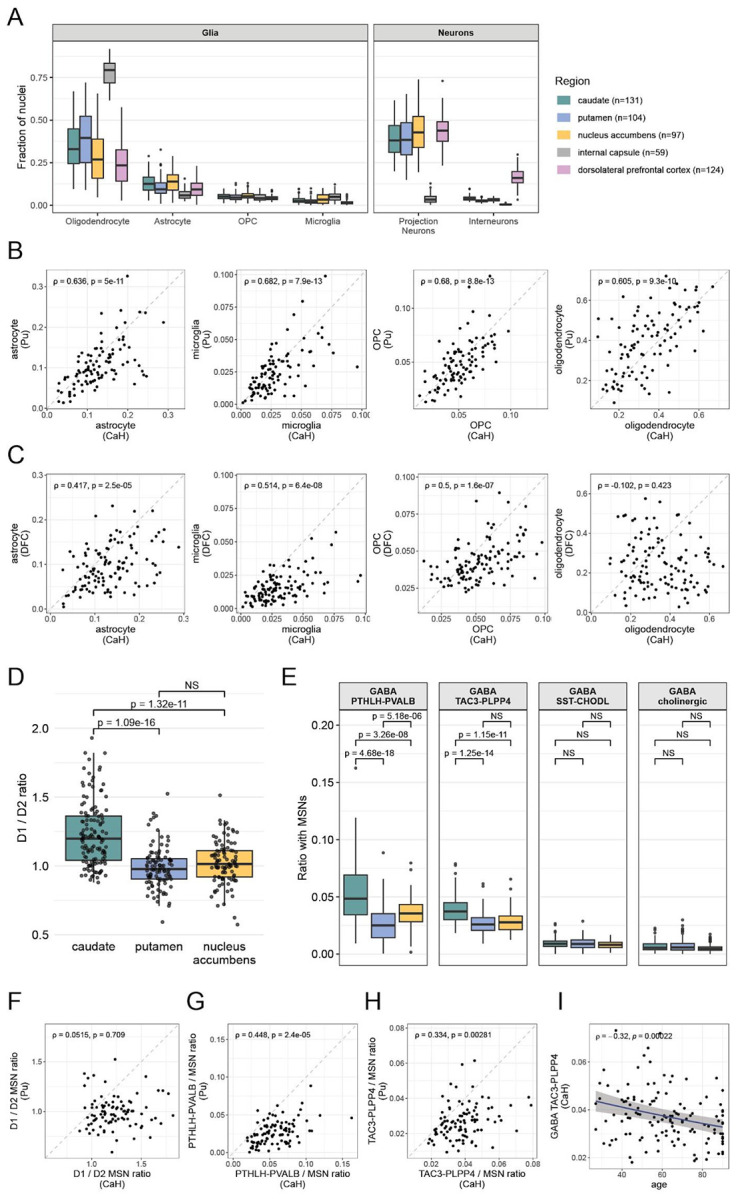
Variation in cell type abundances across individuals and between anatomical regions. (Extended analyses are in Figures S3-S7). **A.** Representation of major cell types in the brain regions sampled. Boxes indicate the interquartile range across donors, center lines denote median values, and whiskers extend to the most extreme points within 1.5x the interquartile range. Relative to the gray matter regions, the internal capsule yielded more oligodendrocyte and fewer neuronal nuclei; DFC had a higher fraction of interneurons compared to striatal regions. **B.** Representation of glial cell type abundances (as a fraction of all nuclei sampled) in the caudate (x-axis) and putamen (y-axis) of 98 donors. Spearman correlation coefficient and corrected p-values are shown (Benjamini-Hochberg (BH) procedure, n=498 tests). **C.** Same as **B**, for 114 donors sampled in caudate (x-axis) and dorsolateral prefrontal cortex (y-axis). **D.** D1/D2 MSN ratio was significantly higher in CaH (n=131 donors) than in Pu (n=104 donors) and NAC (n=97 donors); nominal p-values are from the Wilcoxon rank-sum test. No significant difference was detected between Pu and NAC. **E**. Quantitative representation of striatal interneuron subtypes relative to the number of MSNs. Interneuron abundances are expressed relative to MSNs to mitigate the effects of inter-individual variation in glial abundances (panel **B**) on these calculations. Statistical significance was assessed using the Wilcoxon rank-sum test and corrected for multiple comparisons (BH procedure, 12 tests). **F.** Ratio of the number of D1 MSNs to D2 MSNs in CaH (x-axis) and Pu (y-axis) samples from 98 donors. Nearly all donors have a greater D1/D2 MSN ratio in CaH than Pu. This ratio was not correlated between CaH or Pu. Spearman rank correlation coefficient and corrected p-value shown (BH procedure, n=498 tests). **G.** Representation of GABAergic PTHLH-PVALB interneuron abundances (relative to MSNs) in the caudate (x-axis) and putamen (y-axis) of 98 donors. Spearman correlation coefficient and corrected p-values are shown (BH procedure, n=498 tests). **H.** Same as **G**, but for GABAergic TAC3–PLPP4 interneurons. **I.** Abundance of TAC3–PLPP4 interneurons declines with age across striatal regions, shown for CaH. Abundance is expressed as a fraction of all neurons sampled. Each point represents a donor; the blue lines indicate beta-binomial fit with 95% confidence intervals shown as gray ribbons. Abundance was negatively correlated with age for all brain regions (Spearman correlation coefficient and nominal p-value shown). Modeling with a beta-binomial regression confirms a significant decline with age (β = −0.047 per decade; 95% CI: −0.069 to −0.025; BH-adjusted p-value = 0.001; 44 tests; Table S4).

Cell type proportions exhibited extensive variation among the individual donors (Figure 2A). A key question involves whether such variation (at least in part) reflects true biological variation among the brain donors sampled, as opposed to experimental noise introduced by variation in tissue sampling, such as differences in microdissections and tissue micro-environments.

The analysis of microdissections from multiple brain regions in the same large panel of donors made it possible to ask whether this variability reflected intrinsic donor-specific features shared across brain regions. To exclude effects of clinically apparent conditions or conditions apparent to conventional neuropathological analyses of the specimens, we used the stringent donor group (n = 147) for these analyses.

We found that representations of the major glial cell types (astrocytes, microglia, OPCs, and oligodendrocytes) were significantly correlated from one striatal region to another: donors for whom the abundance of one cell class was high in the caudate tended to have this property in the putamen and nucleus accumbens as well (Figure 2B, S3A). These relationships arose from the entire set of donors, as opposed to a few outliers, and were highly significant even when assessed with conservative, non-parametric statistical tests (Figure 2B, S3A).

These relationships also extended beyond the striatum: the tendency of the individual donors to have relatively high or low abundances of astrocytes (or microglia, or OPCs) was also apparent in their internal capsule (Figure S3B) and even in their dorsolateral prefrontal cortex (Figure 2C). These correlations were similarly strong when the abundance of each cell type was calculated relative to the number of neurons rather than the total number of nuclei (to prevent the glial cell types from affecting one another's abundance estimates; Figure S4). These results suggest that a substantial component of inter-individual differences in glial abundance reflects biological properties of individuals, and that these properties also extend beyond the striatum.

Substantial fractions of inter-individual variation in the measured representations of OPCs, astrocytes, and microglia in the caudate could be predicted by measurements of the same properties in DFC (Pearson *r*^*2*^ of 0.22 for OPCs, 0.18 for astrocytes, and 0.29 for microglia). Focusing on the component of inter-individual variation that was shared between DFC and caudate, we conservatively estimated that caudate samples from any two individuals will frequently (in at least half of comparisons) have at least a 1.2 fold difference in the representation of astrocytes and OPCs, and a 1.4 fold difference in the representation of microglia (see **Methods**).

The principal exception to this inter-region correlation pattern involved oligodendrocytes (Pearson r^2^ = 0.01 between caudate and DFC). Across the individual donors, their abundances in the DFC (or ic) were not correlated with those in the striatum (CaH, Pu, and NAC) (Figure 2C, S3B), and thus appear to be shaped by differential sampling of white matter and/or axonal fiber bundles in sub-dissections.

These results suggest that, even among persons not known to have brain-related clinical conditions or neuropathological findings, the tendency of individual people to have higher-than-average or lower-than-average numbers of astrocytes, microglia, and/or OPCs is a consistent property of the individual that appears across multiple striatal and cortical regions.

### Neuronal proportions differ among striatal regions

Sampling from a large number of donors in multiple brain regions made it possible to recognize quantitative features of specific striatal regions that consistently differed from each other, in many different people. In order to minimize the effect of the large inter-individual variation in the glial cell types (Figure 2), we measured the abundances of neuronal types relative to those of other types of neurons.

MSNs are classically categorized based on their expression of either D1 or D2 dopamine receptors^40^, corresponding to the direct (D1) and indirect (D2) output pathways to basal ganglia structures. D1 and D2 MSNs are further distributed across two anatomical compartments in the striatum: the matrix and striosomes^41-43^ (Figure S5). The observed ratio of D1 to D2 MSNs was 20% higher in caudate (mean 1.23) than in the putamen (mean 1.00) and nucleus accumbens (mean 1.02) (Figure 2D). This difference was consistent across both matrix and striosome MSNs (Figure S6A) and across donors (Figure S6B), and aligns with findings from mouse immunofluorescence studies^44,45^. This finding also aligns with results from the contemporaneous discovery (from Slide-tags spatial transcriptomics data) that six discrete spatial zones underlie variation across the striatum: the two zones with the greatest representation of D1 MSNs overlap with the caudate^46^. The ratio of striosome to matrix MSNs did not differ significantly between caudate and putamen, with striosome MSNs representing on average 17% of MSNs in both brain regions (Figure S6C).

The striatum also contains populations of non-canonical (also referred to as “hybrid” or “eccentric”) MSNs^22,47^. The ratio of eccentric to canonical MSNs consistently distinguished the nucleus accumbens (in which approximately 19% of MSNs were of the eccentric subtypes) from the caudate and putamen (approximately 7%) (Figure S6D), largely due to the elevated abundance of D1 NUDAP (neurochemically unique domains of accumbens and putamen^48^) MSNs (Figure S5F,G).

While MSNs are the principal neuronal population in the striatum, they are accompanied and modulated by a heterogeneous set of GABAergic interneurons with distinct structural and electrophysiological properties^49-51^. The two most abundant striatal interneuron classes are the fast-spiking PTHLH-PVALB and the TAC3-PLPP4 types, the latter previously considered primate-specific^17^ but recently detected across several mammalian species^52^. PTHLH-PVALB interneurons were most numerous in caudate, followed by nucleus accumbens and then putamen, forming a medial-to-lateral gradient (Figure 2E). TAC3-PLPP4 interneurons were also most numerous in caudate but showed similar abundances in putamen and nucleus accumbens (Figure 2E).

**Figure 3.**
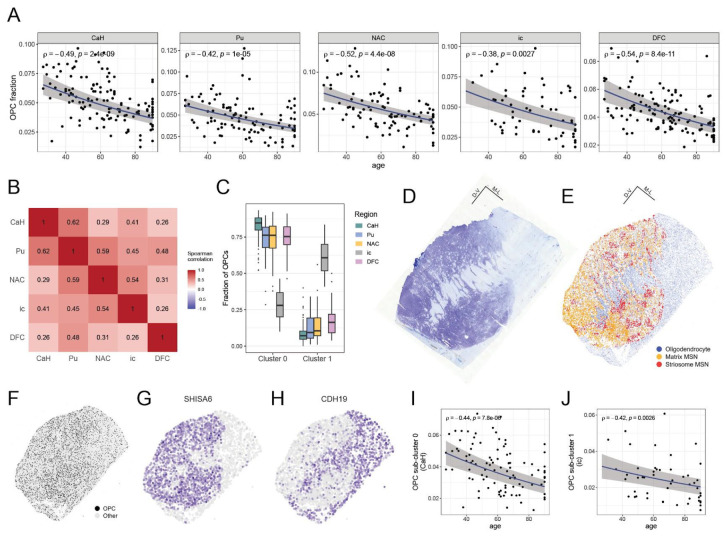
Decline in OPC populations in multiple brain regions with advancing age. (Extended analyses are in Figure S9). **A.** Attrition of OPCs with advancing age in five brain regions. In each sample, the abundance of OPCs was measured as a fraction of all nuclei sampled. Each point corresponds to a donor (n=131 in CaH; n=104 in Pu; n=97 in NAC; n=59 in ic; n=124 in DFC). Spearman rank correlation and nominal p-values are shown. Each blue line represents a beta-binomial fit of OPC abundance to age, with 95% confidence intervals shown by the gray ribbon. Modeling with a beta-binomial regression confirmed a significant decline of OPC fraction with age (β=−0.096 per decade of age; 95% CI: −0.12 – −0.07; Benjamini-Hochberg (BH) adjusted p-value=4.70e-12; n=44 tests; Table S4). **B.** Heatmap shows the cross-region correlation of “OPC fraction residual” – the observed OPC fraction minus the age-expected OPC fraction, where the age-expected fraction was calculated from the beta-binomial fit. Positive correlations are in shades of red, and negative correlations in blue. **C.** Representation (in each brain region) of cluster 0 and cluster 1 OPCs, calculated as a fraction of all OPCs. **D**. Nissl stain of the striatum (12 μm section) from one donor. **E.** Spatial transcriptomics data of an adjacent section from the same donor, generated with Slide-tags. Each point shown is an individual nucleus corresponding to either oligodendrocytes (blue), matrix MSNs (orange), or striosome MSNs (red). **F.** Spatial arrangement of OPCs (black) vs. all other cells (gray). **G.** Expression of *SHISA6* (a cluster 0 marker gene) in OPCs highlights gray matter compartments. Color of the points represents log-normalized transcript counts (darker purple = greater expression; lighter gray = lower expression). **H.** Expression of *CDH19* (a cluster 1 marker gene) in OPCs highlights white matter compartments. **I.** OPC cluster 0 abundance, as a fraction of all nuclei sampled (y-axis), declines with age (x-axis) in CaH (Spearman correlation coefficient and nominal p-value shown). Modeling with a beta-binomial regression confirmed a significant decline with age (β = −0.095 per decade; 95% CI: −0.122 to −0.067; BH-adjusted p-value = 7.02e-10; n=44 tests; Table S4). **J.** OPC cluster 1 abundance declines with age in ic. Modeling with a beta-binomial regression confirmed a significant decline with age (β = −0.072 per decade; 95% CI: −0.109 to −0.036; BH-adjusted p-value = 0.004; n=44 tests; Table S4).

We also looked for evidence of inter-individual variation in neuron abundances that was shared across striatal regions. Across individuals, the ratio of D1 to D2 MSNs was not significantly correlated between the caudate, putamen, and nucleus accumbens (Figure 3F, S6B). However, the abundances of interneuron subtypes exhibited strong evidence of inter-individual variation, in patterns that appeared consistently across the striatum. PTHLH-PVALB and TAC3-PLPP4 interneurons showed strong correlations in abundance across all pairs of striatal regions, particularly between caudate and putamen (Figure 2G,H, S7A-C). Abundances of different types of interneurons were also correlated with each other: donors with higher abundances of PTHLH-PVALB interneurons also had higher abundances of TAC3-PLPP4 interneurons (Figure S7D). We note that the observed correlations likely underestimate the strength of the underlying relationships, since striatal interneurons are sparse and our estimates of their abundance are therefore affected by substantial statistical sampling noise.

To evaluate the extent to which inter-individual variation in neuron abundances might reflect effects of age or sex, we applied a regression framework that incorporated age, sex, brain region, and additional donor- and sample-level covariates, while controlling for variability in nuclei counts per sample (see **Methods**). Canonical MSN subtypes, as a fraction of all MSNs, showed no significant associations with either age or sex (Table S4). Among interneurons (relative to all neurons), the TAC3-PLPP4 type had a statistically significant association with age (β = −0.047 per decade of age, 95% CI: −0.069 – −0.025, FDR-adjusted p-value = 0.001, Figure 2I, S7E). No type had a significant association with sex (Table S4). TAC3-PLPP4 interneurons appeared to decline modestly, by approximately 20% between ages 30 and 80, and at a similar rate across all three striatal regions (see **Methods**).

These results suggest that neuronal composition in the striatum reflects both consistent anatomical specialization between regions and inter-individual variability, the latter particularly evident for interneurons.

### OPC proportions decline with age across brain regions

We next asked whether any other cell type abundances were shaped by age or sex. Among well-ascertained cell types (median > 1% of all nuclei across samples), and after correcting for multiple hypothesis testing, only OPC abundance was significantly associated with age (β=−0.096 per decade of age, 95% CI: −0.12 – −0.07, FDR-adjusted p-value=4.70e-12, Table S4). Neither astrocytes nor microglia showed a statistically significant association of abundance with age (Figure S8A,B). No cell type showed a significant association with sex (Table S4).

OPCs (also referred to as polydendrocytes, in recognition of their many functions beyond serving as progenitors) remain proliferative in the adult brain and serve as the primary progenitors of myelinating oligodendrocytes; beyond this role, OPCs are increasingly recognized to have additional functions in the adult CNS, including through the synaptic inputs that they receive from neurons^53^.

The presence of OPCs (relative to all nuclei sampled) declined with advancing age in all brain regions analyzed (Figure 3A). This attrition was remarkably similar in magnitude across these brain regions, in all of which OPCs declined by about 40% between age 30 and age 80 (see **Methods**). A declining fraction of OPCs with age has been previously observed in the human DFC^54^ and in the mouse frontal cortex and striatum^55^.

To see whether the presence of OPCs is affected by individually varying factors beyond chronological age, we calculated an OPC fraction residual, the difference between a sample's actual OPC fraction and the OPC fraction predicted from their age (and other model covariates, see **Methods**). These OPC fraction residuals were positively correlated between all pairs of brain regions (Figure 3B, S8C): donors with higher-than-expected OPC abundance for their age in one brain region tended to exhibit similarly elevated OPC abundance in other regions. The consistency across diverse brain regions of both the effect of age and the residual unexplained by age suggests a substantial role for genetics and/or environment.

To better characterize OPC attrition and OPC diversity across these brain regions, we sub-clustered the OPC gene expression profiles (from ~155K nuclei), identifying seven transcriptionally distinct clusters (Figure S9, see **Methods**). The two most abundant clusters closely resembled OPC populations previously described as “immature” (cluster 0, expressing *SHISA6, SGCZ, GPC6*) and “mature” (cluster 1, expressing *CDH19, SEMA3E, GRIA4*)^56^. Among genes differentially expressed between these OPC populations, the most distinctive and interpretable signal for cluster 0 was the elevated expression of several genes that encode synaptic proteins (*GABRB2, KCNB2, SYN3, NCALD, SHISA6*) and specific transcription factors (*TCF7L1, PBX3, RFX4*). Cluster 1 was marked by higher levels of *CDH19*, which encodes a classic oligodendrocyte adhesion molecule. OPC clusters 0 and 1 were also distinguished by their expression levels of distinct sets of axon guidance genes (*CNTN5, EPHA5, ADGRV1* in cluster 0; *SEMA3E, PDZRN3, DACH2* in cluster 1) and extracellular matrix genes (*COL4A2, FBLN2, COL19A1* in cluster 0; *ADAMTSL1, COL20A1, CHRDL1* in cluster 1). These tissue-context-dependent expression profiles support the idea that OPCs have functions beyond serving as oligodendrocyte progenitors^57^.

Both of these clusters were observed in all brain regions; however, the internal capsule (the only white matter region analyzed) had a far lower proportion of cluster 0 OPCs and greater proportion of cluster 1 OPCs (3.6 - 7.1 fold; DFC and CaH, respectively) (Figure 3C, S9C,D). To ask whether these clusters might vary in their spatial distributions (e.g., potentially between gray and white matter regions) we examined Slide-tags spatial transcriptomics data of the striatum generated concurrently^46^ (Figure 3D,E). OPCs were evenly distributed throughout both the gray and white matter of the striatum (Figure 3F), consistent with reports in mice^55^. However, when visualized spatially, marker genes for cluster 0 highlighted gray matter compartments (Figure 3G, S9E), while marker genes for cluster 1 highlighted white matter compartments (Figure 3H, S9F). The spatial localization of these OPC populations further supports the idea that the fundamental difference between these types of OPCs may be tissue context (gray matter vs. white matter).

Across all brain regions, the quantitative presence of both OPCs with a cluster 0 profile (gray matter-enriched) and OPCs with a cluster 1 profile (white matter-enriched) declined with age (Figure 3I-J, S9G-H), further suggesting that this age-associated attrition affects OPCs broadly and in diverse tissue contexts.

### Age-associated changes in cell-type-specific RNA expression

Cells of any one type also vary from person to person in quantitative levels of the expression of each gene. To recognize such variation and the effects of age and sex upon it, we used a regression framework that incorporated donor-, sample-, and cell-level covariates (see **Methods**).

A key question in this and other brain research involves what set of donors are most informative for characterizing normal variation. A longstanding practice involves narrow inclusion criteria that exclude a large set of common health circumstances; this practice tends to focus research utilization on a small fraction of brain-bank samples, and excludes the vast majority of donations from younger people, whose manner of death more frequently involves drug overdose or suicide. Our project provided an opportunity to relax assumptions and empirically evaluate the effects of exclusion criteria on statistical power for various kinds of analyses. We considered the effect of two kinds of exclusions: "metadata-driven" exclusions based on reported health circumstance (including major depressive disorder and substance dependence; see **Methods**), and "snRNA-seq-data-driven" exclusions for donors identified as clear outliers in gene expression or cell-type proportions (see **Methods**). For analyzing effects of age, sex and common genetic variants, we found that snRNA-seq-data-driven exclusions tended to preserve or increase statistical power without altering estimated effect sizes (Figure S10, S15). In contrast, metadata-driven exclusions (based on available health information) substantially reduced statistical power in these analyses, mainly by reducing sample size without any compensating benefit in reduced variance (Figure S10, S15), resulting in a reduced number of discoveries at any given false discovery rate. Therefore, our subsequent differential expression analyses (and the genetic analyses described in a subsequent section) implemented only snRNA-seq-data-driven donor exclusions – generally involving about 5% of the available samples (which we believe generally arise from peri-mortem circumstances and perhaps local subclinical conditions in specific brain regions).

**Figure 4.**
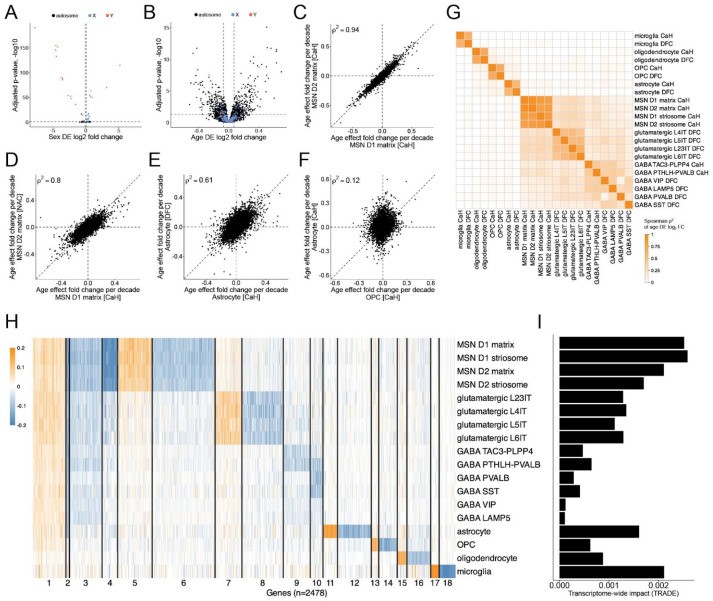
Age-associated changes in gene expression across brain cell types. (Extended analyses are in Figure S11). **A.** Analysis of sex differences in astrocyte gene expression (volcano plot). Each point represents one gene. Genes on the X chromosome, Y chromosome, and autosomes are shown in blue, red and black, respectively. **B.** Analysis of age-associated gene-expression changes in astrocytes (volcano plot). Effect sizes (x-axis) represent log2 fold change per decade of age (see **Methods**). Chromosomal locations of genes are indicated as in panel **A. C.** Comparison of age-associated gene-expression changes between D1 MSNs (x-axis) and D2 MSNs (y-axis) (matrix subtype, sampled in the caudate; Spearman’s ρ). **D.** Comparison of age-associated gene-expression changes between D1 MSNs (matrix subtype) sampled in the caudate (dorsal striatum) or the nucleus accumbens (ventral striatum). **E.** Comparison of age-associated gene-expression changes between striatal astrocytes (x-axis) and cortical astrocytes (y-axis). **F.** Comparison of age-associated gene-expression changes between OPCs (x-axis) and astrocytes (y-axis) in the caudate **G.** Heatmap shows correlation of age-associated gene-expression changes for each pair of cell types. Colors show Spearman’s ρ^2^ for correlations of gene-level log_2_-fold-change per decade of age. An extended version of this analysis is in Figure S11A. **H.** Clustering of genes by their patterns of age-associated expression changes in the various cell types. Columns show genes (n = 2478) grouped by k-means clustering of their age-associated expression changes (log2-fold-change per decade of age, shown in shades of orange and blue) across the various cell types. An extended version of this analysis is in Figure S11D. **I.** Magnitude of transcriptome-wide age-associated expression changes in each cell type, as estimated from these data using TRADE ^61^.

Across the striatal and cortical regions we analyzed, associations of gene expression to chromosomal sex were largely limited to sex chromosome genes – primarily Y chromosome genes and *XIST* and *TSIX* on the X chromosome (Figure 4A). This pattern was shared across all cell types in the analysis.

In contrast to the very small effect of sex, age appeared to affect the expression of thousands of genes, on all chromosomes (Figure 4B) and in every cell type. Age-associated effects were highly correlated between closely related cell types (e.g., subtypes of MSNs) (Figure 4C) and between homologous cell types across regions (e.g., MSNs in different striatal regions; striatal versus cortical astrocytes) (Figure 4D,E, S11B,C), though the changes in MSNs appeared to proceed a bit more slowly in the nucleus accumbens than in the caudate (Figure 4D).

In contrast to the consistent effects of age on the same or similar cell types in different brain regions, age affected gene expression in different cell types (even in the same brain region) in very different ways (Figure 4F). Even when focusing on neurons, age affected gene expression in different types of neurons in quite different ways and to quite different degrees (Figure 4G,H,I).

To better recognize the underlying patterns that shape global and cell-type-specific components of brain aging, we allowed the data to organize genes into clusters (using k-means clustering) based on their patterns of cell-type-specific age-associated expression changes (Figure 4H, S11D). A small subset of the age-affected genes (cluster 1 in Figure 4H) tended to increase modestly in expression with age across cell types. These included genes related to stress and glucocorticoid signaling (e.g., *FKBP5;* Figure S12), inflammatory pathways (*RSAD2, MGST2, CKLF, TNFSF13B, CD247*), DNA damage responses (*RAD51B, RPA1, BRIP1, FANCL, USP45*), and metabolism (*SLC25A24, ALDH3A2, PNPLA7, PTGR1/2, LSS*). Many of these kinds of changes have previously been recognized in analyses of age-associated gene-expression changes in "bulk" brain tissues, which have often aligned around functional themes such as metabolism, stress, inflammation, and DNA repair^58-60^.

Beyond this modest set of shared, pan-cell-type changes in gene expression, each type of cell exhibited a substantial set of cell-type-specific age-associated changes in RNA expression. For glial cell types, these changes replicated strongly between analyses of the striatal regions and analyses of the DFC (Figure 4G, S11A). Among neurons, changes were strongly shared only between certain specific (and closely related) neuronal types: canonical MSN sub-types (D1 and D2; matrix and striosome) and intratelencephalic-projecting (IT) glutamatergic neurons of different cortical layers. MSNs, interneurons, and cortical pyramidal neurons exhibited only slightly positive correlations (with one another) in their age-associated transcriptional changes (Figure 4G, S11A), with these changes arising from a small subset of the age-affected genes (Figure 4H). There was almost no correlation in the age-associated changes of the various glial cell types when compared with one another or with neurons (Figure 4G,H).

In principle, age might affect the biology of some brain cell types more than others. Quantifying and comparing the magnitude of transcriptional changes across cell types has been challenging due to the difference in power to recognize age-associated changes in different cell types, as very different numbers of cells and RNA transcripts (unique molecular identifiers, or UMIs) are sampled for each cell type, making the number of differentially expressed genes (whose identification is highly sensitive to statistical power) a misleading estimate of impact. To overcome this, we used transcriptome-wide analysis of differential expression (TRADE^61^), a statistical framework for recognizing the distribution of true differential expression effects while accounting for estimation error, to estimate the average transcriptome-wide impact of age upon each cell type (Figure 4I).

MSNs exhibited larger age-associated transcriptional changes than any other striatal or cortical cell type did (Figure 4H,I). More generally, projection neurons, including MSNs and cortical glutamatergic neurons, exhibited much larger age-associated changes than interneurons did (Figure 4H,I). Among glial cells, microglia and astrocytes exhibited the largest age-associated changes in gene expression, followed by oligodendrocytes and then OPCs (Figure 4I). Curiously, of all cell types in the analysis, OPCs exhibited the smallest age-associated effects on RNA expression (Figure 4I), despite being the only glial cell type that exhibited substantial attrition with age (Figure 3A). Similarly, GABAergic TAC3-PLPP4 interneurons – whose abundance was also impacted by age (Figure 2I, S7E)– showed a relatively modest transcriptional impact of age, compared to projection neurons (Figure 4I).

To better characterize these age-associated gene expression changes in each cell type, we ranked genes by the age-associated differential expression test statistic and performed Gene Set Enrichment Analysis (GSEA) on these rankings^62^ (Table S5). MSNs, interneurons, and astrocytes all showed age-associated declines in expression of gene encoding cellular respiration enzymes (e.g., GO:0045333; p-value = 1e-10), a set of genes long observed to decline with expression with age in "bulk" tissue studies. However, this pattern was by no means universal to all cell types: microglia, for example, exhibited the opposite relationship, increasing expression of this same set of genes with age (e.g., GO:0045333; p-value = 4e-10). This suggests that these metabolic changes may themselves result from changes in cell-type-specific activities.

**Figure 5.**
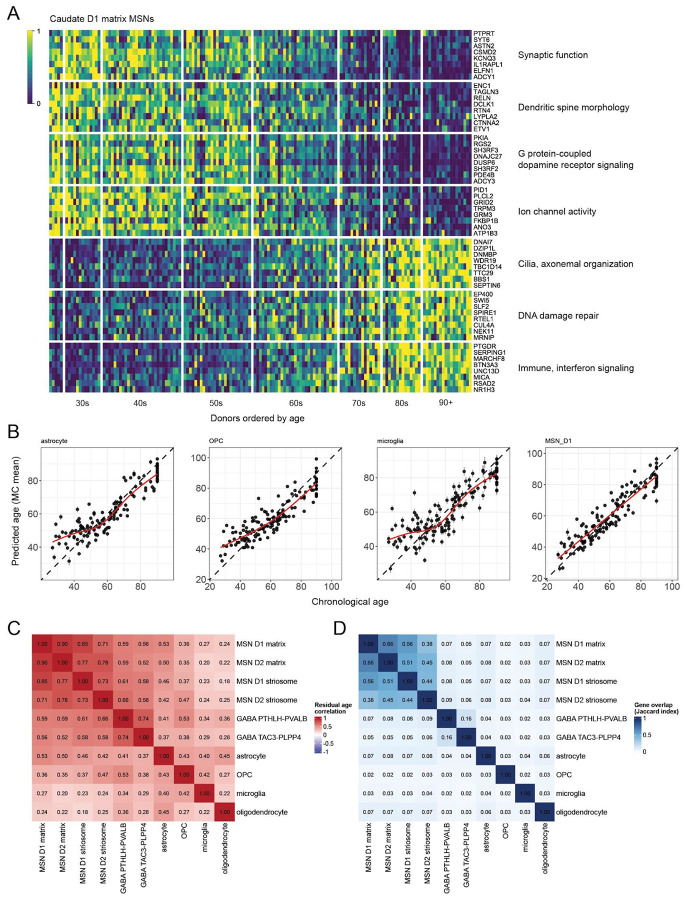
Chronological age can be predicted from gene expression in various cell types. (Extended analyses are in Figures S13 and S14.) **A.** Age-associated changes in D1 matrix MSN gene expression, within the caudate. The 150 brain donors (columns) are ordered along the x-axis by ascending age (27 to 90+). Expression values (transcripts-per-million) for each gene (row) have been rescaled such that 0 (blue) represents the 10th percentile among all donors and 1 (yellow) represents the 90th percentile. **B.** RNA-expression-based age prediction in four representative caudate cell types. For each cell type, donor chronological age (x-axis) is plotted against RNA-expression-predicted age(y-axis), with predictions derived from an elastic net model. Each point represents one donor. The dashed line denotes the identity line (predicted = chronological age). Vertical error bars indicate plus or minus 1 SD around the predicted age. The red curve shows a Gaussian additive model (GAM) fit of predicted age as a function of chronological age. Age residuals (expression-predicted minus chronological age) are defined relative to this fitted relationship. **C**. Positive correlation of cell-type-specific RNA-expression "clocks", beyond the shared effects of chronological age. The heatmap shows pairwise correlations (for each pair of caudate cell types) of the GAM-corrected age residuals. **D**. Minimal overlap of the genes used to predict age in most cell types. The heatmap shows Jaccard indices quantifying overlap among age-associated genes used in each cell-type-specific model.

In neurons, this apparent average decline in cellular respiration activities was accompanied by a decline in expression of ribosomal/translation programs (e.g., GO:0003735; FDR = 8e-29), perhaps suggesting reduced commitment to (energetically expensive) turnover activities. In MSNs, the most prominent age-downregulated modules comprised terms related to synaptic function and signaling (e.g., GO:0097060; FDR = 2e-28), including reduced expression of *GPR158, RGS9, ADCY3, PDE10A, PKIA*, and *PPP1R1B* (additional genes represented in Figure 5A). GABAergic *PTHLH-PVALB* interneurons also showed a decline in expression of genes with synaptic functions (e.g., GO:0097060; FDR = 1e-28), with a pronounced (4:1), overall bias towards downregulation of these genes with age. Together, these neuronal signatures indicate a coordinated decline in bioenergetic activity and translation, in conjunction with reduced synthesis or turnover of synaptic molecular machinery.

GSEA analysis revealed the diverse cell-type-specific activities that appeared to be age-associated in the various glial cell types. In astrocytes, age was associated with increasing expression genes with annotated roles in immune-interface programs (e.g., GO:0002449; FDR = 9e-05) and complement activation (e.g., GO:0006958, FDR = 0.01) (including elevated expression of the complement gene *C3* with age). Microglia showed increased expression of genes with immune activation signatures (e.g., GO:0042611; FDR = 0.005) alongside reduced expression of TGF-β response pathways (e.g., GO:0071559; FDR = 1e-4). Oligodendrocytes exhibited reduced expression of genes associated with cell projection and cytoskeleton-associated components (e.g., GO:0000904; FDR = 7e-11), alongside increased expression of genes with roles in antigen processing and presentation (e.g., GO:0003823; FDR = 1e-05). Finally, the strongest signal in OPCs involved reduced expression of genes with roles in synapse organization and cell-cell communication (e.g., GO:0097060; FDR = 9e-15), consistent with loss, weakening, or reduced turnover of neuron-OPC synaptic contacts.

These findings point to a broad set of age-associated molecular changes in the striatum, largely cell-type-specific, and affecting MSNs in particularly strong ways.

### Inter-individual variation in biological aging

Differential expression analyses identify genes whose expression tends to increase or decrease with advancing age. An interesting question involves the kinetics of these changes in individual persons, who could in principle exhibit faster-than-average or slower-than-average aging – either within specific cell types or across all cell types. Inspired by the informativeness of methylation-based aging clocks in analyzing blood samples^63^, we sought to ask whether cell-type-specific RNA-expression measurements could be similarly informative about age-associated processes in brain cell types. To do this, we constructed an age prediction model for each cell type using a penalized regression model (elastic net^64^, see **Methods**). This model generated, for each donor and cell type, both a predicted age and an age residual (defined as the difference between predicted and chronological age).

We trained separate models for each cell type (by region) and evaluated their ability to estimate each donor's age from RNA-expression data alone. These models were trained using the same cell-type–specific age-associated genes identified in the previous section. Across models, RNA-expression-predicted age was highly correlated with actual chronological age (Figure 5C). The median of the model-level mean absolute error was 6.6 years (range 5.1–14.4 years), with a corresponding median absolute deviation of 5.6 years (Figure S13A). Model accuracy depended strongly on the number of age-associated differentially expressed genes (adjusted R^2^ ≈ 0.72) and was thus greater for MSNs (Figure S13B). Predictions at younger and older ages were shifted toward the cohort mean (Figure 5C). As with overall prediction error, the magnitude of this shift tracked the number of age-associated differentially expressed genes available to the model (Figure S13C). Uncorrected residual age showed a shared age-dependent bias across models (Figure S14A). To account for this bias, we defined residual age relative to the fitted relationship between predicted and chronological age, which eliminated this age dependence and removed the correlation driven by this shared bias (Figure S14B).

For each cell type, the difference (residual) between chronological age and RNA-expression-predicted age could represent simple prediction error, or a difference between biological aging (which may vary across individuals and cell types) and simple chronological age. In support of the latter interpretation, we found that these residuals were positively correlated between all pairs of cell types in the analyses (Figure 5D), despite the fact that the genes used to estimate biological age in different cell types were almost completely distinct (Jaccard index < 0.1 for most cell-type pairs) (Figure 5E). This suggests that these cell-type-specific "aging clocks" exhibit a tendency to run faster or slower together in an individual person, a tendency that could in principle result from shared influences such as circulating factors, life history, or genetics. This correlation was only partial, though, and exhibited fluctuations that appeared to be biologically meaningful: for example, the correlations-of-residuals were generally larger when comparing the same cell type across brain regions than when comparing different cell types within a brain region (Figure S14C,D).

### Effects of common genetic variation on gene expression

A pervasive source of biological diversity is the genetic variation that percolates through human populations. Any two people will generally have tens of millions of genetic differences. Most phenotypes are genetically complex – shaped by common and rare genetic variation at many loci, and thus requiring large samples for genetic mapping. However, samples of the current size (100 - 200 donors) are often sufficient for identifying strong effects of common genetic variants and haplotypes on expression levels of nearby genes (expression quantitative trait loci, eQTLs). To map common genetic effects on gene expression in each of the cell types of the striatum and cortex, we jointly analyzed the snRNA-seq and whole-genome sequence data from the brain donors.

**Figure 6:**
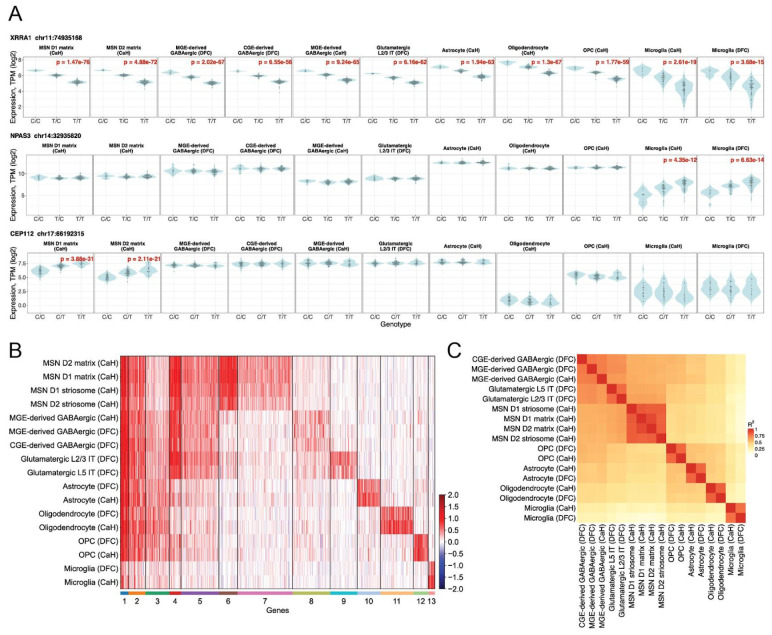
Effects of common genetic variation on gene expression. (Extended analyses including additional cell populations and brain regions are in Figures S16 and S17.) **A.** Log_2_-transformed expression levels of three genes (of 9,899) whose expression levels associated with common genetic variants: *XRRA1* (rs10899052), *NPAS3* (rs61980314), and *CEP112* (rs7209037). Brain donors' expression levels are shown for each genotype (as violin plots), for a variety of striatal and cortical cell types (red text indicates Benjamini–Hochberg adjusted p values). **B.** K-means clustering (K=13) of the 9,899 eQTLs based on their patterns of effect sizes on gene expression across the cell types in the analysis. Effect sizes represent standardized change in gene expression (inverse-normalized log2-transformed values). For each effect, shades of red indicate the most common effect direction and shades of blue indicate any cell types with an opposite direction of association. **C.** Pairwise correlations (among cell types) of genome-wide sets of eQTL effects (corresponding to the rows in panel B). For each cell-type pair, Spearman’s ρ^2^ was calculated using eQTLs significant in at least one of the two cell types. Rows and columns are hierarchically clustered. The analyses in panels B and C are extended to additional brain regions in Figure S16.

We identified 9,899 genes with significant evidence (at a false discovery rate of 0.01) of cis-regulation ("eGenes") by a nearby SNP or haplotype in one or more cell types (three illustrative examples are shown in Figure 6A). As expected, more deeply-sampled cell populations such as D1 matrix MSNs yielded more eQTL discoveries (n = 4,530 unique eGenes detected in DFC and CaH) than sparser cell types such as microglia did (n = 408) (Table S6).

To systematically characterize patterns of cell-type specificity among these 9,899 eQTLs, we used k-means clustering to group eQTLs by shared patterns of "effect sizes" – the fractional change (in each cell type) in gene expression per allele inherited – across the full set of cortical and striatal cell types (Figure 6B, see **Methods**).

Some 16% of the eQTL effects we ascertained (n = 1,540 of 9,899; gene clusters 1 - 3 in Figure 6B) were shared across most of the cell types in the analysis, though often excluding microglia, which arise from a different developmental lineage^65^. Such effects varied in magnitude across the cell types but almost never varied in direction (more versus less expression for a given allele). For example, expression of the *XRRA1* gene associated strongly with the same common haplotype (tagged by the SNP rs10899052) in every cell type of the striatum and cortex (Figure 6A, first row).

Another 16% of the eQTLs (n = 1,559 of 9,899; clusters 4 and 5 in Figure 6B) were shared across many neuronal classes (including projection neurons and interneurons), often with more-modest effects in one or more of the neuroglial cell types.

A large majority of eQTL effects, though, were specific to a cell type, almost always manifesting in that cell type in all of the brain regions in which that cell type was present (Figure 6B, S16). These included clusters of eQTLs specific to MSNs (clusters 6 and 7), interneurons (cluster 8), cortical pyramidal neurons (cluster 9), astrocytes (cluster 10), oligodendrocytes (cluster 11), OPCs (cluster 12), and microglia (cluster 13) (Figure 6B). Specific examples included a common genetic effect on *NPAS3* expression in microglia (Figure 6A, second row) and a common genetic effect on *CEP112* expression in MSNs of all types (D1 and D2; striosome and matrix) (Figure 6A, third row).

Notably, *NPAS3* illustrated a broader pattern in which cell-type-specific eQTLs often appeared in cell types that did not express the gene most strongly: microglia had the lowest *NPAS3* expression of all cell types in the analysis, yet were the only cells in which this genetic effect clearly manifested; and the genetic effect at *CEP112* tended to reduce expression in MSNs below the expression levels in other neurons and astrocytes (Figure 6A). In seven of eight cell-type-specific clusters (clusters 6 - 12), only 24 - 42% of eGenes had their highest median expression in the cell type in which they manifested cell-type-specific regulatory effects; for the microglia-specific cluster (cluster 13), this fraction was 62% (Table S7).

Overall, some 68% of the eQTL effects (n = 6,800 of 9,899; clusters 6 - 13) exhibited one of these forms of cell-type specificity – a number that is likely a lower bound on the true cell type specificity of brain eQTLs, since a cell-type-specific eQTL has fewer chances to be discovered at statistical significance.

These data also afforded the opportunity to compare cell-type specific eQTLs across striatum and cortex. Glial cell eQTLs were almost identical between cortex and striatum: nearly all genetic effects that manifested in striatal astrocytes were also apparent in independent analyses of cortical astrocytes, and vice versa; the same was also true for OPCs, oligodendrocytes, and microglia (Figure 6B,C). The various types of MSNs (D1 and D2 MSNs; and matrix and striosome MSNs) also tended to exhibit highly similar effects of common genetic variants (Figure 6B,C), as did MSNs from different striatal regions (Figure S16).

Neurons with more-distinct molecular identities, however – such as MSNs, interneurons, and cortical pyramidal neurons – tended to manifest highly divergent sets of genetic effects (Figure 6B,C). This may foreshadow the existence of a far-larger, heterogeneous set of common genetic effects that remain to be discovered in other neuronal types beyond the striatum and neocortex, especially among the astonishing variety of neuronal types that are found beyond the telencephalon^66,67^.

Among the eQTLs, genes that are intolerant to loss-of-function mutations^68^ tended to have smaller genetic effect sizes (fold-changes) than less-constrained genes did (Figure S17). This was true for the eQTLs as a whole (p = 9.3e-19), and for each cell type separately, and for each eQTL cluster (1 through 13) defined by patterns of cell-type specificity (Figure S17). This aligns with a recent finding about human blood eQTLs^69,70^ and suggests that eQTLs in the most functionally critical genes tend to involve more-modest quantitative effects – and thus require larger cohorts to recognize at genome-wide significance. This relationship may foreshadow that, as sample sizes for such analyses continue to expand, the number of findings that involve constrained and disease-affecting genes will grow disproportionately.

## Discussion

Decades of work by brain banks, and the generosity of brain donors and their families, is increasingly meeting new genomic analysis technologies that unlock the cellular and molecular information in these tissues. Human brain tissues offer unparalleled opportunities to investigate pathogenic processes, especially for the many human brain disorders that lack mechanism-based animal models.

At the same time, such work lacks the factors that have been the cornerstone of most laboratory studies and biological training: controlled laboratory conditions studying a single experimental variable in isolation. Human tissue studies operate in a context in which thousands of factors vary simultaneously – often without prior knowledge about the magnitude or independence of this variability. Better appreciating the patterns that underlie and structure human biological variation can support the substantial intellectual and resource investment involved in the design and interpretation of human brain studies, from selecting donors to appropriately calibrating statistical analyses and claims. We hope that the data generated by this project provide a useful resource for such studies.

One of the most remarkable observations from these data involved the degree to which cell type proportions vary from person to person (Figure 2). The quantitative representations of astrocytes, microglia, OPCs, and striatal interneurons — which might have been reasonably assumed to be optimized parameters of the human brain — were in fact highly variable (Figure 3F,G,H). This manifested in our data as consistent properties of individuals that re-appeared in all of the brain regions we analyzed from the same person (Figure 2B,C,F-H) – to an extent that was only partially (and for only certain cell types) explained by age effects (Figure 3A,B). This observation raises intriguing questions: what is the functional consequence of having a larger-than-average number of interneurons, OPCs, astrocytes, or microglia? How might such variation influence the biology of neural systems, the flexibility of cognitive processes, or the properties of neuronal circuits?

A fascinating question for much future work involves the causes and biological mechanisms that underlie this variation. For OPCs, age contributed to differences in abundance (in both gray- and white-matter tissue contexts, across striatum and cortex), but age alone explained only part of the interindividual variability that manifested across all of an individual's brain regions (Figure 3B). Sex had no apparent impact, despite ample statistical power to observe such relationships. Brain developmental processes seem likely to contribute, for example via heterochronic shifts in the timing of neurogenesis or gliogenesis, differences in migratory patterns, or variation in apoptotic pruning during development. Genetic factors seem likely to contribute as well, and as cohort sizes increase it will become possible to map such effects to specific genes.

Each cell type exhibited pronounced differences in gene expression across donors. Age, but not sex, had a strong relationship to gene expression in every cell type (Figure 4A,B) – and the vast majority of these effects were specific to each cell type (Figure 4G,H). We found that people's ages could be estimated with surprising accuracy (to about 5 years) based on only these RNA-expression patterns (Figure 5B) – but that, at the same time, there was clear evidence that these same changes progress more slowly or rapidly in some individuals than in others, and (within individuals) in some cell types than others. In human brain tissue studies, analytically addressing such effects could in principle help to make other genetic and biological effects more clear. We hope that our data help provide a resource for such analyses and enable many new analytical approaches to be conceived, trained and critically evaluated.

Age appears to affect some brain cell types much more than others (Figure 4H). Of all the striatal and cortical cell types in our analyses, MSNs showed the largest effects of aging on gene expression, followed by cortical glutamatergic neurons (also a type of projection neuron); the various types of interneurons in these brain regions manifested much smaller changes. It is interesting to speculate whether this sensitivity arises in projection neurons' need to support spatially distant metabolic and physiological processes, or from their remarkable number of dendritic arbors and spines. The recognition of aging effects in a more-diverse set of types of neurons across the brain may well reveal clear patterns.

One way in which these results challenged our expectations was that the cell types with the greatest vulnerability to age-associated loss – OPCs and TAC3-PLPP4 interneurons – were not the cell types with the largest age-associated gene-expression changes (Figure 4H); in fact, OPCs showed the most modest changes among the glial cell types, and TAC3-PLPP4 interneurons showed the most modest expression changes among the neuronal cell types. In the case of OPCs, it may be that renewal from a stem population attenuates the effects of aging, even as the capacity of that stem population itself declines with age. In the case of these interneurons, one possibility is that age-associated changes lead to loss of the more severely affected interneurons (reducing their numbers, but also reducing the average amount of age-associated change among the surviving interneurons) but do not do this in projection neurons.

The data provided an opportunity to recognize common genetic effects on gene expression levels (eQTLs) in a wide variety of neuronal and glial cell types in multiple brain regions (Figure 6). We found that genetics shapes inter-individual variation in gene expression in ways that are remarkably cell type specific: a large majority of eQTLs identified predominantly affected expression in a particular neuronal or glial cell type (Figure 6C). Another clear pattern was that among cell-type specific genetic effects, the cell type in which a variant alters gene expression was often not the cell type with the highest baseline expression of that gene; simply knowing which cell type expresses a gene most strongly seems to provide a poor compass to recognizing where genetic effects manifest. Comparing cell-type-specific effects across striatum and cortex, we found that each glial cell type manifested very similar sets of genetic effects in cortex as in striatum. In contrast, sets of eQTLs for different types of neurons were quite distinct. This predicts that a vast set of neuron-specific genetic effects in other brain regions are yet to be discovered — among which MSNs and cortical projection neurons represent only a small fraction of total neuronal diversity.

Finally, we would like to highlight several observations that we hope could benefit the design and interpretation of research that uses human brain tissue.

The first involves the inclusion and exclusion criteria that are used to define "cases" and "controls" in disease-focused analyses of human tissue. It is often assumed that the key to successful analysis is to minimize within-group heterogeneity insofar as can be accomplished from available medical records. This often leads to the adoption of narrow inclusion criteria for affected individuals (to reduce disease heterogeneity) and to broad exclusion criteria for controls (e.g. excluding common health conditions), which can greatly reduce sample size. Our results suggest that even criteria such as those defining our core “normative” donor set admit abundant biological variation (Figure 2,3). The hoped-for reduction in variance obtained by narrow inclusion criteria and broad exclusion criteria may often be small compared to the biological variation that is present within each group. In the context of abundant inter-individual biological variation, it will usually be more informative to expand the sizes of case and control groups when possible, and to favor analyzing a larger number of donors over simple technical replication or deeper sequencing of cells from the same donors.

The second involves utilizing experimental approaches that minimize technical variability, recognizing that biological variation is ubiquitous yet often mixed with technical and statistical-sampling effects on variables of interest. Here we utilized an approach for isolating nuclei in large pools ("villages") of about 20 donors handled as a single sample and therefore exposed to identical handling conditions. Key factors such as age and (in some study designs) sex or diagnosis are ideally balanced across pools as much as possible. Other alternative experimental approaches could in principle be developed in the future. Any such approach should be evaluated empirically; reasonable metrics could include reductions in the variance (across donors) of quantitative measurements, and increased statistical power for recognizing already-established biological effects (such as the thousands of effects of age on gene expression described here).

The third involves the selection of appropriate statistical tests. The vast majority of the measurements we made – whether of cell-type proportions, or gene expression levels – did not exhibit "normal" (Gaussian) distributions. For this reason, the conventional statistical tests that are used in most biological studies – including t-tests, Pearson correlations, and conventional linear regressions (all of which assume such distributions) – will routinely overestimate the statistical significance of the inevitable average differences between experimental groups. We encourage researchers to use the data from this project to assess the ways in which biological variables of interest are empirically distributed – and to design studies, interpret data and perform statistical tests with these distributions in mind. This will often mean using non-parametric statistical frameworks and permutation tests, which are far less vulnerable to inflating the apparent significance of statistically unremarkable differences between experimental groups.

Our hope is that these data and analyses, and much other such work to come, will make natural human biological variation an ever-more-useful tool to neurobiology – a source not of unwelcome analytical complexity, but of new questions, new discoveries, and a deeper understanding of human biology.

### Limitations of the study

Our analyses do not identify the biological mechanisms by which these sources of biological variation arise, nor (beyond the effects of common genetic variants on gene expression) make any particular claim about the relative contributions of genetics and environment. Such questions deserve extensive future work by a wide variety of scientific approaches. We hope our results inspire the conception of such studies and inform their design.

Humans have an enormous variety of subclinical biological conditions, as well as conditions that do not receive medical attention or a formal diagnosis. Our observations that certain kinds of biological variation appear frequently and in specific patterns make no claims about whether or not such biological variation is "healthy", affects brain functions, or contributes to vulnerabilities – in fact, we believe this is an important topic for future research.

## Supplementary Material

Supplement 1

Supplement 2
