# Supplementary material for: Inter-individual variation of cellular and gene-expression properties of the human striatum
Source: bioRxiv. 2026 Mar 23:2026.03.20.713160. Preprint. [Version 1] doi: 10.64898/2026.03.20.713160 (PMC13042060; doi:10.64898/2026.03.20.713160)
Supplement: Supplement 2 [file NIHPP2026.03.20.713160v1-supplement-2.pdf]

bioRxiv preprint doi: https://doi.org/10.64898/2026.03.20.713160; this version posted March 23, 2026. The copyright holder for this preprint (which was not certified by peer review) is the author/funder, who has granted bioRxiv a license to display the preprint in perpetuity. It is made available under aCC-BY 4.0 International license.

Supplemental figures

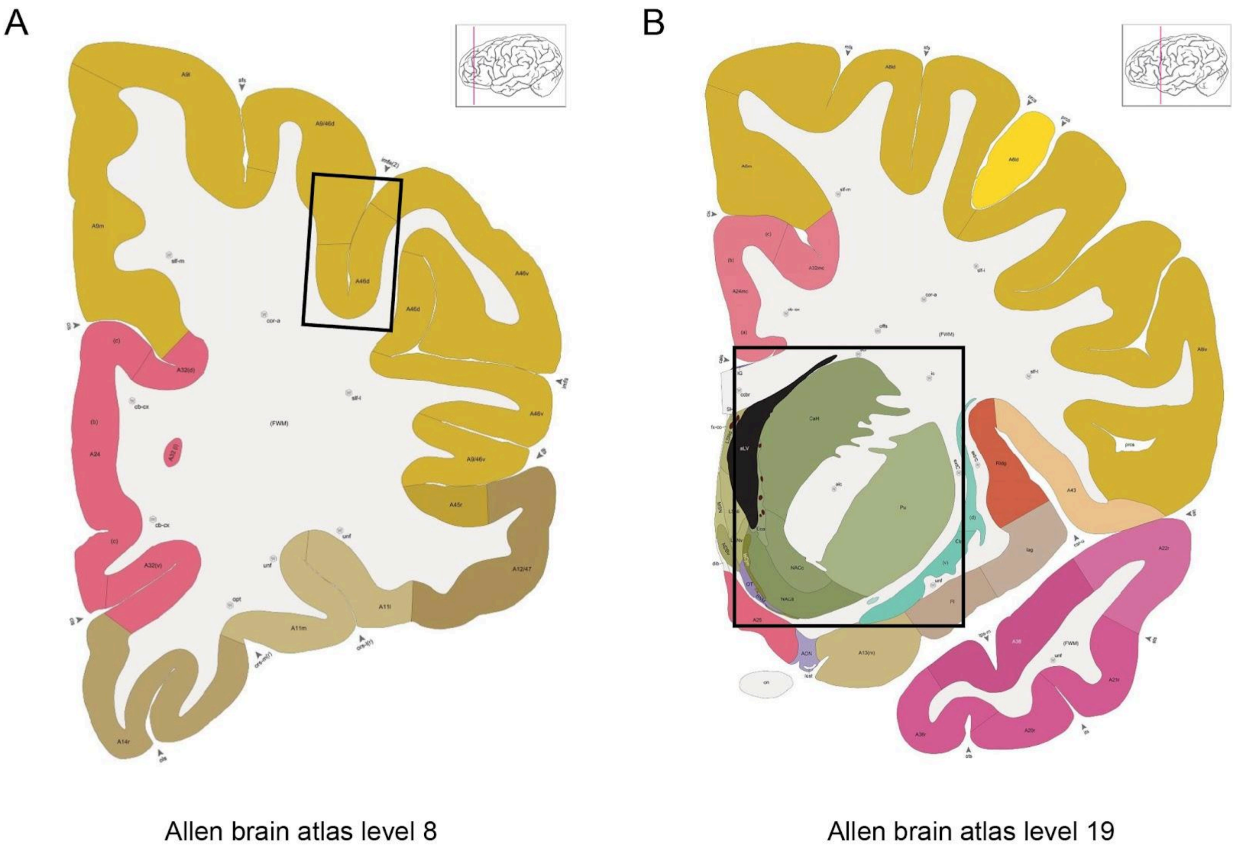

Figure S1. Tissue procurement and dissection reference for regions of interest (ROIs). Images from the Allen Institute adult human brain atlas, modified Brodmann annotation[34]. Black box represents the requested ROIs from NBB. **A**. Atlas level for dorsolateral prefrontal cortex (DFC) samples. **B**. Atlas level for the striatum complex (caudate, putamen, nucleus accumbens, and internal capsule) samples.

bioRxiv preprint doi: https://doi.org/10.64898/2026.03.20.713160; this version posted March 23, 2026. The copyright holder for this preprint (which was not certified by peer review) is the author/funder, who has granted bioRxiv a license to display the preprint in perpetuity. It is made available under aCC-BY 4.0 International license.

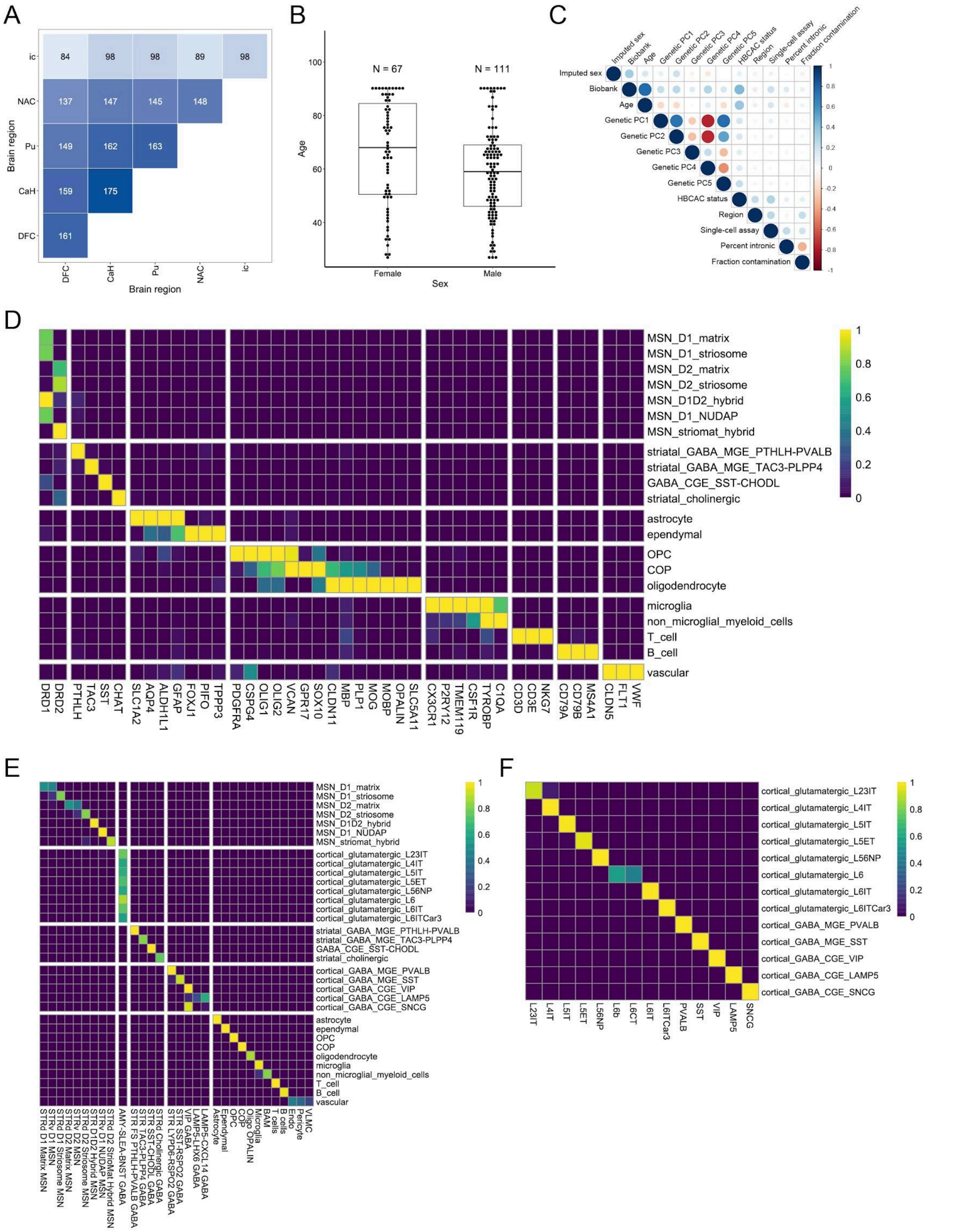

bioRxiv preprint doi: https://doi.org/10.64898/2026.03.20.713160; this version posted March 23, 2026. The copyright holder for this preprint (which was not certified by peer review) is the author/funder, who has granted bioRxiv a license to display the preprint in perpetuity. It is made available under aCC-BY 4.0 International license.

Figure S2. Overview of donor sampling, metadata structure, and cell type annotations.
A. Donor sampling across brain regions. For each pair of brain regions, the heatmap shows the number of donors for which both regions were sampled. Values along the diagonal are the total donor counts for each region, as shown in **Figure 1B**. **B.** Counts and age distribution of female and male donors. **C.** Heatmap summarizing pairwise associations among demographic, genetic, technical, and sequencing-quality variables. Numeric–numeric pairs show Pearson correlations; factor–factor pairs show Cramér's V; and mixed numeric–factor pairs show the proportion of variation in the numeric variable explained by group differences. Pearson correlations range from –1 to 1, whereas the other measures range from 0 to 1. Circle size and color indicate the magnitude and direction of association. A complete description of variables is provided in **Table S3**. **D.** Cell-type specificity of gene expression measurements. Heatmap shows expression measurements for some of the genes (columns) whose expression levels distinguish major cell classes (rows). Colors represent normalized expression by column (across major cell classes) such that the highest expression is 1 (bright yellow) and lowest is 0 (dark purple). **E.** Confusion matrix comparing cell identities as inferred from two analytical approaches: MapMyCells label transfer from the consensus basal ganglia taxonomy (columns), and manual unsupervised clustering annotations (rows). Values (colors) represent fractions (of the total) in each row. **F.** Confusion matrix comparing cell identities as inferred from scPred label transfer from the Allen Brain human cortical neuron taxonomy (columns), and manual unsupervised clustering annotations (rows). Values (colors) represent fractions of the total in each row.

bioRxiv preprint doi: https://doi.org/10.64898/2026.03.20.713160; this version posted March 23, 2026. The copyright holder for this preprint (which was not certified by peer review) is the author/funder, who has granted bioRxiv a license to display the preprint in perpetuity. It is made available under aCC-BY 4.0 International license.

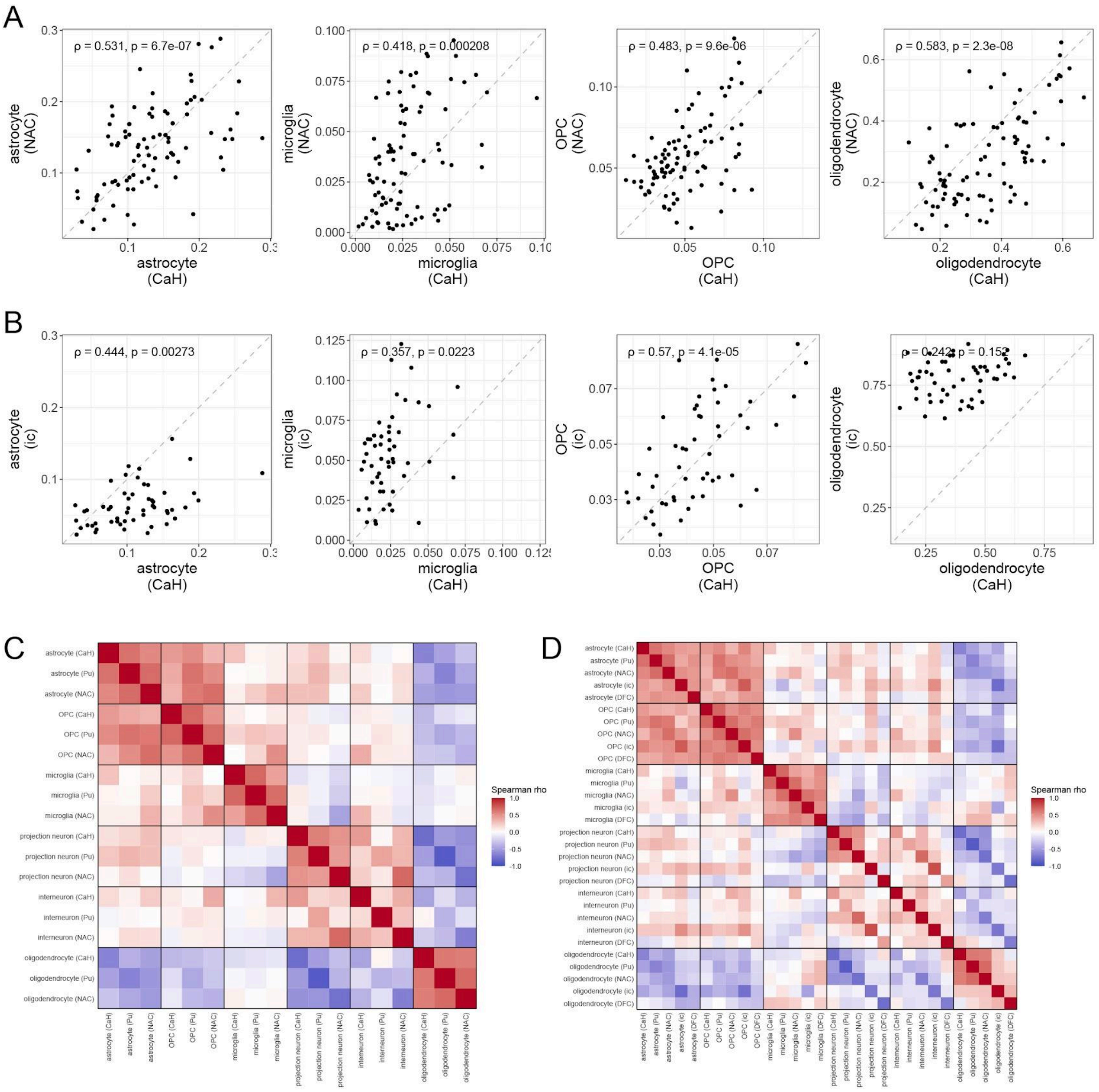

Figure S3. Cross-brain-region correlations in cell type abundances.
A. Representations of glial cell types (astrocytes, microglia, OPCs, oligodendrocytes) in the caudate (x-axis) and nucleus accumbens (y-axis) in the same 90 donors (points). Abundances are quantified as fractions of all nuclei ascertained in a donor. Spearman correlation coefficient and corrected p-value are shown for each cell type (Benjamini-Hochberg (BH) procedure, n=498 tests). **B.** Same as **A**, for the caudate (x-axis) and internal capsule (y-axis) across 54 donors. **C.** Pairwise correlations of cell type abundance measurements – quantified as fractions of all nuclei ascertained in a sample – in striatal regions (caudate, putamen, and nucleus accumbens). Spearman correlation coefficients are shown; positive correlations in red, negative in blue. Note the blocks of positive correlation (red) for specific cell types regardless of the brain regions in which they are measured. **D.** Same analysis as **C**, for all five brain regions surveyed.

bioRxiv preprint doi: https://doi.org/10.64898/2026.03.20.713160; this version posted March 23, 2026. The copyright holder for this preprint (which was not certified by peer review) is the author/funder, who has granted bioRxiv a license to display the preprint in perpetuity. It is made available under aCC-BY 4.0 International license.

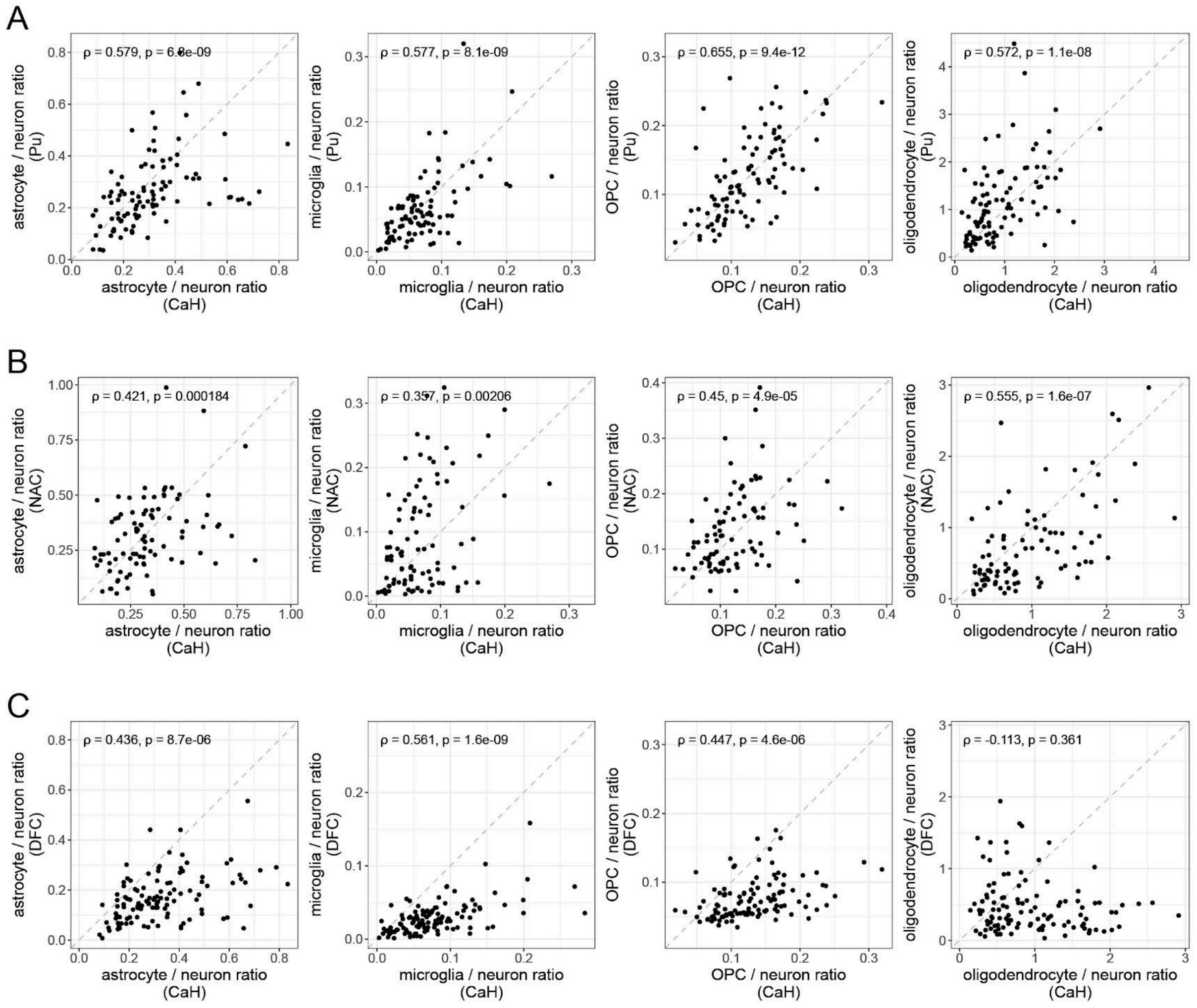

Figure S4. Cross-brain-region correlations in the abundances of glial cells.

A. Representations of glial cell types in the caudate (x-axis) and putamen (y-axis) across the same 98 donors. Here, abundances were quantified relative to the total number of neurons in the same sample, so that the glial cells do not influence one another's abundance estimates. Spearman correlation coefficient and corrected p-value are shown for each cell type (Benjamini-Hochberg (BH) procedure, n=498 tests). **B.** Same analysis as in **A**, focusing on correlation between caudate (x-axis) and nucleus accumbens (y-axis) across 90 donors. **C.** Same analysis as in **A**, comparing caudate (x-axis) and dorsolateral prefrontal cortex (y-axis) across 114 donors. Astrocyte, microglia, and OPC abundances are significantly correlated, indicating that these patterns extend beyond the striatum.

bioRxiv preprint doi: https://doi.org/10.64898/2026.03.20.713160; this version posted March 23, 2026. The copyright holder for this preprint (which was not certified by peer review) is the author/funder, who has granted bioRxiv a license to display the preprint in perpetuity. It is made available under aCC-BY 4.0 International license.

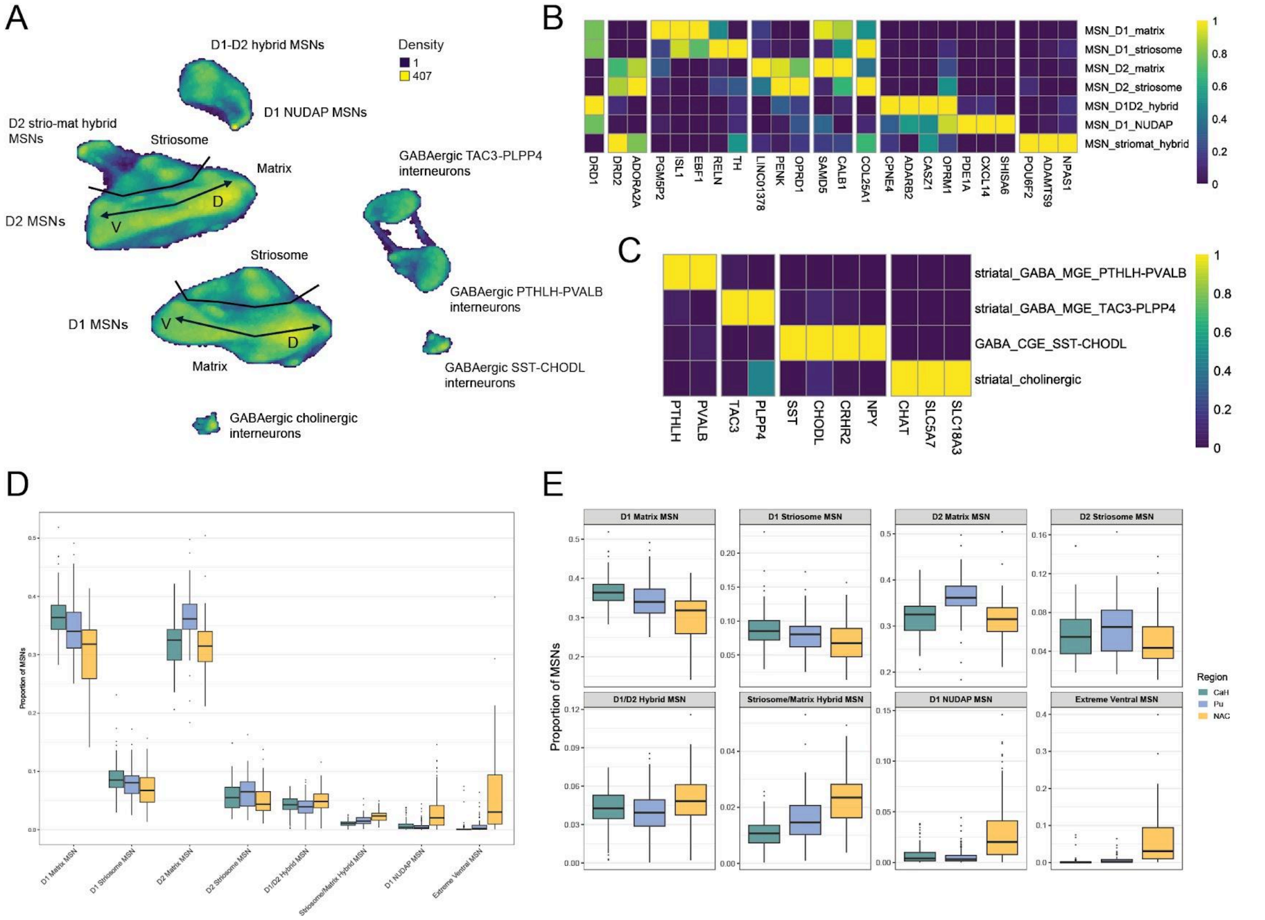

Figure S5. Identification and regional composition of striatal neuron subtypes.
A. Unsupervised clustering of the gene-expression profiles from striatal neurons identified all expected types and sub-types for anterior sampling of the striatum. For canonical (non-eccentric/hybrid) MSNs, boundaries between matrix and striosome are indicated with black lines and labels. Transcriptional differences between dorsal (D; CaH, Pu) and ventral (V; NAC) MSNs are also represented in this UMAP projection and the resulting gradients marked with black arrows. **B.** Expression levels of specific marker genes (columns) for MSN sub-types (rows). Colors represent expression levels that have been normalized by column (i.e., across the SPN types shown) such that highest expression is 1 (bright yellow) and lowest is 0 (dark purple). **C.** Expression levels of specific marker genes (columns) in striatal interneuron sub-types (rows), with expression levels represented by colors in the same way as panel **B**. **D.** D1 and D2 matrix MSNs make up the majority of MSNs in the striatum. **E.** Distributions of MSN proportions in the striatum (free scales).

bioRxiv preprint doi: https://doi.org/10.64898/2026.03.20.713160; this version posted March 23, 2026. The copyright holder for this preprint (which was not certified by peer review) is the author/funder, who has granted bioRxiv a license to display the preprint in perpetuity. It is made available under aCC-BY 4.0 International license.

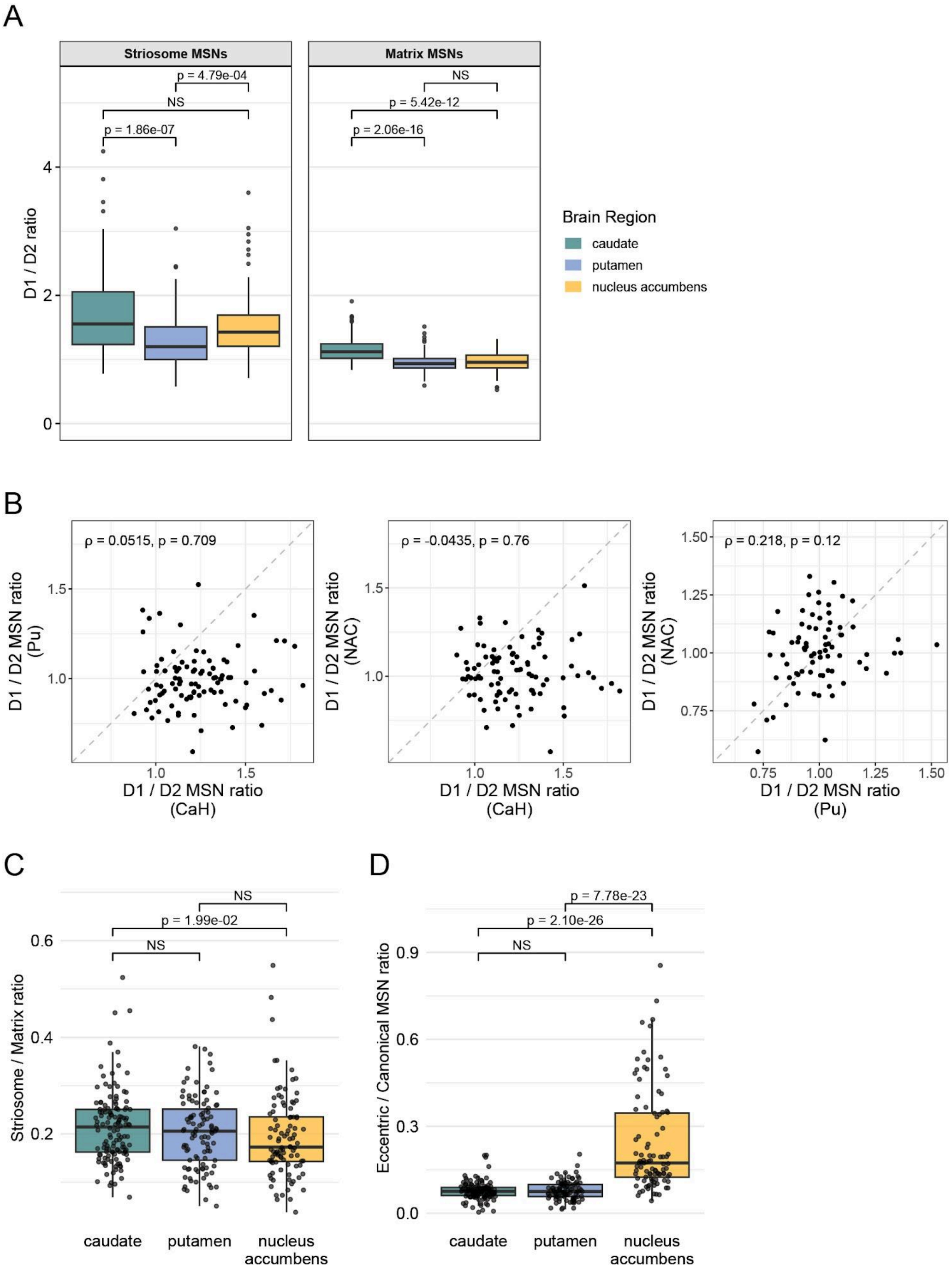

Figure S6. Representation of MSN subtypes across brain regions.
A. Comparison of D1 / D2 MSN ratios across CaH, Pu and NAC, stratified by striosome/matrix compartments and evaluated by Wilcoxon rank-sum test. **B:** Comparisons (and lack of significant correlations) between D1/D2 MSN ratios comparing different pairs of striatal regions. Spearman correlation coefficient and corrected p-values are shown (Benjamini-Hochberg (BH) procedure, n=498 tests). Left: caudate (x-axis) vs. putamen (y-axis), n=98 donors. Middle: caudate (x-axis) vs. nucleus accumbens (y-axis), n=90 donors. Right: putamen (x-axis) vs. nucleus accumbens (y-axis), n=78 donors. **C.** The striosome / matrix MSN ratio does not vary dramatically between regions, although there may be a slight striosome depletion in NAC, as evaluated by Wilcoxon rank-sum test. **D.** NAC is significantly enriched for eccentric MSNs relative to CaH and Pu, as evaluated by Wilcoxon rank-sum test.

bioRxiv preprint doi: https://doi.org/10.64898/2026.03.20.713160; this version posted March 23, 2026. The copyright holder for this preprint (which was not certified by peer review) is the author/funder, who has granted bioRxiv a license to display the preprint in perpetuity. It is made available under aCC-BY 4.0 International license.

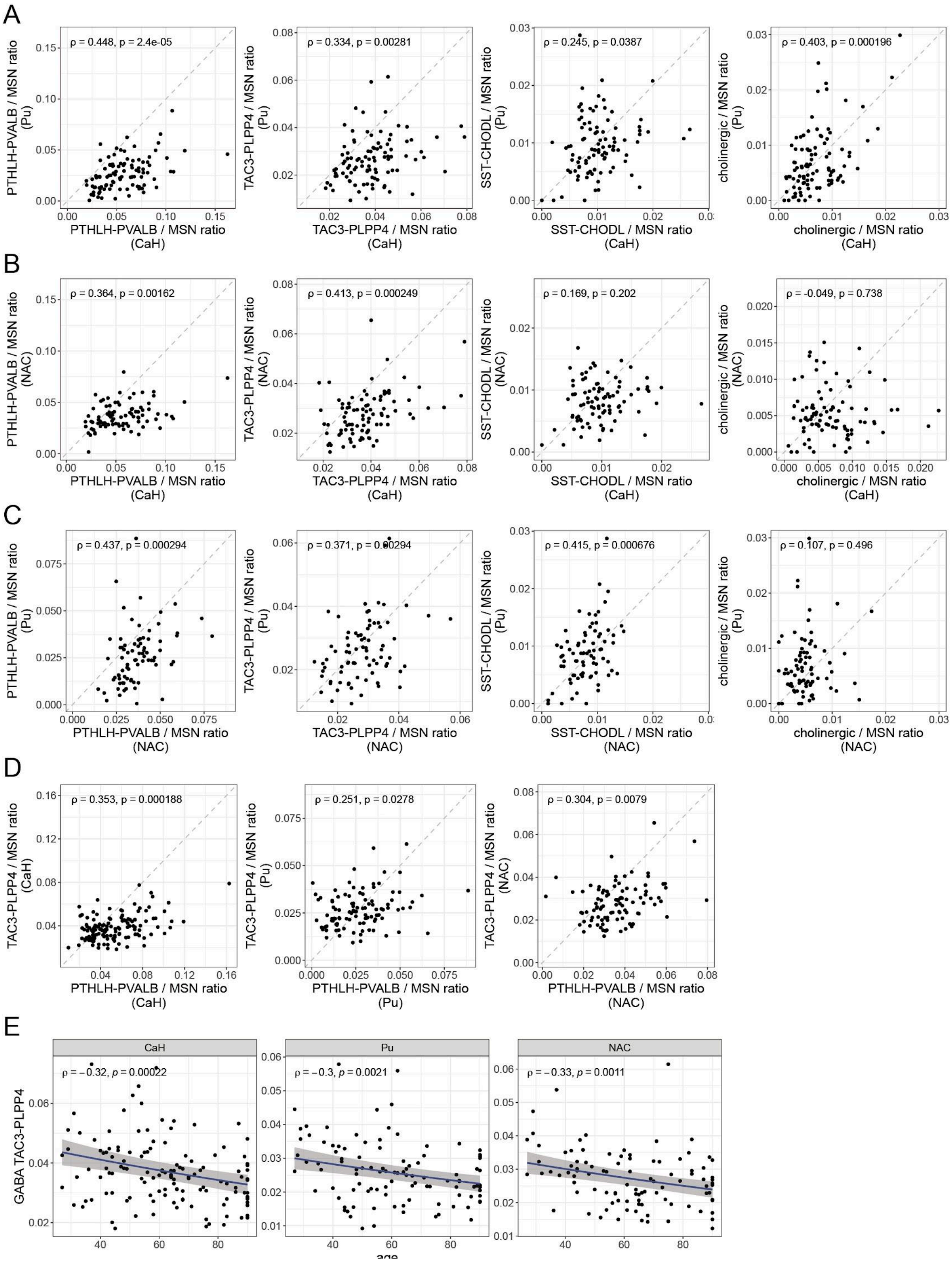

bioRxiv preprint doi: https://doi.org/10.64898/2026.03.20.713160; this version posted March 23, 2026. The copyright holder for this preprint (which was not certified by peer review) is the author/funder, who has granted bioRxiv a license to display the preprint in perpetuity. It is made available under aCC-BY 4.0 International license.

Figure S7. Relationships among the abundances of various types of striatal interneurons. In all plots, abundances have been quantified relative to the number of MSNs (in the same tissue sample), to reduce dependence of these measurements on one another and to minimize the effects of variance in glial populations. **A.** Abundances of various types of interneurons in the caudate (x-axis) versus putamen (y-axis) across 98 donors (points). Spearman correlation coefficient and corrected p-values are shown (Benjamini-Hochberg (BH) procedure, n=498 tests). PTHLH–PVALB, TAC3–PLPP4, and cholinergic interneuron subtype abundances were significantly correlated between caudate and putamen. **B.** Same analysis as in **A**, comparing caudate (x-axis) and nucleus accumbens (y-axis) across 90 donors. PTHLH-PVALB and TAC3-PLPP4 interneuron subtype abundances are significantly correlated. **C.** Same analysis as in **A**, comparing nucleus accumbens (x-axis) and putamen (y-axis) across 78 donors. PTHLH-PVALB, TAC3-PLPP4, and SST-CHODL interneuron subtype abundances were significantly correlated. **D.** Within-sample comparison of PTHLH–PVALB and TAC3–PLPP4 abundances. Left: caudate (n=131 donors). Middle: putamen (n=104 donors). Right: nucleus accumbens (n=97 donors). Spearman correlation coefficient and corrected p-values are shown (BH-procedure, n=498 tests) **E.** Attrition of TAC3–PLPP4 interneurons with advancing age, in all striatal gray matter regions. Abundance is expressed relative to all neurons. Each point represents a donor, and blue lines indicate beta-binomial fits with 95% confidence intervals (gray ribbons). Abundance is negatively correlated with age for all brain regions (Spearman correlation coefficients and nominal p-values shown). Modeling with a beta-binomial regression confirmed a significant decline with age, shown with the regression line (β = –0.047 per decade; 95% CI: –0.069 to –0.025; BH-adjusted p = 0.001; n=44 tests; **Table S4**).

bioRxiv preprint doi: https://doi.org/10.64898/2026.03.20.713160; this version posted March 23, 2026. The copyright holder for this preprint (which was not certified by peer review) is the author/funder, who has granted bioRxiv a license to display the preprint in perpetuity. It is made available under aCC-BY 4.0 International license.

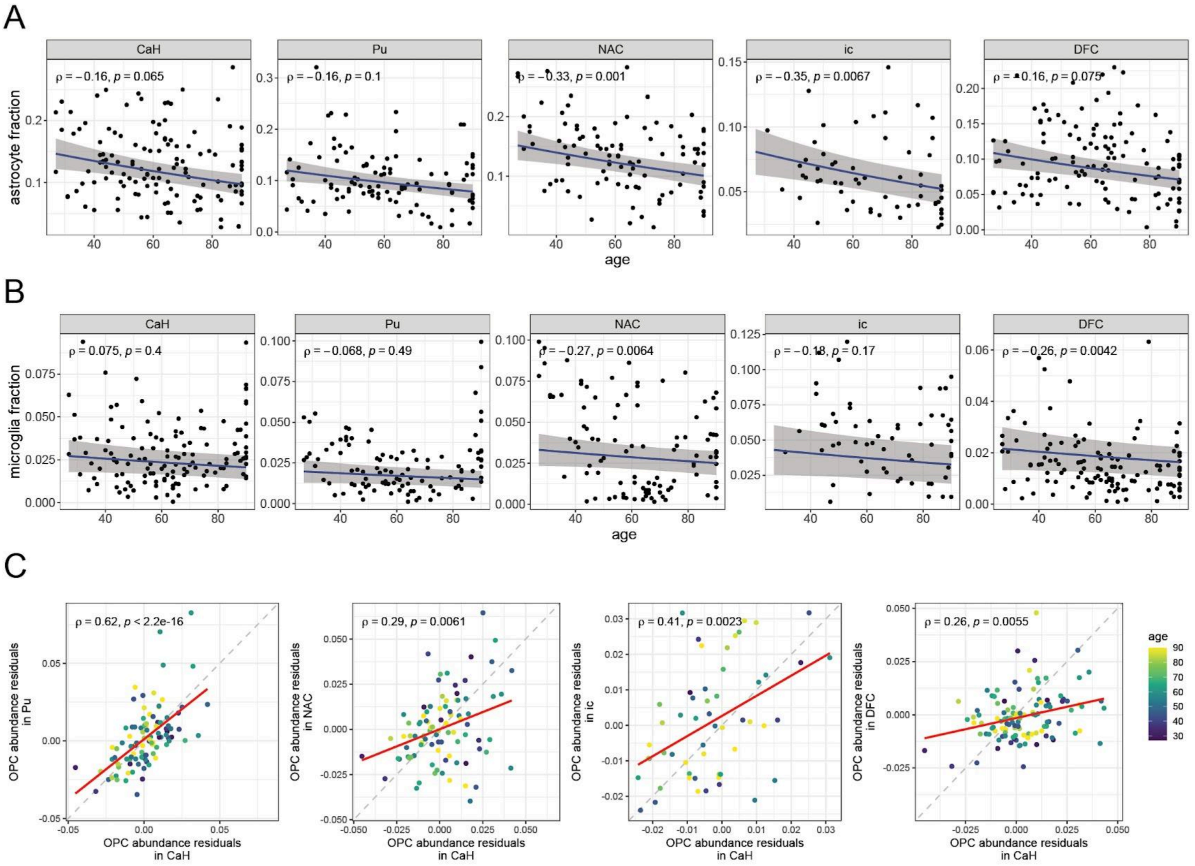

Figure S8. Additional analyses of glial cell abundances in relationship to age.
A. Abundance of astrocytes, as a fraction of all nuclei sampled. Each point represents a donor, and blue lines indicate beta-binomial fits with 95% confidence intervals (gray ribbons). While nominally significant negative Spearman correlations were observed in specific regions, the result of global modeling via beta-binomial regression (β = –0.074 per decade; 95% CI: –0.115 to –0.033; BH-adjusted p = 0.018; n=44 tests; **Table S4**) was not significant after correcting for multiple hypothesis testing; however, this is not a definitive negative result. **B.** Same as **A**, but for microglia abundance. Beta-binomial regression found no significant association between microglial abundance and age (β = –0.045 per decade; BH-adjusted p = 1; n=44 tests; **Table S4**). **C.** Comparison of OPC fraction residuals (observed minus age-predicted values from covariate-adjusted beta-binomial models) for the caudate compared to the other four brain regions analyzed. Each point represents a donor, colored by age; red lines indicate linear regression fits. The positive correlations in these residuals across all pairs of brain regions indicate that additional donor-level factors (beyond chronological age) influence OPC abundance in the striatum complex and DFC in a similar manner.

bioRxiv preprint doi: https://doi.org/10.64898/2026.03.20.713160; this version posted March 23, 2026. The copyright holder for this preprint (which was not certified by peer review) is the author/funder, who has granted bioRxiv a license to display the preprint in perpetuity. It is made available under aCC-BY 4.0 International license.

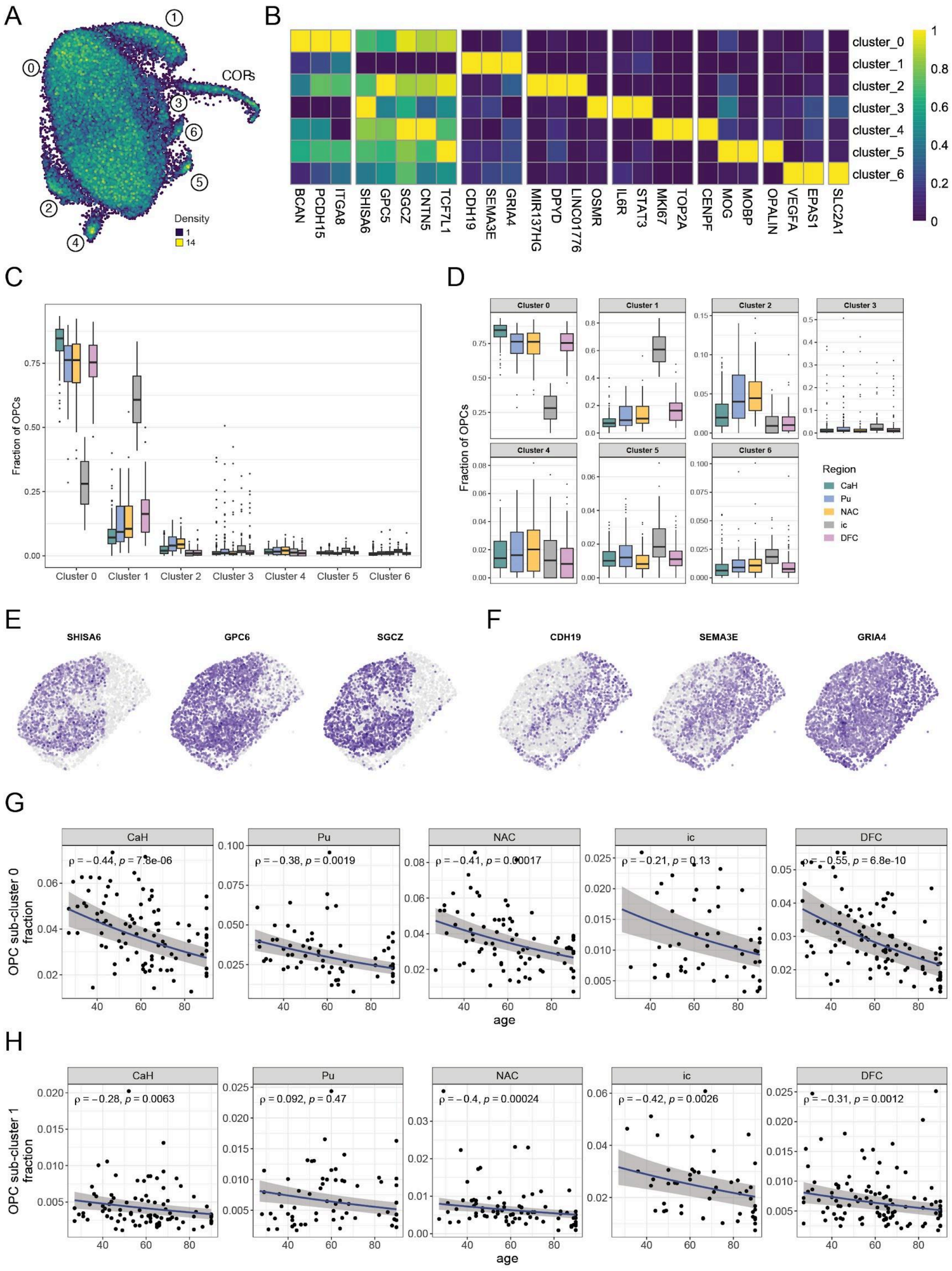

bioRxiv preprint doi: https://doi.org/10.64898/2026.03.20.713160; this version posted March 23, 2026. The copyright holder for this preprint (which was not certified by peer review) is the author/funder, who has granted bioRxiv a license to display the preprint in perpetuity. It is made available under aCC-BY 4.0 International license.

Figure S9. OPC diversity and attrition with age.
A. Unsupervised clustering assignments and UMAP representation of OPCs sampled from the caudate. Colors on the UMAP plot represent the density of nuclei in that region of the plot. **B.** Expression levels of specific marker genes (columns) for OPC sub-clusters (rows; note, COPs excluded). Colors represent normalized expression by column (across major cell classes) such that the highest expression is 1 (bright yellow) and lowest is 0 (dark purple). **C.** Distribution of OPC sub-cluster compositions across regions. Gray matter regions exhibit much lower numbers of cluster 1 OPCs and higher numbers of cluster 0 OPCs relative to the internal capsule. **D.** Distribution of OPC sub-cluster compositions across regions (free scales). **E,F.** Slide-tags spatial transcriptomics analysis of the striatum from a representative donor. Expression of genes whose expression defines OPC cluster 0 (e.g. *SHISA6*, *GPC6,* and *SGCZ*) highlights gray matter compartments. Color of the points represents log-normalized transcript counts for each gene (light grey lower expression, dark purple higher expression). Expression of genes whose expression defines OPC cluster 1 (*CDH19, SEMA3E*) highlights white matter compartments. *GRIA4* is expressed in all OPC sub-clusters, but most strongly in cluster 1, as seen in **B. G.** Decline in abundance of OPC cluster 0 with advancing age, across the brain regions sampled. Abundance is expressed relative to all nuclei sampled. Each point represents a donor, and blue lines indicate beta-binomial fits with 95% confidence intervals (gray ribbons). Spearman correlation coefficients and nominal p-values shown. Modeling with a beta-binomial regression confirmed a significant decline with age (β = –0.095 per decade; 95% CI: –0.122 to –0.067; BH-adjusted p = 7.02e-10; n=44 tests; **Table S4**). **H.** Same as **G**, but for OPC cluster 1. Modeling with a beta-binomial regression confirmed a significant decline with age (β = –0.072 per decade; 95% CI: –0.109 to –0.036; BH-adjusted p = 0.004; n=44 tests; **Table S4**).

bioRxiv preprint doi: https://doi.org/10.64898/2026.03.20.713160; this version posted March 23, 2026. The copyright holder for this preprint (which was not certified by peer review) is the author/funder, who has granted bioRxiv a license to display the preprint in perpetuity. It is made available under aCC-BY 4.0 International license.

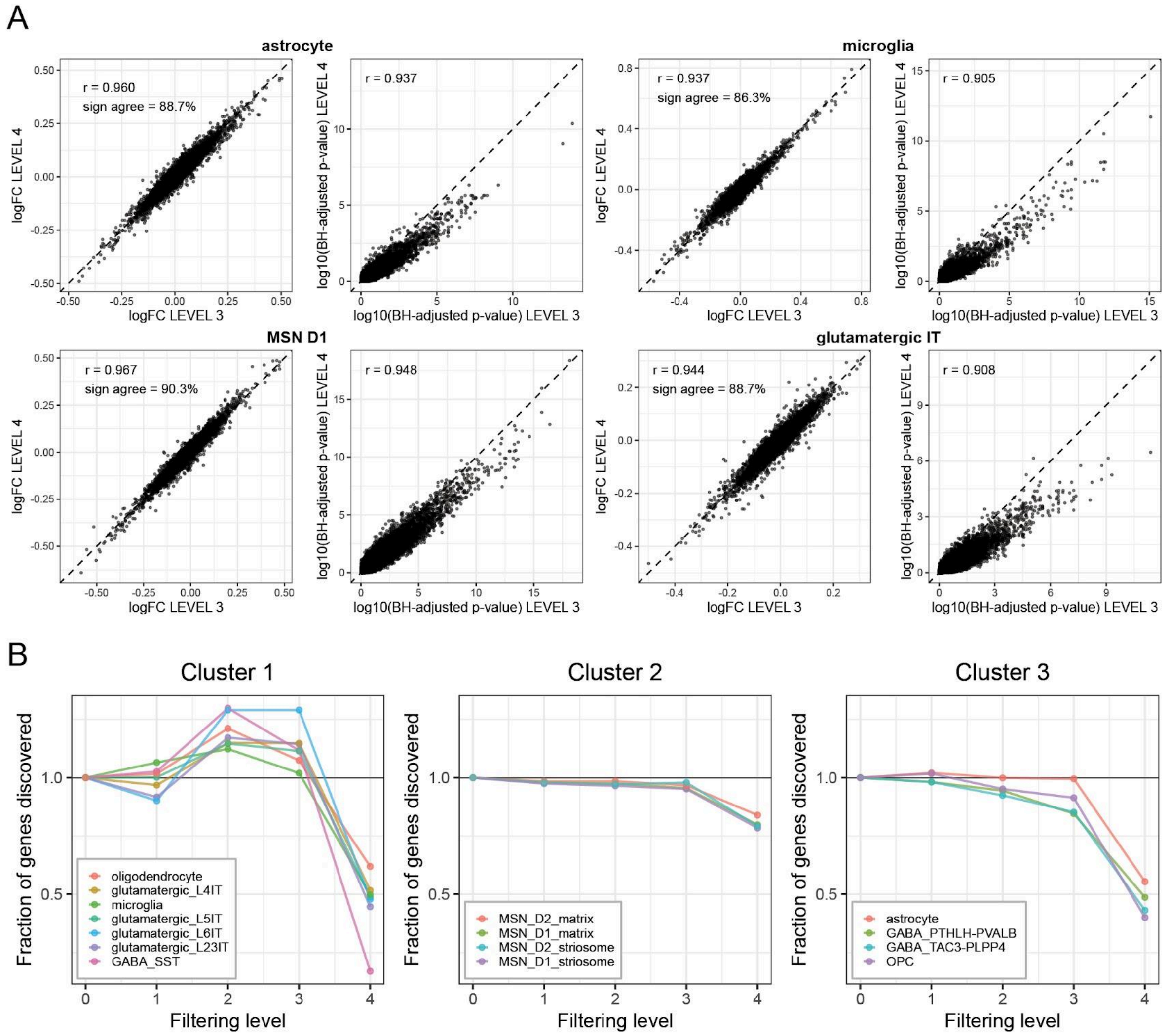

Figure S10. Effects of donor exclusion criteria on recognition and inference of age-associated changes. A. Comparison, in multiple cell types, of gene-level log fold-change estimates and statistical significance (–log10 FDR-adjusted p-values) for age-associated genes as donor exclusion criteria are made more stringent. Stringency levels 0–3 represent progressively stricter snRNA-seq-data-driven exclusions; Level 4 additionally excludes donors based on metadata criteria from clinical records. Effect-size estimates remain highly concordant across filtering levels, and statistical significance is largely preserved or improved under snRNA-seq-data-driven filtering. In contrast, adding metadata-driven exclusions reduces statistical significance (and thus ability to distinguish real effects at genome-wide significance) while leaving effect-size estimates largely unchanged. **B.** Fraction of age-associated differentially expressed genes detected at each filtering level, normalized to the unfiltered dataset (Level 0), shown across representative cell types. Cell types were clustered using k-means clustering (k=3) based on the fraction of genes discovered at each filtering level to identify shared patterns. snRNAseq-data-driven filtering (Levels 1–3) preserved or increased the number of discoveries for nearly all cell types. Additional metadata-driven exclusions (Level 4) reduced the number of detected genes across cell types, indicating a loss of statistical power without changes in estimated effect sizes.

bioRxiv preprint doi: https://doi.org/10.64898/2026.03.20.713160; this version posted March 23, 2026. The copyright holder for this preprint (which was not certified by peer review) is the author/funder, who has granted bioRxiv a license to display the preprint in perpetuity. It is made available under aCC-BY 4.0 International license.

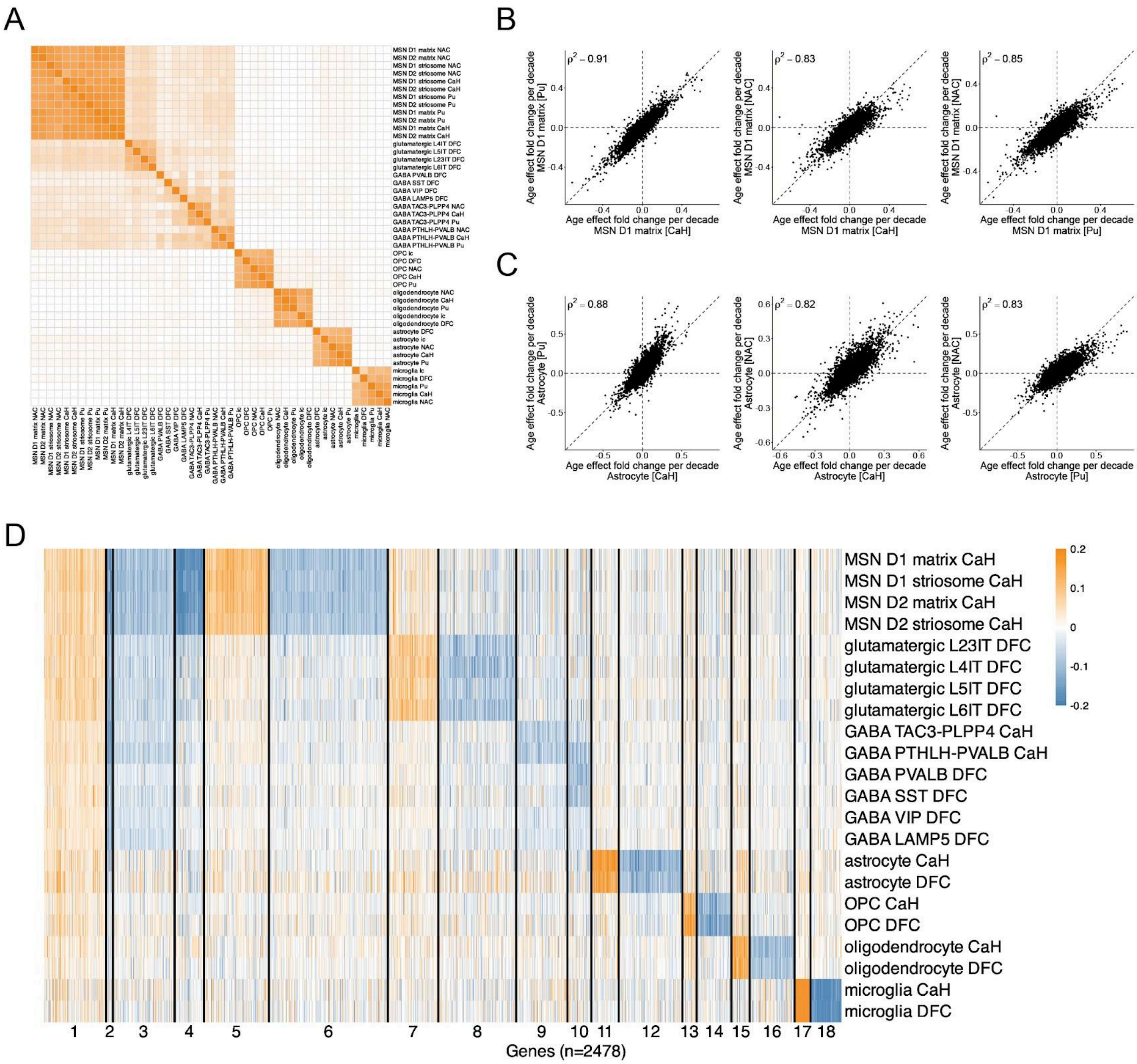

Figure S11. Extended analyses of aging effects. Same analyses as in **Figure 4**, but extended to additional cell populations. **A.** Heatmap shows correlation of age-associated gene-expression changes for each pair of cell types and brain regions. Colors show Spearman's ρ2 for correlations of gene-level log2-fold-change per decade of age. **B.** Comparison of D1 MSNs (matrix subtype) age-associated gene-expression changes between CaH and Pu, CaH and NAC, and Pu and NAC. **C.** Comparison of astrocyte age-associated gene-expression changes between CaH and Pu, CaH and NAC, and Pu and NAC. **D.** Clustering of genes by their patterns of age-associated expression changes in the various cell types and brain regions. Columns show genes (n = 2478) grouped by k-means clustering (same clustering plotted in **Figure 4H**) of their age-associated expression changes (log2-fold-change per decade of age) across the various cell types

bioRxiv preprint doi: https://doi.org/10.64898/2026.03.20.713160; this version posted March 23, 2026. The copyright holder for this preprint (which was not certified by peer review) is the author/funder, who has granted bioRxiv a license to display the preprint in perpetuity. It is made available under aCC-BY 4.0 International license.

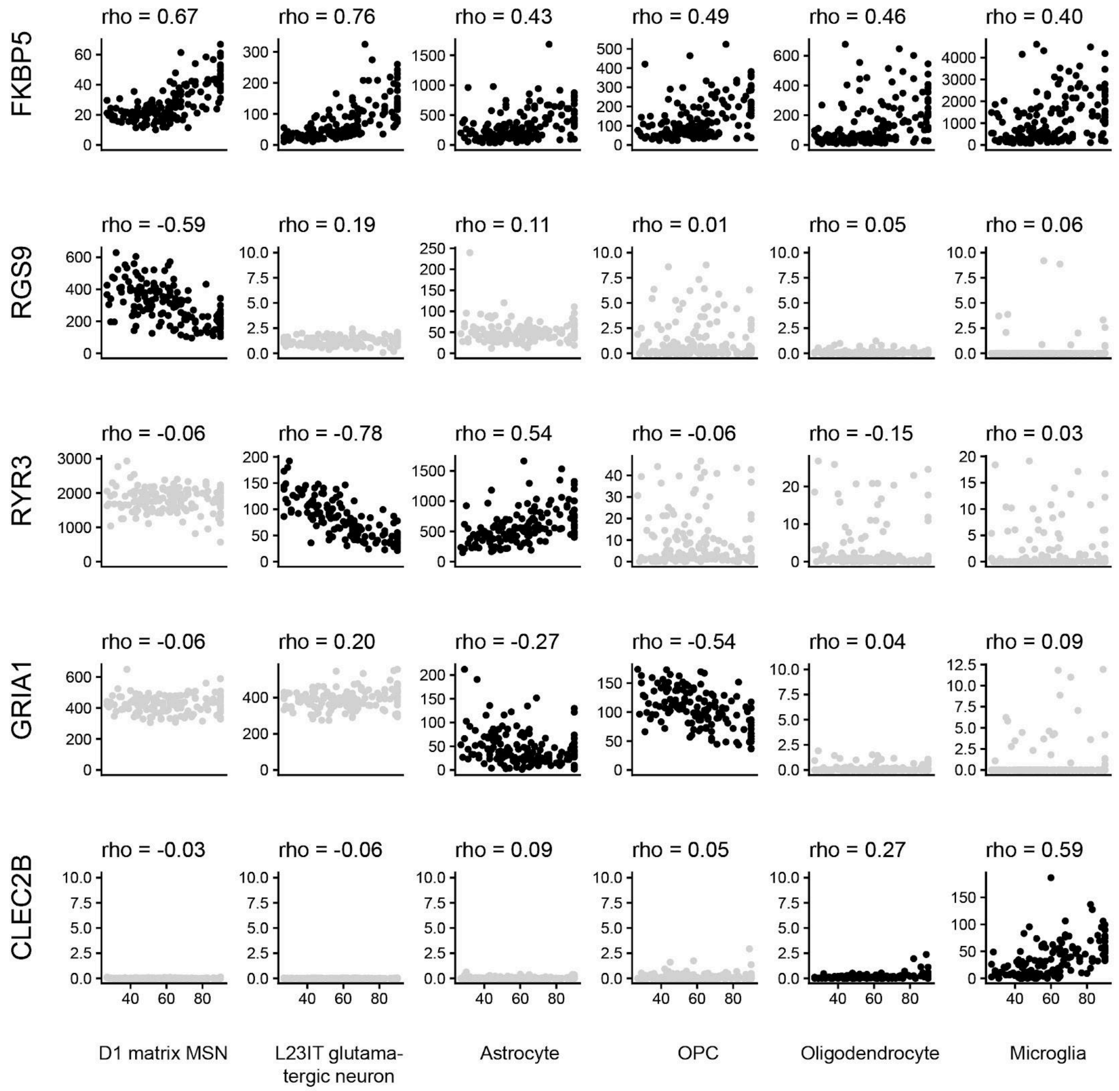

Figure S12. Age-associated effects on expression of specific genes.
Scatterplots show expression of specific genes in relationship to donor age (x-axis of each plot); expression levels have been normalized (transcripts per million; y-axis of each plot). Spearman's ρ, a non-parametric statistic assessing correlation between donor age and expression level (for the selected gene, cell type) is shown above each plot. For non-significant correlations (nominal p-value > 0.05) points are shown in grey.

bioRxiv preprint doi: https://doi.org/10.64898/2026.03.20.713160; this version posted March 23, 2026. The copyright holder for this preprint (which was not certified by peer review) is the author/funder, who has granted bioRxiv a license to display the preprint in perpetuity. It is made available under aCC-BY 4.0 International license.

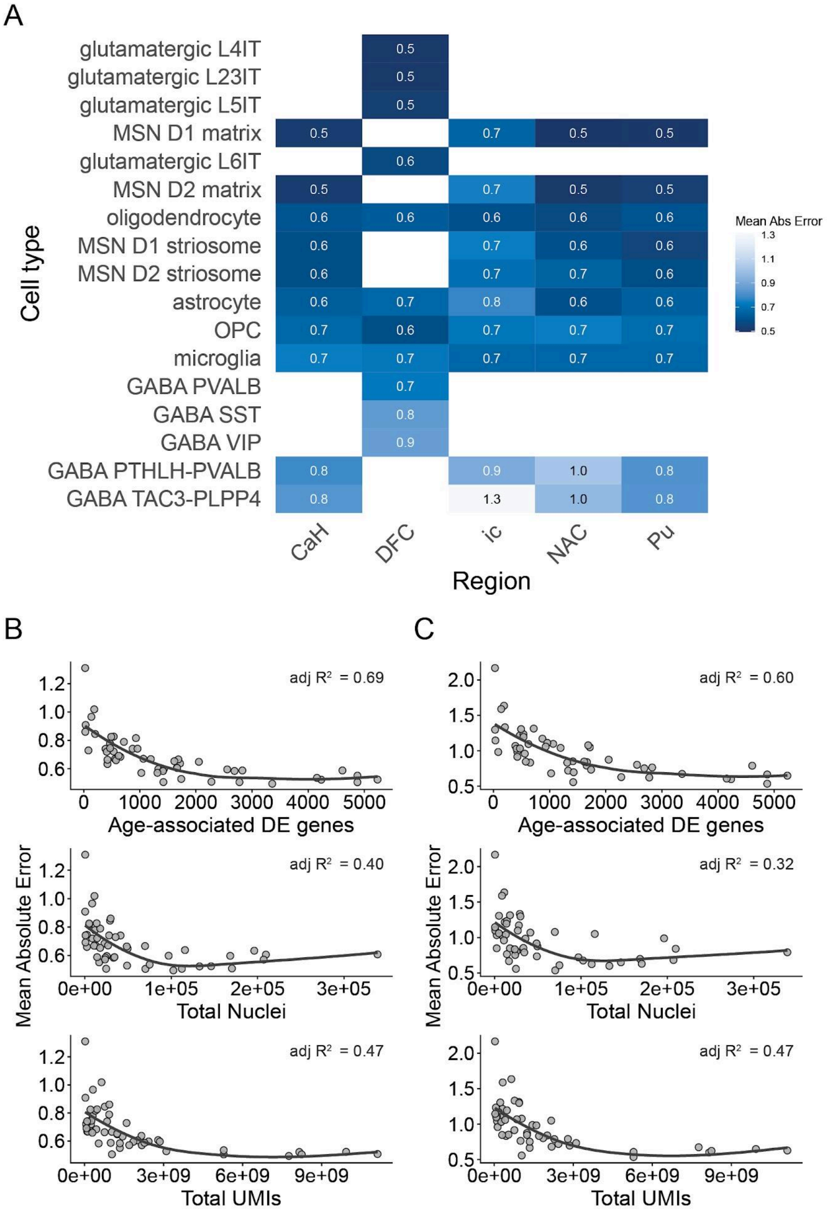

Figure S13. Mean absolute error for age prediction models.

A. Heatmap showing mean absolute error for each cell type–region age-prediction model. Cell-type/region combinations for which age-prediction models were built are shown in shade of blue, with the shade reporting the model error.. **B.** Mean absolute error plotted against the number of age-associated differentially expressed genes, total nuclei, and total UMIs per model. Adjusted R^2 values are calculated separately for each predictor. A multivariable model including all three predictors explains variation in mean absolute error across models (adjusted $R^2 \approx 0.72$), indicating that prediction accuracy reflects both power to detect age-associated genes and cell-type-specific differences in the magnitude of age-related transcriptional effects. **C.** The analysis in B was repeated using only the youngest 20% of donors. Mean absolute error is higher than in the full cohort, reflecting regression-to-the-mean effects, but shows similar dependence on the predictors.

bioRxiv preprint doi: https://doi.org/10.64898/2026.03.20.713160; this version posted March 23, 2026. The copyright holder for this preprint (which was not certified by peer review) is the author/funder, who has granted bioRxiv a license to display the preprint in perpetuity. It is made available under aCC-BY 4.0 International license.

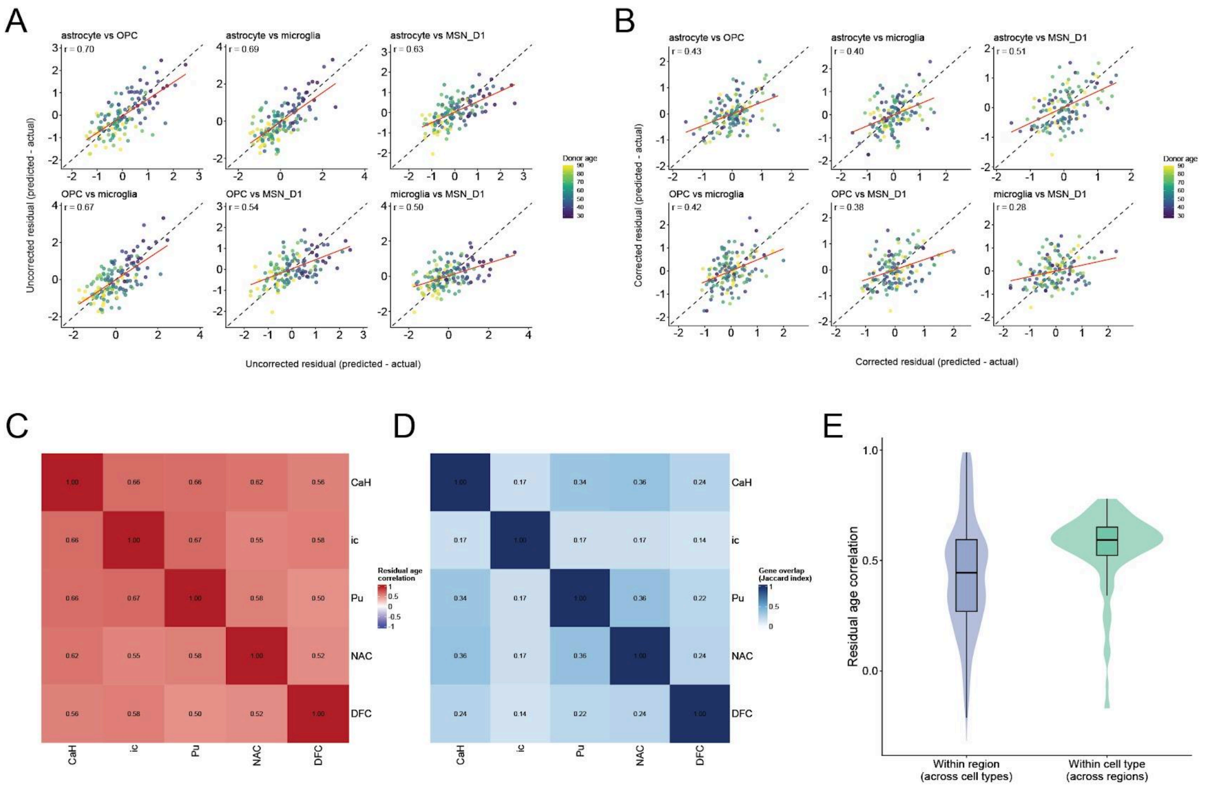

Figure S14. Correction and correlation of age prediction residuals.
A. Pairwise comparisons of uncorrected age residuals (predicted minus chronological age) between representative caudate cell types. Residuals were found to be positively correlated across cell types. Regression-to-the-mean effects can introduce age-dependent structure in these residuals, thereby inflating correlation across cell types. **B.** The same pairwise comparisons as D after GAM-based correction of predicted versus chronological age. Correlations are reduced relative to the uncorrected residuals, indicating removal of shared age-dependent bias. A non-zero correlation signal remains, consistent with shared inter-individual variation in biological aging beyond regression-to-the-mean effects. **C**. Positive correlation of cell-type-specific RNA-expression "clocks", beyond the shared effects of chronological age. The heatmap shows pairwise correlations (for each pair of caudate cell types) of the GAM-corrected age residuals. **D**. Minimal overlap of the genes used to predict age in most cell types. The heatmap shows Jaccard indices quantifying overlap among age-associated genes used in each cell-type-specific model. **E.** Pairwise correlations of corrected residual age were computed across cell types within each region and across regions within each cell type. On average, correlations were higher across regions for a given cell type than across cell types within a region, indicating that biological aging kinetics are more strongly shared within cell type (across brain regions) than within region (across cell types).

bioRxiv preprint doi: https://doi.org/10.64898/2026.03.20.713160; this version posted March 23, 2026. The copyright holder for this preprint (which was not certified by peer review) is the author/funder, who has granted bioRxiv a license to display the preprint in perpetuity. It is made available under aCC-BY 4.0 International license.

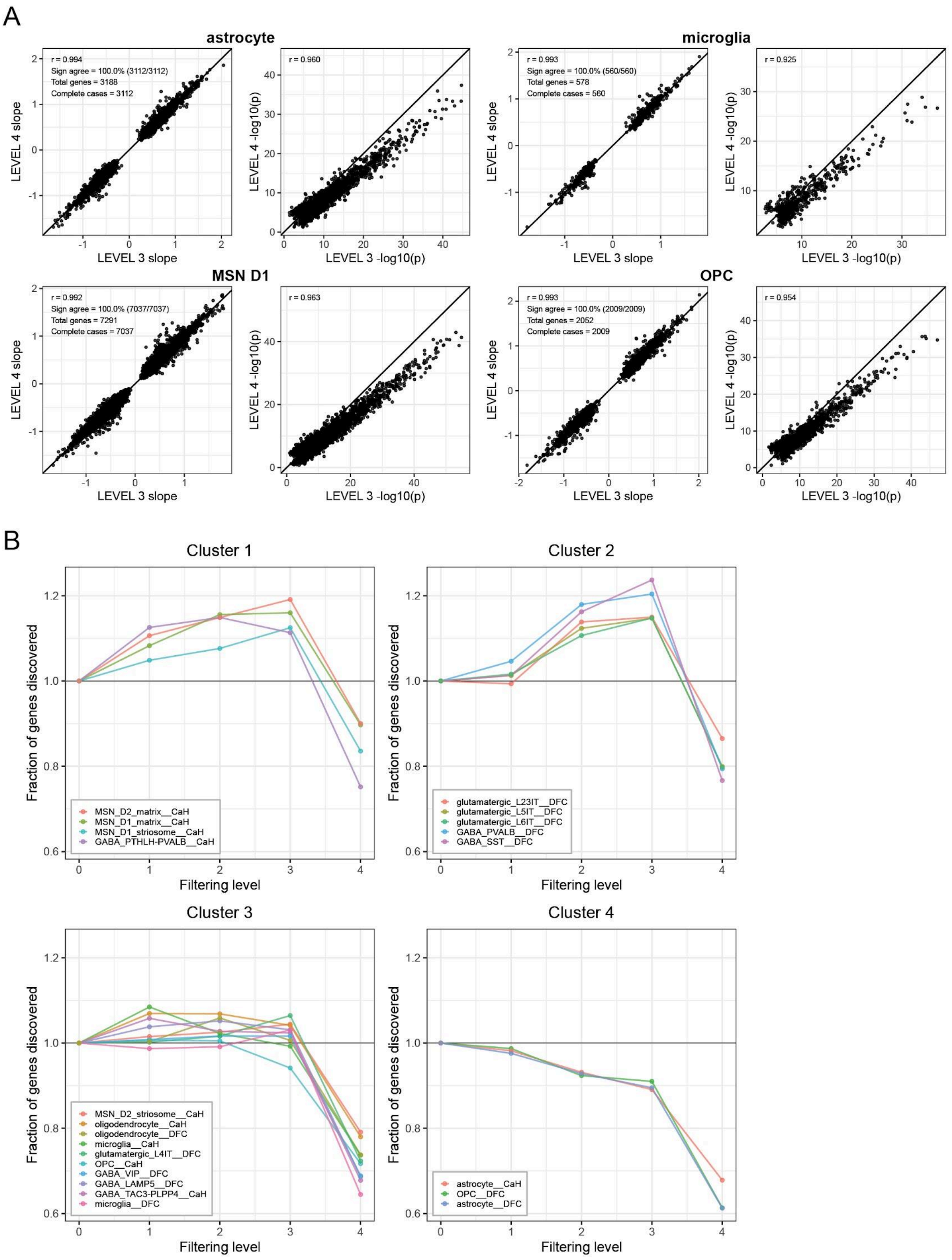

bioRxiv preprint doi: https://doi.org/10.64898/2026.03.20.713160; this version posted March 23, 2026. The copyright holder for this preprint (which was not certified by peer review) is the author/funder, who has granted bioRxiv a license to display the preprint in perpetuity. It is made available under aCC-BY 4.0 International license.

Figure S15. Effects of donor exclusion criteria on recognition and inference of eQTLs. A. Comparison of log fold-change estimates and statistical significance (–log10 empiric p-values) for expression quantitative trait loci (eQTL) discovery. As in the earlier analysis of age-associated effects (**Figure S10**), Levels 0–3 represent progressively stricter snRNA-seq-data-driven exclusions; Level 4 additionally excludes donors based on metadata criteria (findings in medical histories). Effect-size estimates remained highly concordant across filtering levels, and statistical significance is largely preserved or improved under snRNA-seq-data-driven filtering. In contrast, adding metadata-driven exclusions reduced statistical significance while effect-size estimates were largely unchanged. Annotated are the correlation of effect sizes, fraction of the time the direction of the eQTL effect sizes agree (sign test), total number of genes compared (union of eGenes), and total number of genes discovered in both data sets (intersect of eGenes.) **B.** Fractions of eGenes detected at each filtering level, normalized to the unfiltered dataset (Level 0), shown across representative cell types. Cell types were clustered using k-means clustering (k=4) based on the fraction of genes discovered at each filtering level to identify shared patterns. snRNA-seq-data-driven filtering (Levels 1–3) preserved or increased the number of discoveries. Additional metadata-driven exclusions (Level 4) reduced the number of detected genes across cell types, indicating a loss of statistical power without corresponding changes in estimated effect sizes.

bioRxiv preprint doi: https://doi.org/10.64898/2026.03.20.713160; this version posted March 23, 2026. The copyright holder for this preprint (which was not certified by peer review) is the author/funder, who has granted bioRxiv a license to display the preprint in perpetuity. It is made available under aCC-BY 4.0 International license.

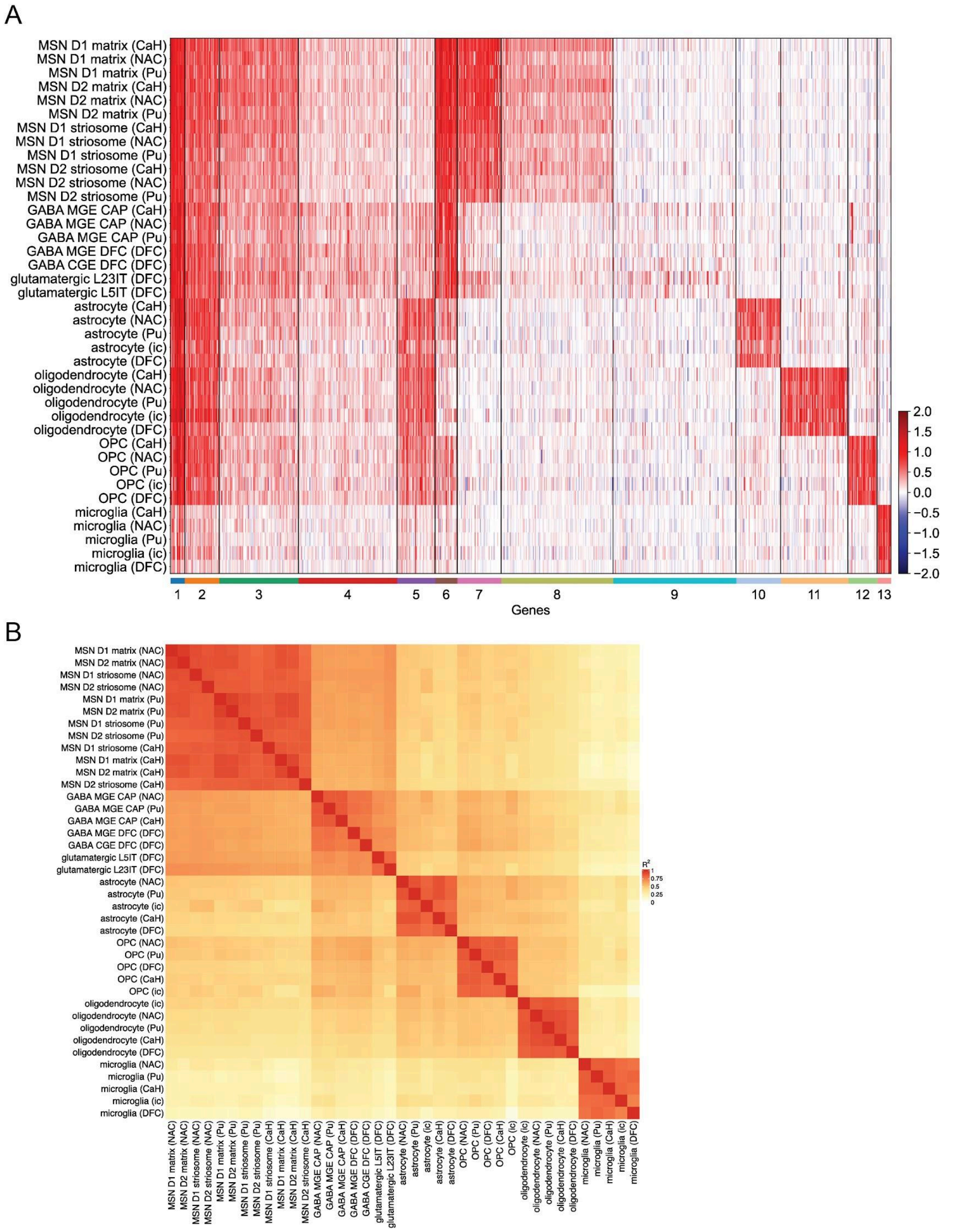

Figure S16. Cell-type specificity of expression QTLs. Extension of Figure 6 to additional brain regions. **A.** K-means clustering (K=13) of the same 9,899 expression QTLs as shown in Fig. 6B (reaching significance in either CaH, DFC, or both regions), using effect sizes estimated across five brain regions (CaH, Pu, NAC, ic, DFC). Rows represent separate region-specific analyses of eQTLs in each cell type, and columns represent these eQTLs (gene-SNP pairs) grouped by cluster. Shades of red and blue indicate standardized effect sizes (inverse-normalized log2-transformed expression), with red indicating the predominant direction of effect and blue indicating opposite-direction effects. **B.** Pairwise correlations (among cell types sampled in each brain region) of genome-wide sets eQTL effects (corresponding to the rows in panel A). For each cell-type pair, Spearman correlation (R^2) was calculated using eQTLs significant in at least one of the two cell types. Rows and columns were hierarchically clustered.

bioRxiv preprint doi: https://doi.org/10.64898/2026.03.20.713160; this version posted March 23, 2026. The copyright holder for this preprint (which was not certified by peer review) is the author/funder, who has granted bioRxiv a license to display the preprint in perpetuity. It is made available under aCC-BY 4.0 International license.

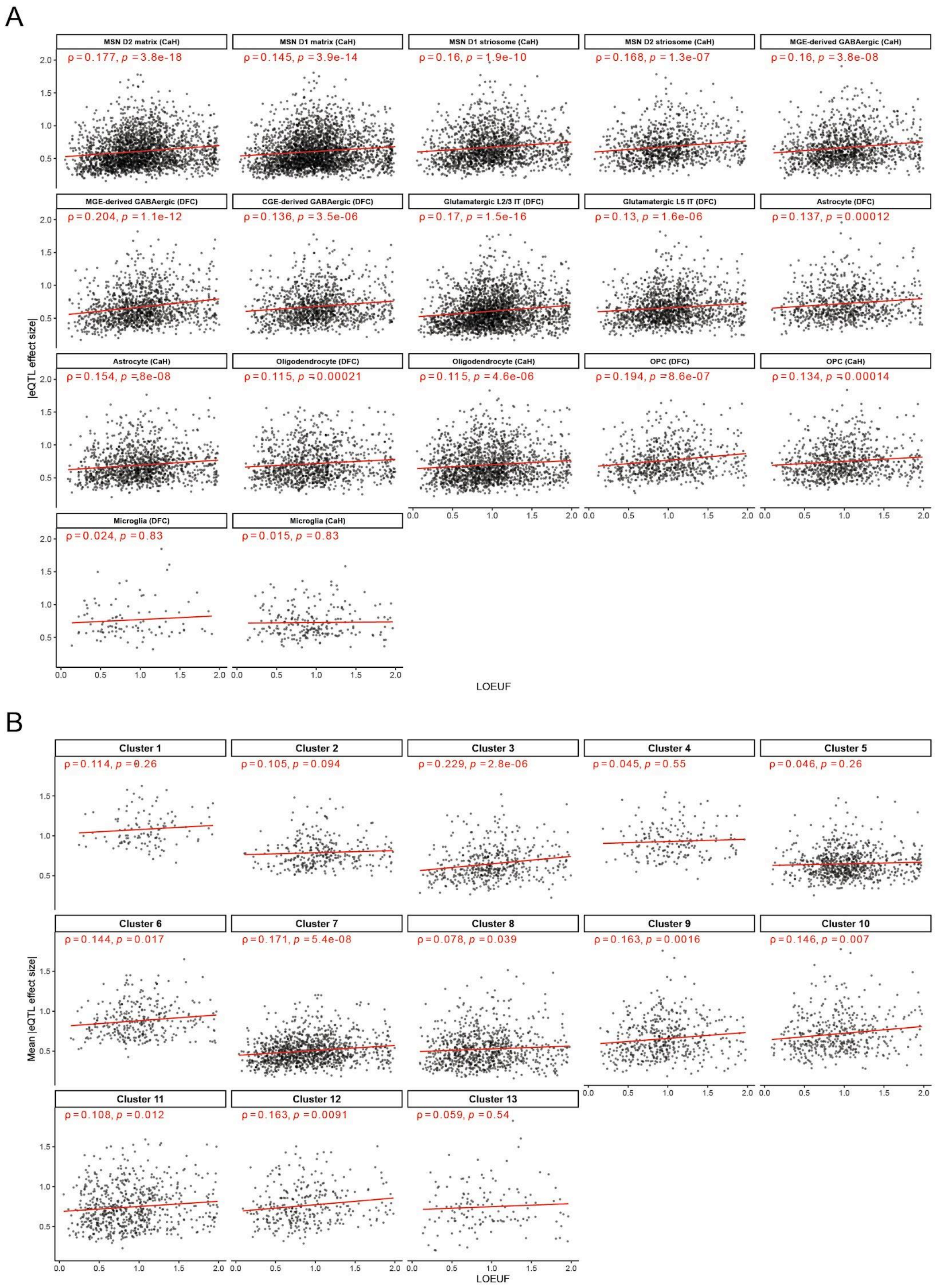

bioRxiv preprint doi: https://doi.org/10.64898/2026.03.20.713160; this version posted March 23, 2026. The copyright holder for this preprint (which was not certified by peer review) is the author/funder, who has granted bioRxiv a license to display the preprint in perpetuity. It is made available under aCC-BY 4.0 International license.

Figure S17: Relationship of eQTL effect sizes and gene function constraint.
eQTL effect sizes are measured as the change in gene expression per allele inherited; functional constraint is quantified by LOEUF scores (Loss-of-function Observed/Expected Upper bound Fraction). Genes' LOEUF scores have been measured in earlier work [68] and are based on the frequency (relative to chance expectation) with which genes are found to have loss-of-function mutations in human populations. Genes with low LOEUF scores have very few such mutations and are thus inferred to be under strong functional constraint (such as haploinsufficiency). **A.** Scatter plots showing the relationship of absolute eQTL effect sizes (y-axis) to LOEUF scores (x-axis) for each of 17 combinations of cell type and brain region for which eQTLs were recognized in independent analyses. **B.** Mean absolute eQTL effect sizes across statistically significant cell types for each K-means eQTL cluster (K=13). Analyses included all protein-coding eGenes ($q < 0.01$) with LOEUF scores from gnomAD v4.1. Higher LOEUF indicates less constraint (greater tolerance to loss-of-function). Red lines show linear regression fits; Spearman ρ and FDR-adjusted p-values are shown per cluster. Effect sizes were measured from tensorQTL slopes (relationship of inverse-normal-transformed expression level to genotype).

bioRxiv preprint doi: https://doi.org/10.64898/2026.03.20.713160; this version posted March 23, 2026. The copyright holder for this preprint (which was not certified by peer review) is the author/funder, who has granted bioRxiv a license to display the preprint in perpetuity. It is made available under aCC-BY 4.0 International license.

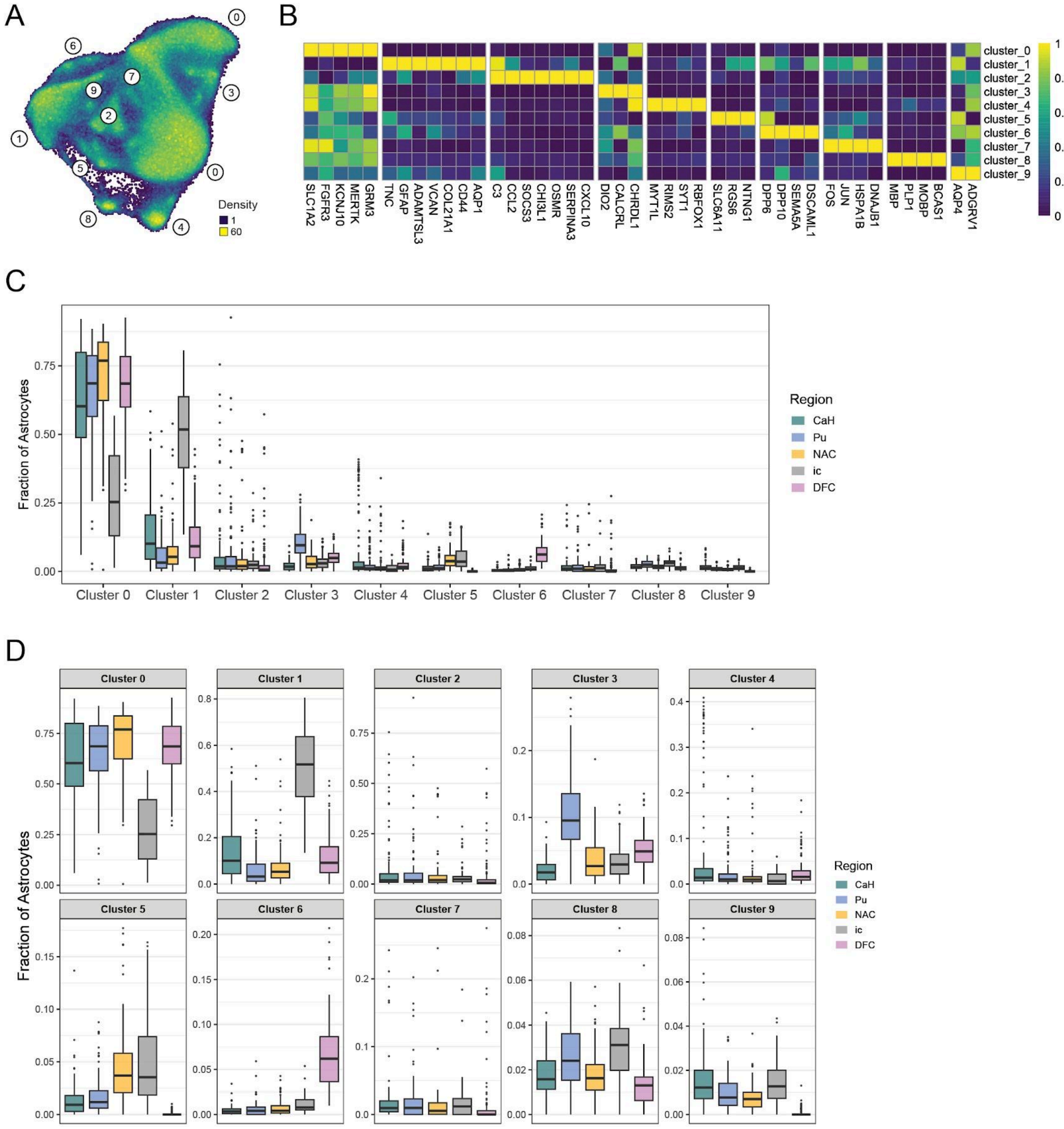

Figure S18: Astrocyte sub-cluster identification and abundance.
A. Unsupervised clustering assignments and UMAP representation of astrocytes across striatum and cortex samples. UMAP coloring represents spatial density of nuclei. **B.** Selected marker genes (columns) for astrocyte sub-clusters (rows). Colors represent normalized expression by column (across major cell classes) such that heist expression is 1 (bright yellow) and lowest is 0 (dark purple). **C.** Distribution of astrocyte sub-cluster compositions across regions. Gray matter regions exhibit cluster 1 astrocyte depletion and cluster 0 astrocyte enrichment relative to the internal capsule. **D.** Distribution of astrocyte sub-cluster compositions across regions (free scales)

bioRxiv preprint doi: https://doi.org/10.64898/2026.03.20.713160; this version posted March 23, 2026. The copyright holder for this preprint (which was not certified by peer review) is the author/funder, who has granted bioRxiv a license to display the preprint in perpetuity. It is made available under aCC-BY 4.0 International license.

Supplemental tables

Table S1: Donor stratification and analysis inclusion by diagnostic "exclusion" category, age, and sex. Donor counts by clinical/neuropathological diagnostic "exclusion" categories, age groups (<50 years, >50 years), and sex. Diagnostic "exclusion" categories include overdose/substance dependence, psychiatric conditions, neurodegeneration, and cases with missing clinical brain diagnosis (CBD) or neuropathology (NP). The table also indicates the inclusion of each donor group in downstream analyses.

Table S2: Donor demographics. Comprehensive donor-level metadata, including demographic information, clinical and neuropathological diagnoses, tissue source, and cohort inclusion criteria. This table also indicates donor inclusion in downstream analyses.

Table S3: Sample covariates. Description of demographic, genetic, technical, and sequencing-quality variables.

Table S4: Beta-binomial regression results for age and sex effects on cell-type abundances. Effect sizes, standard errors, test statistics, and p-values for age and sex effects on cell-type abundances estimated using beta-binomial regression. Abundances are defined relative to the indicated denominator (e.g., all nuclei). Age is modeled per decade, and sex is coded as male versus female. Effect sizes are reported on the log-odds scale, with positive values indicating increased relative abundance and negative values indicating decreased relative abundance with the covariate. Nominal p-values and p-values adjusted for multiple comparisons using the Benjamini–Hochberg procedure are reported.

Table S5: Significant age-associated differential expression Gene Set Enrichment Analysis (GSEA) results by cell type. Counts of unique eGenes identified per cell type, defined as the union of eGenes detected in dorsolateral prefrontal cortex (DFC) and caudate head (CaH).

Table S6: Union of eGenes across DFC and CaH by cell type. Counts of unique eGenes identified per cell type, defined as the union of eGenes detected in dorsolateral prefrontal cortex (DFC) and caudate head (CaH).

Table S7: Fraction of cell-type-specific eGenes with maximum expression in corresponding cell type(s). For each eGene cluster (k-means; see **Figure 6**), the expected cell type(s) (by region), the number of eGenes with nonzero median expression across cell types, the number of eGenes whose highest median expression occurs in one of the corresponding cell types, and the proportion of eGenes whose maximum median expression falls in one of the expected cell type(s) (see **Methods**).

Methods

Donor selection and tissue procurement

Postmortem human brain tissue was obtained through the Human Brain Cell Atlas Collection (HBCAC), a dedicated program within the NIH NeuroBioBank (NBB; RRID:SCR_003131) supporting the NIH BRAIN Initiative Cell Atlas Network (BICAN). Brain and Tissue Repositories (BTRs) contributing to the NBB HBCAC followed harmonized protocols for donor evaluation, brain collection, and tissue processing and preparation such as photodocumentation, slab thickness, and uniform sectioning. Postmortem human brain tissue was also obtained from the

bioRxiv preprint doi: https://doi.org/10.64898/2026.03.20.713160; this version posted March 23, 2026. The copyright holder for this preprint (which was not certified by peer review) is the author/funder, who has granted bioRxiv a license to display the preprint in perpetuity. It is made available under aCC-BY 4.0 International license.

main NBB collection ("archival donors"). Archival donors appropriate for our study were selected by NBB, in accordance with HBCAC criteria. Postmortem tissue collection followed the provisions of the United States Uniform Anatomical Gift Act of 2006 described in the California Health and Safety Code section 7150 and other applicable state and federal laws and regulations. Informed consent for unrestricted research use and open data sharing was obtained by NBB from the legal next-of-kin. The Broad Institute Office of Research Subject Protection reviewed the use of postmortem brain tissue for research purposes and determined that the use of de-identified specimens from deceased individuals did not constitute human subjects research requiring IRB review (NHSR-8066) per federal regulation 45 CFR 46 and associated guidance.

Donor information is available in **Table S2**. Adult donors ages 27 to 89+ that were consented for open data sharing were considered for this study. Exact donor age at time of death for donors over 89 years old is not available as a subject's age, in combination with other health information, could potentially be a unique identifier for individuals aged 89 and older. These subjects are listed as aged 89+ years, following HIPAA Privacy Rules. Donors with positive serology results for HIV-1/2, Hepatitis B, or Hepatitis C were not considered for this study.

We sampled from donors flagged by NBB as potentially meeting the HBCAC criteria at the time of donor intake at the brain bank. All donors were evaluated for major or persistent neurological or psychiatric conditions, such as Alzheimer's disease and schizophrenia. Toxicology testing was conducted at each contributing brain bank to determine substance presence at the time of death and to interpret substance exposure alongside clinical brain diagnoses. All available donor metadata at the time of brain bank intake were comprehensively reviewed and used to guide donor selection and initial tissue sampling.

Donors that were later found to have major or persistent neurodegenerative or psychiatric conditions, or substance dependence (including alcohol, opioid, or cocaine) were not designated as healthy control donors (see **Methods**: Overview of Outlier Filtering, **Table S1**, **S2**). Additional exclusionary criteria for healthy control designation included clinical brain diagnoses of: recurrent major depressive disorder, and other psychiatric disorders (including post-traumatic stress disorder (PTSD), obsessive-compulsive disorder (OCD), and hoarding disorder). Exclusion criteria based on neuropathological evaluation included: Cerebral infarction in donors under 65 years of age[71], Primary age-related tauopathy (PART) in donors under 65 years[72,73], Limbic-predominant age-related TDP-43 encephalopathy (LATE) in donors under 80 years[74]. Age thresholds were informed by current literature characterizing the age-related onset of these pathologies. Additional exclusionary metadata was based on causes of death, specifically: cause of death of combined overdose (OD) that includes substances of ≥2 drug classes and cause of death due to chronic substance use/abuse/dependence. Minor neurological or psychiatric conditions deemed to have minimal functional impact (e.g., migraine, specific phobias) were permitted. Donors with historical diagnoses in clinical remission were eligible, provided there was no confirmatory evidence of the disorder at the time of death per clinical records. Findings associated with normal aging or incidental focal pathology (e.g., localized ischemic events) were not considered exclusionary.

bioRxiv preprint doi: https://doi.org/10.64898/2026.03.20.713160; this version posted March 23, 2026. The copyright holder for this preprint (which was not certified by peer review) is the author/funder, who has granted bioRxiv a license to display the preprint in perpetuity. It is made available under aCC-BY 4.0 International license.

To facilitate standardized tissue selection and annotation, brain slab images from HBCAC donors were uploaded to the custom-built Neuroanatomy-anchored Information Management Platform (NIMP; RRID:SCR_024684) developed by UTHealth Houston[75]. Brain slab image collections, prepared under HBCAC protocols, consisted of an average of about 30 coronally sectioned slabs, each approximately 5 mm thick. Slabs were photographed with identification numbers and scale bars, enabling tissue requesters to estimate dimensions accurately. Neuroanatomists reviewed these slab collections and identified slabs that contained the relevant regions of interest (ROI). ROIs were drawn on the slab images to indicate the precise dissection sites and annotation pins were placed to link to neuroanatomical structures as defined in the DHBA ontology. To maintain experimental uniformity across donors and brain regions, ROIs were matched as closely as possible in anatomical position, minimizing variability caused by normal morphological differences between individuals.

Archival donors included in this study were selected by NBB staff based on the HBCAC criteria. Tissue was requested through the NBB request process and subdissections were performed by NBB in accordance with NBB's standard practice[76]. Tissue preparation varied by brain bank, with tissue arriving as small blocks, chips, or pulverized (tissue samples that were pulverized are designated as such in **Table S2**). DFC, caudate, putamen, and nucleus accumbens were sampled from the archival donors. Internal capsule tissue was not sampled for the archival donors.

RNA quality scoring

Genomic DNA and total RNA were extracted from frozen cerebellar tissue from each individual donor and processed at Broad Clinical Laboratories. RNA quality was assessed using the LabChip GX system (PerkinElmer). RNA integrity was evaluated by microfluidic capillary electrophoresis, generating RNA Quality Scores (RQS) on a scale of 1–10, with higher values indicating greater integrity. DV200 values (proportion of RNA fragments >200 nucleotides; reported on a 0–1 scale) were calculated as an additional measure of RNA quality predictive of RNA-sequencing performance. RQS and DV200 served as quantitative quality control measures.

Tissue handling and dissections

The anatomical location of each ROI was confirmed by aligning slab images to the Ding atlas[34] coordinates, with "pinned" reference points marking the anterior–posterior, dorsal - ventral, and medial - lateral positions. Care was taken to match ROIs across donors, ensuring uniform anatomical coverage despite individual morphological variation. DFC sampling targeted the cortical layers anterior to the premotor cortex and dorsal to the inferior frontal sulcus (**Figure S1A**). Striatal dissections were guided by internal landmarks to isolate the entirety of caudate nucleus, putamen, nucleus accumbens core and shell (treated as a single region for downstream analysis for consistency with archival donor sampling), and segments of the internal capsule without contamination from neighboring territories (**Figure S1B**). Dissection was conducted in a cryostat (-25 °C) using pre-chilled razor blades. Slabs were kept on dry ice until processing and immediately returned to -80 °C storage post-sampling. All cuts were made

bioRxiv preprint doi: https://doi.org/10.64898/2026.03.20.713160; this version posted March 23, 2026. The copyright holder for this preprint (which was not certified by peer review) is the author/funder, who has granted bioRxiv a license to display the preprint in perpetuity. It is made available under aCC-BY 4.0 International license.

with care to avoid inclusion of unintended subregions and any adjacent white matter, particularly for small striatal nuclei. Subdissections were conducted by NBB staff for all donors designated as “archival”.

Whole genome sequencing

Whole-genome sequencing (WGS) was performed on genomic DNA from each donor. PCR-free libraries were prepared using the KAPA HyperPrep Library Construction Kit (Roche) with custom Broad indices. Libraries were sequenced on the NovaSeq X platform (Illumina; RRID:SCR_024569) to generate 150 bp paired-end reads to a target mean coverage of 30x. Reads were demultiplexed, aggregated, and aligned to the hg38 reference genome using Illumina DRAGEN processing. Data delivery included aggregated CRAM files, corresponding CRAI index files, and md5 checksum files.

Single-nucleus RNA-seq library preparation and sequencing

To enable direct comparative analysis of nuclei from multiple brain donors while minimizing technical variance, we processed frozen brain tissue specimens from multiple donors as a single pooled sample. Each village comprised nuclei isolated from an average of 19 donors (range: 9-26 donors). For each donor, equivalent amounts of tissue (30 ± 5 mg for most regions, ~15 mg for smaller anatomical regions) were collected. All pooled samples underwent nuclei isolation, droplet-based encapsulation, library preparation, and sequencing as a single batch. Between 2-8 encapsulation reactions were performed per village, depending on the desired sequencing depth. We used combinations of hundreds of transcribed single nucleotide polymorphisms (SNPs) in each cell’s sequenced reads to assign each nucleus to its donor-of-origin, using the computational approach described below.

Nuclei were isolated from frozen brain tissue using a modified density gradient protocol[77]. Briefly, tissue blocks were cryosectioned/microdissected at −25 °C and transferred directly to pre-chilled Dounce homogenizers containing Nuclei EZ lysis buffer (MilliporeSigma, #NUC101) supplemented with 1 U/uL RiboLock RNase Inhibitor (Thermo #EO0382). Mechanical homogenization was performed with 10-20 strokes of each pestle size, followed by incubation on ice for 10 min. The lysate was passed through a 20 μm vacuum cell strainer (SCNY00020), centrifuged at 500 × g for 5 min at 4 °C, and the pellet resuspended in G30 solution (30% iodixanol (STEMCELL #07820), 3.4% sucrose, 20 mM Tricine, 25 mM KCl, 5 mM $MgCl_2$, pH 7.8). The suspension was layered over a fresh G30 cushion in 1.5 mL volumes and centrifuged at 8000 × g for 10 min at 4 °C. Supernatants were discarded, and resulting nuclei pellets were resuspended in PBSAi buffer (composition as optimized in our protocol) and pooled into a single tube per village. Nuclei suspensions were brought to 1.5 mL total volume with PBSAi, centrifuged again (500 × g, 5 min, 4 °C), and the pellets resuspended in ~100 μL of PBSAi. Nuclei were counted using a LUNA-FL Dual Fluorescence Cell Counter (Logos Biosystems, #L12005) with acridine orange/propidium iodide staining (Logos Biosystems #F23001) to assess concentration and viability.

Isolated nuclei were loaded onto a 10x Genomics Chromium instrument (RRID:SCR_024939) and processed using either Chromium NextGEM Single Cell 3’ Reagent Kits V3.1 (CG00204, Rev D) or Chromium GEM-X Single Cell 3′ Reagent Kits v4 (CG000731, Rev A) according to

bioRxiv preprint doi: https://doi.org/10.64898/2026.03.20.713160; this version posted March 23, 2026. The copyright holder for this preprint (which was not certified by peer review) is the author/funder, who has granted bioRxiv a license to display the preprint in perpetuity. It is made available under aCC-BY 4.0 International license.

the manufacturer's protocol, with minor modifications for nuclei input (19-22K loading for NextGEM, 29-32K loading for GEM-X). The majority of libraries were generated from small-scale (8-16 reactions) manual preparations using magnetic racks, while a subset of experiments were processed using automated high-throughput liquid-handling systems. Sequencing was performed on the Illumina NovaSeqX platform.

Sequence alignment and donor assignment

Sequence data was demultiplexed and aligned following the standard Drop-Seq protocol[78] and was aligned to the GRCh38 reference and GENCODE (RRID:SCR_014966) v43 gene models. The expression matrix was computed using Drop-Seq DigitalExpression program, and used two non-standard flags READ_MQ=0 and FUNCTIONAL_STRATEGY=STARSOLO to replicate STARSolo expression quantification. Ambient/ background RNA was removed from digital gene expression (DGE) matrices using CellBender (v.0.3.2) remove-background[36]. Nuclei selection was performed via DropSift (see below), then final DGE matrices were generated for those subsets of cell barcodes. These matrices were then used in combination with MapMyCells[39] to generate cell type labels. Donor assignment and doublet detection were performed by Dropulation[35].

Nucleus selection

Accurate nucleus selection is a critical step in single-nucleus RNA sequencing (snRNA-seq) analysis. Proper identification of nuclei ensures robust downstream expression quantification, cell type classification, and biological interpretation. However, distinguishing nuclei from empty droplets presents unique challenges, particularly in brain tissue, where high cellular diversity, ambient RNA contamination, and debris can complicate classification. To address these challenges, we developed DropSift, a supervised method that distinguishes nuclei from empty droplets in an experiment-specific manner.

Nucleus-containing droplets were distinguished from empty droplets using DropSift[79]. DropSift is a supervised classifier that labels barcodes as nuclei or empty droplets using a support vector machine (SVM) trained separately for each experiment. The model uses cell-level summary metrics (including total UMI counts and the fraction of intronic UMIs), expression-based features derived from genes differentially expressed between nuclei and empty droplets, and, when available, CellBender-derived estimates of ambient RNA contamination as input features. For each dataset, DropSift first identifies exemplar nuclei and empty droplets using density-based thresholds in the joint distribution of total UMI counts and intronic fraction, with additional guidance from the CellBender contamination estimates. These exemplars are used to train an SVM with a radial basis function kernel to discriminate nuclei from empty droplets. The trained classifier is then applied to all barcodes above a minimal UMI threshold; very low-UMI barcodes (<20 transcripts) which cannot be reliably classified are excluded from downstream analysis.

By integrating multiple signals, DropSift effectively differentiates nuclei from empty droplets while adapting to variability in data structure, including differences in ambient RNA contamination, across experiments and species. Rather than relying on tissue- or organism-specific marker sets, it learns expression patterns that are specific to each dataset, which improves classification accuracy in heterogeneous brain tissue.

bioRxiv preprint doi: https://doi.org/10.64898/2026.03.20.713160; this version posted March 23, 2026. The copyright holder for this preprint (which was not certified by peer review) is the author/funder, who has granted bioRxiv a license to display the preprint in perpetuity. It is made available under aCC-BY 4.0 International license.

DropSift is implemented in R and released as an open-source package. The source code, documentation, and installation instructions are available from the project GitHub repository (https://github.com/broadinstitute/DropSift)

Unsupervised clustering

Following nuclei selection to establish a set of non-empty, non-debris libraries, we performed unsupervised clustering to identify cell-type structure in the dataset. All clustering analyses were performed in R using Seurat[80] (v4.4.3; RRID:SCR_007322), with analysis-specific parameters (e.g., number of highly variable genes, number of principal components, and clustering resolution) specified below.

Raw count matrices were preprocessed using Seurat's standard single-cell workflow. Gene expression values were log-normalized using NormalizeData. Highly variable genes (HVGs) were selected using FindVariableFeatures, modeling the gene mean-variance relationship, and HVG expression values were linearly scaled using ScaleData such that each gene had zero mean and unit variance across cells. Mitochondrial (MT-) genes were excluded from clustering analyses; no additional manual gene filtering or regression was applied. Principal component analysis was performed using RunPCA on the selected HVGs. Cell-cell neighbor graphs were constructed using FindNeighbors, followed by graph-based clustering using FindClusters (Leiden algorithm). Low-dimensional embeddings were computed using UMAP via RunUMAP. To encourage co-clustering of nuclei across donors, and in some analyses across brain regions, Harmony-based integration was applied using Seurat's Harmony wrapper (implementation specified per analysis).

Due to the large scale of the dataset (~5 million nuclei when including all donors, samples, and doublets), analyses were performed using BPCells[81], an R package that enables memory-efficient storage and streaming of large single-cell expression matrices. BPCells was used in conjunction with Seurat to support normalization, dimensionality reduction, clustering, and visualization at scale.

Expression-based doublet removal

Donor multiplexing (experimental) with genotypic demultiplexing (computational) enabled identification of genotypic doublets, defined as nuclei containing genetic material from more than one donor. Experimental batches ("villages") typically included approximately 20 donors; under even donor representation, the expected proportion of heterotypic genotypic doublets is ~0.95.

To identify additional doublets based on transcriptional profiles, genotypic doublets were intentionally retained during initial clustering to serve as a reference set. Global clustering was first performed using 5,000 HVGs and 60 principal components, followed by iterative sub-clustering at higher resolution. Initial partitions separated major cell classes, including striatal neurons, cortical glutamatergic neurons, striatal GABAergic interneurons, cortical GABAergic interneurons, astrocytes / OPCs / microglia, oligodendrocytes, vascular cells,

bioRxiv preprint doi: https://doi.org/10.64898/2026.03.20.713160; this version posted March 23, 2026. The copyright holder for this preprint (which was not certified by peer review) is the author/funder, who has granted bioRxiv a license to display the preprint in perpetuity. It is made available under aCC-BY 4.0 International license.

ependymal cells, and lymphoid lineage immune cells. These groups were subsequently sub-clustered to generate terminal ("leaf") clusters corresponding to finer cell-type structure.

For each leaf cluster, the proportion of nuclei labeled as genotypic doublets was computed. Clusters in which more than 85% of nuclei were genotypic doublets were designated as doublet-enriched clusters and removed in their entirety. All other clusters were retained, resulting in removal of both genotypic doublets and transcriptionally defined doublets not identified by genotypic criteria. No additional expression-based doublet detection algorithms were applied.

This procedure yielded a curated dataset with doublet annotations and an initial set of unsupervised cell-type clusters used in downstream analyses.

Label transfer from external taxonomies

To support initial annotation and evaluation of unsupervised clustering results, cell-type labels were transferred from external reference taxonomies. For striatal populations, the consensus basal ganglia taxonomy[38] was used as the primary reference. Label transfer was performed using the MapMyCells computational framework.

For each nucleus, best-match labels were computed at multiple hierarchical levels (neighborhood, class, subclass, group, and cluster), along with bootstrap-derived confidence probabilities. Rather than relying on nucleus-level assignments, label transfer results were summarized at the cluster level and used to assign initial putative labels to unsupervised clusters.

In some samples, particularly from nucleus accumbens dissections, sub-clusters were identified that corresponded to neuronal populations outside the target regions of analysis (e.g., globus pallidus GABAergic neurons). These populations formed distinct sub-clusters and were recorded as off-target dissection artifacts in sample-level metadata; downstream analyses focused on neuronal populations within the defined target regions (CaH, Pu, NAC, and ic).

Because the dataset also included cortical nuclei (DFC) as a comparator group, some non-basal-ganglia populations were not represented in the basal ganglia taxonomy. Although MapMyCells assigned labels to these populations, these assignments were not used in downstream analyses. For cortical neuronal populations, additional label transfer was performed using structured neuronal taxonomies from the Allen Brain Atlas (RRID:SCR_017001). Annotated snRNA-seq data from human postmortem dorsolateral frontal cortex (DFC)[25] were used to define exemplar populations. scPred[82] models were trained on these reference datasets and applied to DFC-derived neuronal nuclei. Labels were assigned based on maximum predicted probability, with a minimum probability threshold of 0.8.

Sub-clustering of neuronal and glial populations

bioRxiv preprint doi: https://doi.org/10.64898/2026.03.20.713160; this version posted March 23, 2026. The copyright holder for this preprint (which was not certified by peer review) is the author/funder, who has granted bioRxiv a license to display the preprint in perpetuity. It is made available under aCC-BY 4.0 International license.

After removal of genotypic and expression-defined doublets and exclusion of donor- and sample-level CTP and GEX outliers (see **Methods**: Outlier identification and filtering), cell-type-specific sub-clustering was performed to achieve higher-resolution annotations.

The initial global clustering was sufficient to resolve certain populations (e.g., ependymal cells, B cells, and T cells), which were not further subdivided. Additional sub-clustering was performed for neuronal and major glial populations as described below. Input nuclei for sub-clustering analyses were filtered to libraries with a $\log_{10}$ UMI count equal to or greater than two median absolute deviations (MADs) below the median $\log_{10}$ UMI count for the cell type being clustered, calculated for each donor “village” to account for experimental batch effects influencing UMI counts.

Glial populations analyzed included astrocytes, oligodendrocyte precursor cells (OPCs), committed oligodendrocyte precursors (COPs), oligodendrocytes, microglia, and vascular cells. Vascular cells were sparsely sampled across donors and were excluded from downstream cell-type proportion and gene expression analyses.

Microglia were sub-clustered using 3,000 HVGs, 30 principal components, and Harmony integration by donor and brain region. A small sub-cluster of non-microglial myeloid cells annotated as border-associated macrophages (BAMs) by label transfer from the basal ganglia taxonomy was excluded from microglia analyses.

Oligodendrocytes were sub-clustered using 3,000 HVGs, 30 principal components, and Harmony integration by donor and brain region. Labels from the consensus basal ganglia taxonomy (Oligo OPALIN and Oligo PLEKHG1) were used to support annotation.

Striatal neuron sub-clustering

Striatal neuronal sub-clustering was performed to confirm representation of expected neuronal classes and to achieve high-resolution annotation. Canonical medium spiny neurons (MSNs), eccentric (“hybrid”) MSNs, and striatal interneurons were jointly sub-clustered using 5,000 HVGs, 30 principal components, and Harmony integration by donor.
Clustering was performed at resolution 0.25. Major striatal interneuron classes defined in the consensus basal ganglia taxonomy (PTHLH–PVALB GABA, TAC3–PLPP4 GABA, SST–CHODL GABA, and cholinergic GABA neurons) formed distinct clusters, supported by marker gene expression and label transfer.

Canonical MSNs separated into D1 and D2 populations. Within these populations, cells were further annotated along matrix-striosome and dorsal-ventral axes. For downstream analyses, matrix and striosome MSNs were defined by the intersection of unsupervised cluster membership (restricted to D1 or D2 clusters) and confident label transfer assignment (probability >0.8).

bioRxiv preprint doi: https://doi.org/10.64898/2026.03.20.713160; this version posted March 23, 2026. The copyright holder for this preprint (which was not certified by peer review) is the author/funder, who has granted bioRxiv a license to display the preprint in perpetuity. It is made available under aCC-BY 4.0 International license.

Astrocyte sub-clustering

Astrocytes were sub-clustered across brain regions using 3,000 HVGs, 30 principal components, and Harmony (RRID:SCR_022206) integration by donor and brain region. Clustering at resolution 0.25 yielded 11 astrocyte sub-clusters comprising ~315k nuclei. Two major clusters corresponding to striatal and cortical astrocytes collapsed at lower clustering resolutions and were combined for exploratory analyses (**Figure S18**).

As higher-resolution astrocyte annotations were not available in the consensus basal ganglia taxonomy, marker gene identification was performed using comparison of cluster pseudobulked expression profiles. For each sub-cluster, the top 20-50 genes with the largest fold changes relative to cluster 0 were selected as candidate markers. These gene sets were compared with published markers of astrocyte sub-types and states in the adult human brain [83,84] (**Figure S18B**).

A single cluster (cluster 0) accounted for the majority of astrocytes (63%) and expressed canonical homeostatic astrocyte markers, including *SLC1A2*, *KCNJ10*, *FGFR3*, and *GRM3*, consistent with a population of homeostatic “protoplasmic” astrocytes. Although this population was present across all sampled regions, there was a pronounced enrichment in gray matter regions relative to ic (**Figure S18C,D**). Cluster 1 (14%) was characterized by elevated expression of *GFAP*, *VCAN*, *TNC*, *CD44*, and extracellular matrix-associated genes, and was strongly enriched in the ic (**Figure S18C,D**), consistent with white-matter-associated “fibrous” astrocytes.

Among lower-abundance clusters, we observed evidence of regionally enriched astrocyte populations (**Figure S18B-D**). Cluster 6 (2%) showed near-exclusive enrichment in DFC samples and expressed higher levels of *DPP6*, *DPP10*, *SEMA5A*, and *DSCAML1*. This population may correspond with “interlaminar” astrocytes localized to the cortex. Cluster 3 (4%) expressed *CHRDL1*, *DIO2*, and *CALCRL* and was significantly enriched in putamen samples, potentially corresponding to a localized astrocyte population within the striatum, or astrocytes originating from an anatomically adjacent compartment resulting from off-target dissections. Cluster 5 (3%) was marked by expression of *SLC6A11* together with GABA-signaling-related genes (*GABBR2*, *RGS6*, *ADCY1*) and was enriched in NAC and ic samples. This population may reflect a recently reported *SLC6A11*+ astrocyte population localized to the globus pallidus, captured in more-posterior samples targeting the NAC.

Cluster 9 (1%) was challenging to define. Elevated expression of *AQP4* would be consistent with perivascular astrocytes, but we did not identify a larger blood-brain-barrier associated program.

Two clusters seemed to reflect astrocyte states rather than stable subtypes.

Cluster 2 (6%) expressed inflammatory and cytokine-responsive genes (*C3*, *CHI3L1*, *SERPINA3*, *CCL2*, *CXCL10*, *SOCS3*). We noted a subset of donors with elevated proportions of

bioRxiv preprint doi: https://doi.org/10.64898/2026.03.20.713160; this version posted March 23, 2026. The copyright holder for this preprint (which was not certified by peer review) is the author/funder, who has granted bioRxiv a license to display the preprint in perpetuity. It is made available under aCC-BY 4.0 International license.

astrocytes in cluster 2. One plausible interpretation is that cluster 2 represents astrocytes upregulating a “reactive” cell-state program.

Cluster 7 (2%) was distinguished by robust induction of immediate early genes (*FOS*, *JUN*, *NR4A1/2/3*) and stress-response chaperones (*HSPA1A/B*), potentially indicating an activity- or stress-associated astrocyte state. Perimortem conditions have the potential to impact expression levels of activity / stress programs, so we interpret the presence of this cluster with caution.

Two clusters likely reflect technical artifacts. Cluster 4 (4%) expressed neuronal and synapse-associated transcripts (*RBFOX1*, *SYT1*, *RIMS2*), raising the possibility of low-level neuronal RNA contamination or mixed nuclei, although a contribution from astrocytes with strong synaptic coupling cannot be excluded. Cluster 8 (2%) expressed oligodendrocyte-lineage markers (*MBP*, *PLP1*, *MOBP*, *BCAS1*) and likely represents oligodendrocyte or myelin-debris contamination.

OPC sub-clustering

OPCs were sub-clustered using 3,000 HVGs, 30 principal components, and Harmony integration by donor and brain region. Clustering at resolution 0.1 yielded eight sub-clusters, two of which collapsed at lower resolution and were combined, resulting in seven OPC sub-clusters (~155k nuclei; **Figure S9A**).

To confirm that regional integration did not obscure region-specific structure, region-specific clustering was also performed using 3,000 HVGs for CaH, Pu, and NAC and 2,000 HVGs for ic and DFC, with 30 principal components and Harmony integration by donor. The same seven OPC sub-types were recovered in each region-specific analysis. Marker genes were identified using the same pseudobulk-based approach as for astrocytes[56] (**Figure S9B**).

The resulting clusters exhibited highly skewed abundances (**Figure S9C,D**). One dominant cluster (cluster 0) accounted for approximately 84% of all OPCs, whereas the remaining clusters each comprised between 1 - 7% of the population. Despite these differences in abundance, all clusters were present to some degree across brain regions (in contrast with region-restricted sub-clusters like astrocyte cluster 6), with the possible exception of WM-localized OPCs noted below.

Cluster 0, the most abundant OPC population, expressed canonical OPC-associated genes including *BCAN*, *PCDH15*, and *ITGA8*, and lacked markers of proliferation, stress responses, or inflammatory signaling. The proportion of OPCs in this cluster was lower in the internal capsule relative to striatal GM regions; however, this pattern could be driven by the regional distribution of cluster 1 OPCs.

Cluster 1 (7%) was enriched in the internal capsule. We note several genes upregulated in cluster 1 (*CDH19*, *SEMA3E*, *GRIA4*), and several depleted in cluster 1 relative to cluster 0

bioRxiv preprint doi: https://doi.org/10.64898/2026.03.20.713160; this version posted March 23, 2026. The copyright holder for this preprint (which was not certified by peer review) is the author/funder, who has granted bioRxiv a license to display the preprint in perpetuity. It is made available under aCC-BY 4.0 International license.

(*SHISA6*, *GPC5*, *CNTN5*, *TCF7L1*). In spatial data, the former gene set marked OPCs localized to the ic and the latter marked OPCs within striatal GM regions. In contrast, genes upregulated in cluster 0 marked OPCs in all striatal regions, consistent with an OPC population localizing throughout GM and WM compartments. The ratio of cluster 1 to cluster 0 OPCs was also found to associate strongly with oligodendrocyte fraction in all regions.

Cluster 2 (3%) represented a smaller but distinct OPC population characterized by strong upregulation of *MIR137HG*, *DPYD*, and *LINC01776*, all located within the 1p21.3 genomic locus. This cluster was present across striatal and cortical brain regions, as well as across donors (**Figure S9C,D**).

Cluster 3 (2%) expressed inflammatory and cytokine-responsive genes including *OSMR*, *IL6R*, and *STAT3*. The proportion of cluster 3 OPCs varied markedly across donors and. These findings indicate that cluster 3 could represent a “reactive” OPC state linked to inflammatory signaling.

Cluster 4 (1%) was defined by expression of cell-cycle genes (*MKI67*, *TOP2A*, *CENPF*) indicating an actively mitotic OPC population. This cluster likely represents a transient cell-cycle state superimposed on other OPC identities.

Cluster 6 (1%) was marked by expression of hypoxia- and metabolism-related genes including *VEGFA*, *EPAS1*, and *SLC2A1*. Given its low abundance and transcriptional profile, this cluster may reflect a metabolic or hypoxic stress-associated OPC state, potentially influenced by local vascular environments or perimortem factors.

Finally, cluster 5 (1%) expressed mature oligodendrocyte and myelin genes (*MOG*, *MOBP*, *OPALIN*) and lacked canonical OPC markers, suggesting contamination by oligodendrocyte nuclei or ambient RNA. This cluster was excluded when interpreting OPC heterogeneity.

Visualizing expression of marker genes in Slide-tags data

To visualize the spatial distribution of expression of OPC subcluster marker genes, we utilized Slide-tags spatial transcriptomics data generated and analyzed by Kraft et al., 2026. This data is publicly available in https://assets.nemoarchive.org/collection/nemo:dat-aqicdbo. We chose to visualize gene expression in an exemplary sample (s5) that captured all of the striatal regions of interest.

Computing genetic principal components

Genetic principal components (PCs) were computed by co-embedding our samples with high-coverage whole-genome sequencing data from the 1000 Genomes Project[85] (RRID:SCR_006828) . We first restricted the 1000 Genomes VCFs to variants detected in our dataset and filtered to retain only common variants (MAF > 0.05). Sex was inferred using PLINK[86,87] (RRID:SCR_001757).

bioRxiv preprint doi: https://doi.org/10.64898/2026.03.20.713160; this version posted March 23, 2026. The copyright holder for this preprint (which was not certified by peer review) is the author/funder, who has granted bioRxiv a license to display the preprint in perpetuity. It is made available under aCC-BY 4.0 International license.

Prior to PCA, SNPs were pruned to ensure high-quality, independent markers. Specifically, we removed SNPs with extreme allele frequencies (MAF > 0.995), high missingness (>2%), excess heterozygosity (Hardy–Weinberg[88,89] equilibrium p-value < 1×10^{-6}), and autosomal SNPs showing association with sex. Long-range linkage disequilibrium (LD) regions were also excluded. PCA was performed using EIGENSOFT[90,91] smartpca (RRID:SCR_004965) on the 1000 Genomes reference panel; our samples were subsequently projected into this PC space.

Genetic ancestry proportions were estimated by fitting linear models for each of the five superpopulations as a function of the top 10 genetic principal components. Models were trained on individuals from the 1000 Genomes project with known ancestry, and predicted values with 95% confidence intervals were generated for all samples[92].

Outlier identification and filtering

Throughout this paper, various levels of outlier filtering were used for analysis (see **Table S1**). All UMAP and subtype clustering was performed using all nuclei (no filtering). Cell type proportions (CTP) analyses, including exploratory analyses of cell type abundances (fractions, ratios) and regressions with metadata, were performed on the core donor set, excluding CTP outlier samples and donors. Cell type-specific analyses, including differential expression and downstream TRADE analysis, as well as RNA-based age modeling and expression quantitative trait locus (eQTL) analyses, were performed with CTP outlier samples, CTP outlier donors, gene expression (GEX) outlier samples, GEX-cell type outlier samples, and GEX-donor outlier samples removed.

Gene expression outliers

To identify gene expression (GEX) outliers, we analyzed pseudobulked expression profiles defined at the level of region × village × donor × cell type. Detecting outliers at the cell-type level is critical for downstream cell-type-specific analyses, including differential expression and eQTL mapping. Exclusion of outlier samples can improve statistical power by increasing signal-to-noise ratios, and help minimize spurious signals arising from a small subset of atypical samples. We did not distinguish between biological and technical sources of variation, as samples that deviate strongly from the population distribution may introduce noise regardless of origin.

For each region-village-donor-cell type combination, we generated pseudobulked metacells by aggregating single-cell UMI counts. Within each brain region and cell type, samples with log-transformed library sizes less than the regional mean minus 1.96 standard deviations were removed to exclude poorly ascertained samples. After filtering for library size, remaining metacells had their gene counts normalized to counts per million (CPM) and log-transformed to stabilize variance.

Pairwise Pearson correlations were then computed between all remaining samples within each region-cell-type. For each sample, the median Pearson correlation with all other samples in the same region-cell-type was defined as a conformity score, as described in *Ling et al.*[25].

bioRxiv preprint doi: https://doi.org/10.64898/2026.03.20.713160; this version posted March 23, 2026. The copyright holder for this preprint (which was not certified by peer review) is the author/funder, who has granted bioRxiv a license to display the preprint in perpetuity. It is made available under aCC-BY 4.0 International license.

Conformity scores were modified z-score-normalized within each region-cell type, and samples with conformity z-scores less than -3.75 were classified as GEX outliers.

Outlier detection was restricted to cell types with sufficient coverage for inclusion in differential expression and eQTL analyses. Glial cell types included oligodendrocytes, astrocytes, oligodendrocyte precursor cells (OPCs), and microglia. Neuronal cell types included medium spiny neurons (MSNs) in striatal gray matter regions; because conformity scores were highly correlated across MSN subtypes, MSN D1 matrix neurons were used as a proxy for all MSNs. In dorsolateral frontal cortex (DFC), GABAergic VIP interneurons, GABAergic PVALB interneurons, and glutamatergic L2/3 intratelencephalic (L23IT) projection neurons were included.

Donors for whom more than 50% of samples were flagged as GEX outliers (based on either low library size or low conformity score) were designated as donor-level GEX outliers and excluded from selected analyses. Additionally, donors for whom more than 50% of samples for a given cell type were flagged as GEX outliers were designated as donor-cell-type GEX outliers, and only samples of that specific cell type were excluded. Donor-level filtering was applied prior to donor-cell-type-level filtering.

Cell type proportion outliers

Cell type proportion outliers were determined using a sequential filtering approach similar to the gene expression approach described above. Cell type proportion vectors were generated by aggregating and normalizing cell type counts at the subclass level (annotation_sub_class_complete) for each distinct combination of donor and village. Donor × village samples with log-transformed nuclei counts less than the region mean minus 1.96 standard deviations were removed to exclude poorly ascertained samples.

Following removal of samples with low nuclei counts, gray matter samples (from CaH, Pu, NAC, and DFC) with unexpected neuron types contributing to more than 20% of the neuronal population were identified and removed in order to exclude potential misdissections. Since the abundance of neurons is much lower in white matter, this step was not performed for internal capsule samples. Unexpected neuron types for all brain regions included the neuronal subclusters identified from outside the target regions described previously. In the striatum, any cortical intratelencephalic neurons and cortical interneurons were deemed unexpected; likewise, all MSN types were deemed out-of-distribution for DFC.

For the remaining samples, cell type proportions were transformed with the arcsine square root transformation to stabilize their variance, and conformity scores were then computed as the modified z-score of the median (within-region) pairwise Pearson correlation for each sample. Samples with a conformity score less than -3.75 were labeled as cell type proportion outliers.

Whole donors were removed from some analyses on the basis of cell type proportions either if all, or if at least 3, of their samples were flagged as outliers.

bioRxiv preprint doi: https://doi.org/10.64898/2026.03.20.713160; this version posted March 23, 2026. The copyright holder for this preprint (which was not certified by peer review) is the author/funder, who has granted bioRxiv a license to display the preprint in perpetuity. It is made available under aCC-BY 4.0 International license.

15% of overall samples were flagged as cell type proportion outliers. This varied by region – 6% of DFC samples were cell type proportion outliers, 10% of caudate samples, and about 20% of NAC, Pu, and ic. Due to the nature of their position in the striatum, nucleus accumbens, putamen, and internal capsule were most likely to be affected by dissection artifacts from adjacent regions.

Cell type proportion analyses

For each donor and brain region, we aggregated counts of all single cells with cell type annotations. These aggregated counts were then used to calculate both the proportions of individual cell types relative to the total number of cells and ratios between cell types of interests. Donors with exclusionary metadata criteria were removed from these analyses (see **Methods:** Donor selection and tissue procurement**; Table S1,S2**).

Comparisons across brain regions

To examine differences in cell type composition across brain regions, we first calculated the proportion or ratio of each cell type for each donor-region combination. For the ratio-based neuron analyses, we removed outliers with z-scores greater than 5, where z-scores were calculated separately within each brain region. We performed pairwise comparisons between regions using the Wilcoxon rank-sum test.

To assess the consistency of cell type abundances and ratios across regions within individual donors, we calculated Spearman's rank correlation coefficient for each cell type abundance of interest across all pairs of regions (n=498 tests). P-values were adjusted for multiple hypothesis testing using the Benjamini-Hochberg procedure[93]. Selected region pairs were visualized using scatterplots and heatmaps.

Quantifying variability in glial cell type proportions

Variability of glial cell type proportions was assessed separately within each brain region. For each brain-region–cell-type combination, all pairwise fold ratios between samples were computed as the ratio of the larger to the smaller proportion. The median of these pairwise fold ratios was used as a summary metric of inter-donor variability for that cell type within the specified region. Under this definition, a median fold ratio of 1.2 indicates that, for a randomly selected pair of samples, the larger proportion is typically ~20% greater than the smaller proportion.

In the caudate, median fold ratios were found to be 1.5 for oligodendrocytes, 1.6 for astrocytes, 1.4 for OPCs, and 1.9 for microglia.

Because observed variability reflects both biological and technical sources, we estimated the fraction of variability attributable to donor-level biological differences by leveraging matched samples from dorsolateral prefrontal cortex (DFC). For each glial cell type, Pearson correlations were computed between donor-level proportions in caudate and DFC. The squared correlation

bioRxiv preprint doi: https://doi.org/10.64898/2026.03.20.713160; this version posted March 23, 2026. The copyright holder for this preprint (which was not certified by peer review) is the author/funder, who has granted bioRxiv a license to display the preprint in perpetuity. It is made available under aCC-BY 4.0 International license.

coefficient (r^2) represents the fraction of caudate variance that is shared with DFC across donors.

In caudate, the shared variance (r^2) with DFC was 0.01 for oligodendrocytes, 0.18 for astrocytes, 0.22 for OPCs, and 0.28 for microglia.

To estimate the magnitude of donor-intrinsic variability, observed median fold ratios were adjusted using the cross-region correlation. Specifically, corrected fold ratios were computed as

$$F_{corrected} = \exp(r \bullet \ln(F_{obs})),$$

where F_{obs} is the observed median fold ratio and r is the Pearson correlation between caudate and DFC proportions for the given cell type. This adjustment scales the observed variability by the component that is shared across brain regions, yielding a conservative lower bound on inter-individual biological variability.

The resulting corrected median fold ratios in caudate were 1.05 for oligodendrocytes, 1.2 for astrocytes, 1.2 for OPCs, and 1.4 for microglia.

Cell type proportion regressions

We sought to evaluate age and sex effects only on cell types that were well-ascertained, with a median fraction > 1% across all samples. We additionally computed neuronal subtype proportions with respect to the total number of neurons only. We limited analysis of neurons to gray matter regions only (ic excluded). Since not all samples had OPC subcluster labels, we restricted the analysis of subcluster 0 and 1 abundances to the n=397 samples that contained at least 10 nuclei from both cluster 0 and cluster 1.

As is common for postmortem human brain tissue, the total number of nuclei recovered per sample varied substantially. Because precision in estimated cell type proportions is related to the total counts, we sought a regression framework that explicitly accounted for the underlying counts, rather than treating each measurement as equally precise. We thus modeled cell type abundance using a beta-binomial regression, which operates directly on the observed counts (number of the cell type of interest vs. number of all other cell types). In addition, because cell type proportions are constrained to the unit interval [0,1], we applied a logit link function to allow modeling of the expected (transformed) proportions on an unbounded support, enabling standard regression inference.

Fixed-effect covariates considered for downstream analysis included imputed sex, age, five genetic principal components (PC1–PC5), 10x chemistry (variable “single_cell_assay”), brain region, and biobank. We additionally considered cell-type specific single-cell quality metrics per sample: percent of reads that are intronic (variable “pct_intronic”), the fraction of UMIs removed by CellBender (variable “frac_contamination”). Donor and village were included as random effects. We quantified the contribution of each of these covariates to variance in

bioRxiv preprint doi: https://doi.org/10.64898/2026.03.20.713160; this version posted March 23, 2026. The copyright holder for this preprint (which was not certified by peer review) is the author/funder, who has granted bioRxiv a license to display the preprint in perpetuity. It is made available under aCC-BY 4.0 International license.

(logit-transformed) cell type proportions using variancePartition[94] (RRID:SCR_019204) and selected covariates that explained substantial variation.

For each cell type of interest, we fit a beta-binomial regression mixed effects model using glmmTMB[95] (RRID:SCR_025512). Donor and village were modeled as random intercepts, while the selected covariates were incorporated as fixed effects. Analysis of cortical neurons was limited to DFC, so we omitted the fixed effect of brain region.

```
Shell
 cell_type_glm <- glmmTMB(
    cbind(n_nuclei, n_other) ~ age_decades + imputed_sex +
brain_region_abbreviation_simple + mean_frac_contamination + (1 |
donor_external_id) + (1 | village)
    family=betabinomial(link="logit"),
    data = cell_type_df)
```

From these models, we extracted the coefficients corresponding to age and sex to quantify their effects on cell-type abundances while controlling for other technical and biological covariates (**Table S4**). Statistical significance was assessed using Wald tests, and p-values were adjusted for multiple comparisons across cell types and covariates of interest (age and sex; n=44 tests) using the Benjamini–Hochberg procedure, with a false discovery rate (FDR) threshold of 0.01.

Residuals were computed for each sample as the difference between the observed cell type proportion and the predicted proportion from the model based on fixed effects (age, sex, brain region, and mean contamination fraction) only.

To visualize the age effect on cell type abundance in the original proportion scale, we generated model-based predictions from the beta-binomial GLMM across the observed age range, for each brain region, while holding categorical covariates (sex) constant at the mode or continuous covariates (frac_contamination) fixed at the median values. Predictions were obtained on the response scale with random effects excluded to obtain the population-level fit, and plotted with 95% confidence intervals.

Population-level predicted cell type abundances were computed using emmeans[96] (RRID:SCR_018734). Predictions were obtained at selected ages (30 and 80 years) for each brain region, averaging over the levels of sex and fraction contamination, and 95% confidence intervals were derived on the response scale. Differences between ages were estimated using contrasts of the back-transformed predictions.

For OPC abundance (relative to all nuclei sampled), predicted proportions decreased significantly with age, from 0.0641 (95% CI: 0.0577–0.0705) at age 30 to 0.0406 (95% CI: 0.0376–0.0436) at age 80. This corresponds to an absolute difference of −0.0235 (95% CI: −0.0303 to −0.0166), an approximate 36.7% reduction in OPC abundance over fifty years.

bioRxiv preprint doi: https://doi.org/10.64898/2026.03.20.713160; this version posted March 23, 2026. The copyright holder for this preprint (which was not certified by peer review) is the author/funder, who has granted bioRxiv a license to display the preprint in perpetuity. It is made available under aCC-BY 4.0 International license.

GABAergic TAC3-PLPP4 interneuron abundance (relative to neurons only) also declined significantly with age, with predicted proportions decreasing from 0.0333 (95% CI: 0.0305–0.0361) at age 30 to 0.0265 (95% CI: 0.0249–0.0281) at age 80. This corresponds to an absolute difference of −0.0068 (95% CI: −0.0101 to −0.00351), an approximate 20.4% reduction over fifty years.

Differential expression

To detect sex-biased and age-dependent gene expression, we summed UMI counts of all assignable single cells for each distinct combination of donor, brain region, and 10x chemistry, generating a pseudobulk (observation-by-gene) matrix at the donor–region–chemistry level for each cell type. Fixed-effect covariates considered for downstream analysis included imputed sex, age, five genetic principal components (PC1–PC5), 10x chemistry (variable "single_cell_assay"), brain region, and biobank, as well as mean single-cell quality metrics per sample: percent of reads that are intronic (variable "pct_intronic"), the fraction of UMIs removed by CellBender (variable "frac_contamination"), and the z-score of the log10 number of nuclei captured (variable "z_log10_nuclei"). Additionally, donor and village are modeled as random effects.

We evaluated these potential covariates using two complementary approaches. First, we generated multidimensional scaling (MDS) plots in Glimma[97] (RRID:SCR_017389) for each cell type to visualize relationships among samples and identify variables explaining major axes of variation. This qualitative assessment highlighted covariates contributing visible structure in the data, such as donor identity, brain region, and 10x chemistry. Second, we quantified the contribution of each covariate to gene expression variance using the variancePartition[94] framework.

Based on these assessments, we selected covariates explaining substantial variation for inclusion in the dream[98] differential expression models. dream, an extension of limma-voom, fits linear mixed models with multiple random effects to account for repeated measurements. Donor and village were modeled as random intercepts, while the selected covariates were incorporated as fixed effects.

For each cell type, we filtered observations to remove donor–region–chemistry samples with low library size that were likely to contribute more noise than signal. Library size filtering was performed by computing the mean and standard deviation of total UMIs per sample and retaining only samples with at least mean − 1.96 SD total UMIs. Genes were then filtered to remove lowly expressed features. Counts were transformed to counts per million (CPM) using edgeR[99] (RRID:SCR_012802), and we retained genes where at least 10% of observations had CPM ≥ 1.

Differential expression was tested at two levels of granularity. First, to estimate the average effect of sex or age on each cell type across brain regions, we fit a region-averaged model with age as a single fixed-effect term and region included as a categorical covariate. This model was specified in dream as:

bioRxiv preprint doi: https://doi.org/10.64898/2026.03.20.713160; this version posted March 23, 2026. The copyright holder for this preprint (which was not certified by peer review) is the author/funder, who has granted bioRxiv a license to display the preprint in perpetuity. It is made available under aCC-BY 4.0 International license.

```None
~ 0 + age + z_log10_nuclei + PC1 + PC2 + PC3 + PC4 + PC5 +
    pct_intronic + frac_contamination + imputed_sex +
    single_cell_assay + region + biobank +
    (1 | donor) + (1 | village)
```

To assess region-specific heterogeneity in age effects, we fit a second model in which age effects were allowed to vary by region using region-specific age terms. Using caudate as the reference region, region-specific age effects were encoded as separate fixed-effect terms (for example, age_regionCaH, age_regionDFC). The corresponding dream formula was:

```None
~ 0 + z_log10_nuclei + PC1 + PC2 + PC3 + PC4 + PC5 +
    pct_intronic + frac_contamination + imputed_sex +
    single_cell_assay + region + biobank +
    age_regionCaH + age_regionDFC + age_regionic +
    age_regionNAC + age_regionPu +
    (1 | donor) + (1 | village)
```

This formulation allowed us to estimate region-specific age effects from a single model fit and to compare the magnitude and direction of age-associated differential expression for each gene across brain regions.

Age-associated differential expression analyses and visualizations
Age-associated differential expression was assessed using precomputed results from linear modeling frameworks treating age as a continuous variable, as described in the section on differential regression framework. Results included gene-level effect size estimates, test statistics, and FDR-adjusted significance values. Effect size uncertainties derived from model outputs were used in downstream analyses requiring standard error estimates. Analyses were performed both with brain regions combined and with region-specific models, as noted in individual analyses below.

Cell-type aging effect-size correlations

Concordance of age-associated transcriptional effects across cell types and brain regions was evaluated using pairwise comparisons of gene-level effect sizes in R using cor.test. Genes were aligned across conditions, and comparisons were restricted to genes showing evidence of age association in at least one condition (FDR < 0.05). Spearman's rank correlation was used. Correlation strength and direction were summarized using signed metrics derived from correlation coefficients. Selected comparisons were visualized using scatterplots.

bioRxiv preprint doi: https://doi.org/10.64898/2026.03.20.713160; this version posted March 23, 2026. The copyright holder for this preprint (which was not certified by peer review) is the author/funder, who has granted bioRxiv a license to display the preprint in perpetuity. It is made available under aCC-BY 4.0 International license.

Gene k-means clustering across cell types

A matrix of gene-level age-associated effect sizes estimated across all five brain regions was constructed across analyzed cell types. To arrive at a set of informative genes for this analysis we filtered to genes expressed at 10 transcripts per million (for the cell type being analyzed; in either striatum or cortex) with an FDR < 0.01 and an absolute fold change (per decade) > 1.05 in at least one cell type. This left us with 2,478 input genes. These genes were clustered based on shared age-related expression patterns using k-means clustering (stats::kmeans) with Euclidean distance, a fixed random seed (42), 200 random starts, and a maximum of 20 iterations. Effect sizes were z-score-scaled across cell types prior to clustering. The number of clusters was guided by silhouette-based evaluation (evaluating k = 10-30). Clustered gene patterns were visualized as heatmaps.

TRADE analysis by cell type

Transcriptome-wide impact of aging was quantified using TRADE[61], which integrates gene-level effect sizes and associated uncertainties to estimate global transcriptional perturbation. TRADE analyses were performed separately for each cell type using effect sizes estimated across all five brain regions. Autosomal genes were included in primary analyses, with sex chromosome genes analyzed separately. The primary outputs used in downstream analyses were the estimated transcriptome-wide impact values per cell type.

Gene set enrichment analyses

Rank-based gene set enrichment analysis was performed using fgsea (RRID:SCR_020938) in R. Genes were ranked by age-associated t-statistics. Curated gene sets from the MSigDB (RRID:SCR_016863) Human C5 collection (biological process, molecular function, and cellular compartment) were tested using permutation-based enrichment with multiple hypothesis correction. Gene sets used for visualization were selected from leadingEdge genes of statistically significant (padj < 0.01) GO categories related to the selected themes (e.g., GOBP_REGULATION_OF_SYNAPTIC_PLASTICITY, GOBP_DENDRITIC_SPINE_MORPHOGENESIS, GOBP_G_PROTEIN_COUPLED_DOPAMINE_RECEPTOR_SIGNALING_PATHWAY, GOBP_CALCIUM_ION_TRANSPORT; **Table S5**).

RNA based age prediction

Data preprocessing and feature selection

Single-cell RNA-sequencing data were aggregated to generate pseudobulk expression profiles defined at the donor × cell type × region level. For donors with multiple libraries or chemistries, raw UMI counts were summed across nuclei to yield a single expression profile per donor within each cell type and region. Only autosomal genes (chromosomes 1–22) were retained for

bioRxiv preprint doi: https://doi.org/10.64898/2026.03.20.713160; this version posted March 23, 2026. The copyright holder for this preprint (which was not certified by peer review) is the author/funder, who has granted bioRxiv a license to display the preprint in perpetuity. It is made available under aCC-BY 4.0 International license.

analysis; genes located on sex chromosomes or mitochondrial DNA were excluded prior to further filtering.

Samples with low library size were removed as described in the differential expression (DE) analysis. Genes were filtered using the same criteria applied in the DE framework, including removal of lowly expressed features based on counts per million (CPM). To restrict the feature space to age-informative genes, we retained only those genes identified as significantly associated with age (FDR ≤ 0.05) in the corresponding cell type by region–specific DE analysis. This DE-based gene selection was performed once per cell type and region using all available donors prior to model fitting and served as a predefined feature set for age prediction.

Normalization and model specification

For each modeling iteration, raw count data were transformed to log counts per million (log-CPM) using edgeR. Gene expression features were standardized by z-score transformation, computed separately for each gene using the mean and standard deviation estimated from the training donors only. These scaling parameters were then applied unchanged to the held-out donors. Test samples were provided to the model as raw count data and transformed exclusively using parameters derived from the training set, mirroring the procedure that would be used for prediction in an external dataset.

Chronological age (expressed in decades) was predicted using ridge regression, implemented as elastic net regression with $\alpha = 0$ and a Gaussian error model. At each iteration, the regularization parameter λ was selected by internal 10-fold cross-validation within the training set, minimizing mean absolute error (MAE). Model coefficients and intercepts were estimated from the training donors, and the resulting model was used to generate predictions for the held-out donors.

Repeated age-stratified cross-validation

Predictive performance was assessed using repeated age-stratified 80/20 splits (Monte Carlo cross-validation). For each cell type by region model, donors were binned into five age quantiles, and approximately 20% of donors were withheld from each bin to form a test set, preserving the overall age distribution. This procedure was repeated 200 times, drawing a new stratified split at each iteration.

For each repeat, the model was trained on 80% of donors and used to predict age in the held-out 20%. Only out-of-fold predictions were retained. After 200 iterations, each donor had multiple independent out-of-fold predictions (approximately 40 on average). Final predicted age for each donor was defined as the mean of these out-of-fold predictions. Model performance metrics, including Pearson correlation and mean absolute error, were computed from these aggregated out-of-fold predictions.

Bias correction and residual age

bioRxiv preprint doi: https://doi.org/10.64898/2026.03.20.713160; this version posted March 23, 2026. The copyright holder for this preprint (which was not certified by peer review) is the author/funder, who has granted bioRxiv a license to display the preprint in perpetuity. It is made available under aCC-BY 4.0 International license.

Across models, predicted ages exhibited regression-to-the-mean shrinkage, with younger donors predicted older and older donors predicted younger. To account for this systematic age-dependent bias, we fit a generalized additive model (GAM) for each cell type by region using aggregated out-of-fold predictions as a smooth function of chronological age. The model used a cubic regression spline (k = 5) which was estimated by restricted maximum likelihood, and employed a scaled t distribution to provide robustness to outliers.

Bias-corrected predicted age was defined by removing the estimated age-dependent deviation from the identity relationship between predicted and chronological age, thereby eliminating regression-to-the-mean effects. Residual age was defined both as the raw difference between predicted and chronological age and as the bias-corrected difference. Both forms were used in downstream analyses, with bias-corrected residual age used to assess coordination of transcriptional aging across cell types and regions.

eQTL analysis

Discovery

To generate eQTLs, we used a workflow combining parts of previous pipelines[25,100]. We summed UMI counts of all assignable single cells included in nucleus selection for each distinct combination of donor, brain region, and cell type to create pseudobulked (donor-by-gene) expression matrices at the region cell-type level. Pre-processing of expression data included summing the gene expression per gene defined by the GENCODE v43 gene models and removing the lower 50% of expressed genes, leaving 18,984 to 19,056 genes per brain region cell type matrix. Each matrix was then normalized per-donor using code derived from the pyQTL[101] implementation of edgeR CPM, then standardized using the pyQTL implementation of an inverse normal transformation (INT).

The covariates from the differential expression were used for identifying fixed effects. Probabilistic Estimation of Expression Residuals (PEER)[102,103] was used to generate 10 additional covariates for each brain region × cell type analysis.

Variants included in the analysis were from chromosomes 1-22 and X using the GRCh38 reference. Sites with a minor allele frequency (MAF) less than 0.05 or more than 0.95 were excluded from the analysis. For each variant, 90% of the donors were required to have a genotype quality greater than 30, as measured by the GQ quality tag in the VCF. Sites where the HTSJDK[104] calculated a Hardy Weinberg equilibrium (HWE)[88,89] p-value below 1e-4 were excluded.

The variant genotypes, normalized gene expression phenotypes, and set of covariates were input into tensorQTL[105] to generate cis-QTLs using its cis mode with a seed of 777, a default genome-wide false discovery rate (FDR) setting of 0.05, using a setting of one megabase window before and after each transcription start site (TSS) as defined by the GENCODE v43 gene models. For cis-QTL results reaching genome-wide significance, the tensorQTL cis_independent mode was then used to generate conditionally independent cis-QTLs[106] with

bioRxiv preprint doi: https://doi.org/10.64898/2026.03.20.713160; this version posted March 23, 2026. The copyright holder for this preprint (which was not certified by peer review) is the author/funder, who has granted bioRxiv a license to display the preprint in perpetuity. It is made available under aCC-BY 4.0 International license.

the same seed, covariates, and TSS window. A list of all pairwise variant and gene expression summary results, without genomewide FDR calculation, was generated per matrix by using the same tensorQTL inputs with the cis_nominal mode.

Donor exclusion criteria were evaluated using CTP- and gene-expression–based outlier metrics, consistent with the approach used for differential expression analyses (**Figure S10**). Data-driven filtering preserved or improved discovery yield without materially altering estimated eQTL effect sizes, whereas metadata-driven exclusions substantially reduced power (**Figure S15**).

K-means clustering

We compiled the union of SNP–gene pairs that were significant ($q < 0.01$) in at least one cell type–region combination. For each gene, we then selected a single lead cis-eQTL variant, defined as the variant with the largest absolute effect size across cell type–region combinations. Effect directions were reoriented so that the strongest effect for each gene was positive. The effect sizes of these lead variants across 17 region-stratified cell-type profiles defined the cross-context effect profile for each gene. Missing effect sizes, arising from genes not tested in specific cell types, were imputed as zero. Genes were then clustered based on these effect-size profiles using k-means.

We selected $K = 13$ as the smallest value that resolved distinct, biologically interpretable effect-size patterns without merging them. For visualization, cell types were displayed in a fixed biologically informed order (for example, MSN subtypes adjacent to each other), and clusters were arranged to group related patterns. Within clusters, genes were ordered by hierarchical clustering of effect-size profiles (correlation distance, average linkage) to highlight internal structure.

Expression fraction

To quantify the extent to which cell-type-specific eQTLs is driven by cell-type-specific gene expression, we computed median CPM expression across donors for each of the 9,899 eGenes in each of the 17 cell type-region combinations. For each gene-cell type pair, the median was computed from donors with non-NA expression for that gene; gene-cell type pairs with no expressing donors were set to zero. For each of the eight cell-type-specific K-means clusters (clusters 6-13), we identified the cell type with the highest median expression for each eGene and computed the fraction of eGenes whose highest-expressing cell type matched the cell types(s) in which the eQTLs manifested cell-type-specific regulatory effects.

Pairwise correlation

To quantify similarity of eQTL architectures across cell types, we computed pairwise Spearman correlations of cis-eQTL effect sizes between all cell-type pairs. For each pair of cell types, we included all SNP–gene pairs significant ($q < 0.01$) in at least one of the two cell types.

bioRxiv preprint doi: https://doi.org/10.64898/2026.03.20.713160; this version posted March 23, 2026. The copyright holder for this preprint (which was not certified by peer review) is the author/funder, who has granted bioRxiv a license to display the preprint in perpetuity. It is made available under aCC-BY 4.0 International license.

Correlations were assembled into a symmetric matrix and reported as squared correlation coefficients (R^2) to facilitate interpretability.

The resulting R^2 matrix was visualized as a heatmap, with hierarchical clustering applied to both rows and columns to group cell types with similar effect-size profiles.

eQTL effect size and genetic constraint

To assess whether evolutionarily constrained genes tend to have smaller eQTL effect sizes, we correlated eQTL effect sizes with LOEUF (loss-of-function observed/expected upper bound fraction) scores from gnomAD[68] v4.1. Lower LOEUF indicates stronger selective constraint against loss-of-function variants. We restricted the analysis to protein-coding genes with LOEUF scores, using canonical MANE Select transcripts.

For the cluster-level analysis, we used the same lead SNP-gene pairs as the K-means clustering (see above). For each gene, we computed the mean absolute eQTL effect size across cell types in which the eQTL reached significance (nominal p-value below the per-gene empirical threshold from tensorQTL permutations). We then computed Spearman rank correlations between LOEUF and mean effect size within each K-means cluster (K=13), with p-values corrected for multiple testing using the Benjamini-Hochberg procedure. To obtain an overall measure of the LOEUF-effect size association while controlling for cluster membership, we fit a linear regression of mean effect size on LOEUF and cluster (as a categorical covariate) and report the p-value on the LOEUF coefficient.

For the cell-type-level analysis, we used the lead SNP nominated by tensorQTL for each eGene ($q < 0.01$) independently in each of the 17 cell type-region combinations and computed Spearman correlations between LOEUF and absolute effect size per cell type, with Benjamini-Hochberg correction across cell types.
